# Closing the Carbon Loop: Continuous‐Flow Synthesis of *N*‐Aroyl Sulfoximines via Electrochemical CO_2_ Reduction

**DOI:** 10.1002/cssc.70810

**Published:** 2026-06-27

**Authors:** Giada Moroni, Maximilian Weiss, Giulia Russo, Gloria Biscontini, Olga Lanaridi, Andreas Limbeck, Tobias Huber, Alexander Karl Opitz, Katharina Bica‐Schröder

**Affiliations:** ^1^ Institute of Applied Synthetic Chemistry TU Wien Vienna Austria; ^2^ Institute of Chemical Technologies and Analytics TU Wien Vienna Austria; ^3^ Department of Chemistry Life Sciences and Environmental Sustainability University of Parma Parma Italy

**Keywords:** carbonylation reactions, CO_2_ valorization and utilization, flow chemistry, SOEC, sulfoximinocarbonylation

## Abstract

Sulfoximines have emerged as versatile scaffolds in modern synthesis, medicinal chemistry, and catalysis due to their tunable electronic and steric properties. In particular, *N*‐aroyl sulfoximines are valuable intermediates in amide coupling, C—H functionalization, and heterocycle formation. However, conventional synthetic approaches often rely on stoichiometric activating reagents and harsh conditions, generating waste and limiting substrate scope. Herein, we report a sustainable continuous‐flow platform for the Pd‐catalyzed carbonylative *N*‐aroylation of sulfoximines employing carbon monoxide (CO) as a C1 source under mild conditions. The system integrates a solid oxide electrolysis cell (SOEC) to generate dry CO from CO_2_ on‐demand, enabling a safe, modular, and atom‐economical transformation. The process displayed broad substrate tolerance, accommodating electron‐rich, electron‐poor, and heteroaromatic aryl iodides to furnish *N*‐aroyl sulfoximines in 50%–99% isolated yields within 40 min at 80°C and 6 bar. A heterogeneous Pd‐based catalyst, covalently immobilized on Merrifield resin, further enabled efficient flow operation with 96% conversion, facilitating the catalyst recovery. Finally, the adoption of Cyrene, a biomass‐derived solvent, afforded quantitative yields and demonstrated the process compatibility with green solvents. This work establishes a scalable, safe, and sustainable strategy for sulfoximine *N*‐aroylation, integrating CO_2_ valorization, continuous‐flow technology, and heterogeneous catalysis for modern carbonylation chemistry.

## Graphical abstract

A modular continuous‐flow platform enables the Pd‐catalyzed carbonylative *N*‐aroylation of sulfoximines using carbon monoxide (CO) generated on demand from carbon dioxide (CO_2_) via solid oxide electrolysis cell (SOEC). The integration of a heterogeneous palladium catalyst and a biomass‐derived solvent provides a safe, scalable, and sustainable route to *N*‐aroyl sulfoximines, advancing circular carbon utilization and key principles of green chemistry.

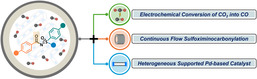

## 1 Introduction

**FIGURE 1:**
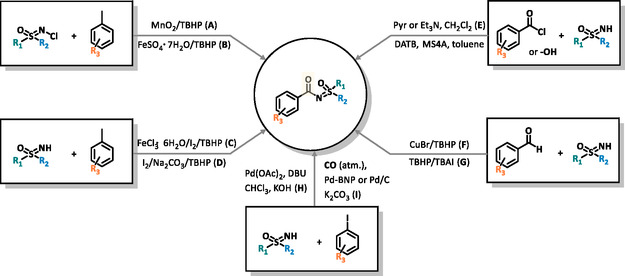
Reported synthetic approaches for aroylation of NH‐sulfoximines.

Sulfoximines and their derivatives have emerged as privileged motifs in modern organic synthesis [1, 2, 3, 4, 5, 6, 7, 8, 9], medicinal chemistry [10, 11, 12, 13, 14], and agrochemistry [15, 16]. Their unique structural framework enables extensive chemical diversification while imparting favorable physicochemical properties, thereby facilitating their incorporation into several drug candidates [17, 18, 19, 20], including tyrosine kinase inhibitors [21, 22, 23, 24, 25], as well as chiral ligands and auxiliaries for asymmetric catalysis [26, 27, 28, 29, 30, 31]. Among these, *N*‐aroyl sulfoximines represent a particularly valuable subclass due to their versatile reactivity and tunable electronic properties. They have recently attracted considerable attention as key intermediates in amide coupling reactions, as directing groups in C—H functionalization, and as precursors to *N*‐heterocycles [4, 14, 32, 33, 34, 35]. However, traditional synthetic approaches toward *N*‐aroyl sulfoximines typically rely on activated acyl chlorides or stoichiometric coupling reagents, often under strongly basic or dehydrating oxidative conditions. These methods suffer from limited substrate scope and functional group tolerance, generation of toxic waste, low selectivity or conversion, and consequently tedious purification extra steps (Figure 1A–G) [36, 37, 38, 39, 40, 41, 42, 43, 44, 45, 46, 47, 48].

Carbonylative transformations involving the insertion of carbon monoxide (CO) into metal–aryl bonds offer a more atom‐economical and elegant route to C—C, C—N, and C—O bond formation under mild and modular conditions [49, 50]. In this context, studies have demonstrated the feasibility of Pd‐catalyzed sulfoximine carbonylation using aryl halides and CO in batch mode, typically employing chemical precursors or heterogeneous Pd‐based catalysts (Figure 1H–I) [51, 52, 53]. Despite these advances, the practical use of CO in batch processes remains challenging due to its toxicity, low solubility, flammability, and difficulties associated with storage, precise dosing, safe pressurization, and handling [54, 55, 56, 57, 58, 59]. Moreover, limitations in heat and mass transfer, catalyst deactivation, poor scalability, and by‐product formation often hinder the implementation of CO‐based methodologies beyond the laboratory scale.

Continuous‐flow chemistry provides a powerful solution to conventional batch limitations by enabling safer gas–liquid transformations, enhanced control over reaction parameters, superior scalability, and reproducibility [60, 61, 62, 63, 64, 65]. Over the past decade, flow reactors have been successfully involved in catalytic carbonylative transformations facilitating the efficient incorporation of carbonyl functionalities such as amides, esters, and ketones [66, 67]. Recent developments have integrated CO utilization through membrane‐based “tube‐in‐tube” systems or chemical surrogates to minimize direct CO handling [51, 68, 69, 70, 71, 72]. In addition to reactor design, the gas–liquid flow regime critically influences mass transfer and reaction performance. Segmented flow is widely adopted for the generation of well‐defined gas–liquid slugs, ensuring efficient mixing and high interfacial area, which is suitable for fast catalytic carbonylation processes [73, 74]. Annular flow, on the other hand, provides continuous phase contact and stable gas–liquid interfaces [75], while tube‐in‐tube reactors enable controlled permeation of CO, offering enhanced safety and precise gas dosing, albeit often at longer residence times [76]. Overall, these approaches provide complementary advantages depending on the balance between mass transfer, mixing, and gas dosing criteria [77].

Beyond safety and process benefits, the integration of CO‐based carbonylation with sustainable feedstocks such as carbon dioxide (CO_2_) is becoming increasingly relevant, as it strongly aligns with current carbon recycling and green chemistry efforts [78]. However, current CO_2_‐to‐CO strategies for carbonylation remain intrinsically limited. The widely applied reverse water–gas shift (rWGSR) generates unavoidable H_2_O and residual H_2_, restricting compatibility with moisture‐sensitive catalysts and reducible substrates [79, 80, 81]. Alternative approaches, including photochemical and low‐temperature electrochemical CO_2_ reduction [82, 83, 84, 85, 86, 87, 88], suffer from slow kinetics, low current densities, catalyst aging, and contamination of the CO stream, while plasma‐based systems remain substrate specific and batch limited [89]. In contrast, solid oxide electrolysis cells (SOECs) enable high‐temperature partial electrolysis of CO_2_, delivering a dry, high‐purity, and tunable CO stream with fast kinetics, thus overcoming key bottlenecks in modern carbonylation chemistry. Herein, we report the first continuous‐flow platform for the Pd‐catalyzed *N*‐aroylation of sulfoximines using CO, operating under safe, efficient, and scalable conditions, and showcasing the successful integration of a home‐made SOEC system, recently developed within our group, for on‐demand dry CO generation [90].

## 2 Results and Discussion

Sulfoximines (**1–5**) were prepared by using a procedure according to the literature in high yields (>90%) [91]. Table 1 reports the batch optimization of base conditions for a palladium‐catalyzed carbonylative coupling reaction between 4‐iodobenzene and (methylsulfonimidoyl)benzene (**1**) as model substrates under CO atmosphere. The reaction was performed in the presence of 2 mol% Pd‐based catalyst, 0.2 eq. of ligand (*N*‐xantphos) and different bases (1 eq.) in DMF (1 mL, 0.5 M) at 80°C for 12 h as reported in the literature [53]. We systematically evaluated a range of inorganic and organic bases in order to adapt our methodology into flow conditions, examining key parameters such as basicity, nucleophilicity, and steric hindrance to obtain the desired product (**6a**). First, the reaction was performed by using K_2_CO_3_ as base and Pd/C 10% w/w as supported catalyst showing a not complete conversion (70%) under standard reaction conditions (Table 1, entry 1) [40]. The data showed that the reaction in the absence of any ligand proceeded with good conversion (70%–91%), indicating that Pd(OAc)_2_ in particular is capable of mediating the transformation but with limited efficiency (entry 2). Upon addition of *N*‐xantphos (entry 3), a substantial enhancement in reactivity was observed, with most conversions exceeding 99%, except when less effective bases such as pyridine (entry 5, 46%) and piperidine (entry 12, 78%) were used. Bases with sufficient basicity and low coordinating ability (e.g., K_2_CO_3_, Et_3_N, TBAF, DMAP, morpholine, DABCO) promoted nearly quantitative conversions (entries 3–4, 6–11, and 13). These bases likely assist in deprotonating reaction intermediates and neutralizing acidic byproducts (e.g., HI), thus enabling efficient turnover of the Pd(0)/Pd(II) catalytic cycle. In contrast, weaker or more coordinating bases such as pyridine and piperidine (entries 5 and 12) may inhibit catalysis by coordinating to palladium centers, thereby decreasing the availability of the active catalyst. Overall, the data indicate that base selection has a significant impact on catalytic efficiency, with moderately basic and weakly coordinating bases being most effective under these carbonylation conditions.

**TABLE 1:** Batch mode screening of organic bases for palladium‐catalyzed *N*‐aroylation of sulfoximines.^a^

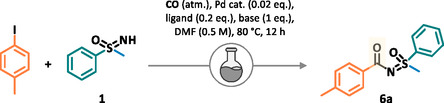				
Entry	Catalyst	Ligand	Base	Conversion, %^b^
1	Pd/C	—	K_2_CO_3_	70
2	Pd(OAc)_2_	—	K_2_CO_3_	91
3	Pd(OAc)_2_	*N*‐xantphos	K_2_CO_3_	>99
4	Pd(OAc)_2_	*N*‐xantphos	DIPEA	>99
5	Pd(OAc)_2_	*N*‐xantphos	Pyridine	46
6	Pd(OAc)_2_	*N*‐xantphos	DBU	>99
7	Pd(OAc)_2_	*N*‐xantphos	Et_3_N	>99
8	Pd(OAc)_2_	*N*‐xantphos	TBAF	>99
9	Pd(OAc)_2_	*N*‐xantphos	Pyrrolidine	>99
10	Pd(OAc)_2_	*N*‐xantphos	DMAP	>99
11	Pd(OAc)_2_	*N*‐xantphos	Morpholine	>99
12	Pd(OAc)_2_	*N*‐xantphos	Piperidine	78
13	Pd(OAc)_2_	*N*‐xantphos	DABCO	>99

a All reactions were performed with 0.5 mmol of 4‐iodotoluene (1 eq.) and (methylsulfonimidoyl)benzene (**1**, 1.5 eq.) in 1 mL of DMF for 12 h at 80°C under CO atmospheric pressure.

b Determined by ^1^H‐NMR of the crude reaction mixture by comparing the methyl of tolyl group (3H, s) of **6a** (2.40 ppm) with the methyl group of 4‐iodotoluene (2.29 ppm) of the reaction mixture or aromatic protons in *α*‐positions of tolyl groups (2H, m) (7.23–7.18 for **6a** and 6.94–6.90 ppm for 4‐iodotoluene, respectively).

**SCHEME 1:**
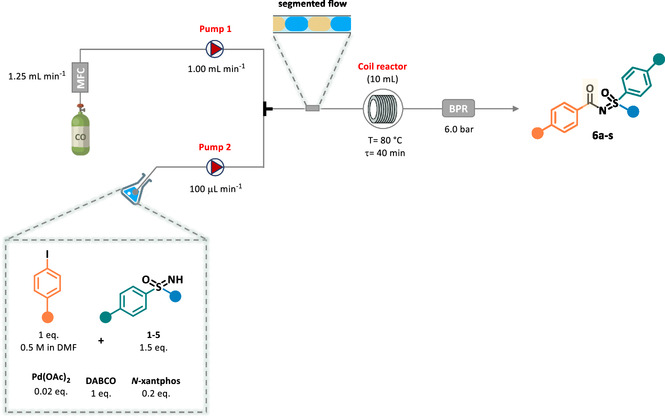
General flow setup for palladium‐catalyzed *N*‐aroylation of sulfoximines.

Having identified the optimized reaction conditions, the transformation was successfully implemented in a continuous‐flow system. The feed solution containing aryl halides (1 eq.), sulfoximines (**1–5**, 1.5 eq.), Pd(OAc)_2_ (2 mol%), *N*‐xantphos (0.2 eq.), and base (1 eq.) was dissolved in DMF (1 mL, 0.5 M) and pumped at 100 μL min^−1^, mixed with a CO stream at 1.00 mL min^−1^ controlled by a mass flow controller (MFC, 1.25 mL min^−1^) and generating a segmented flow. The reaction was realized inside a 10 mL coil reactor (Vapourtec E‐Series easy‐MedChem) heated at 80°C and pressurized at 6 bar for 40 min (Scheme 1).

Flow‐mode optimization allowed us to identify 1,4‐diazabicyclooctane (DABCO) as the optimal base ensuring complete conversion in short reaction times, from 12 h to just 40 min, while achieving excellent yields for a variety of functionalized sulfoximines (**1–5**) and commercial aryl iodides. The flow translation through efficient heat and mass transfer advances led to consistent reaction performance across a broad range of substrates. The results summarized in Table 2 illustrate the wide substrate tolerance, including electron‐rich, electron‐poor, and heteroaromatic aryl iodides, obtaining the final compounds (**6a–s**) in good to excellent yields under relatively short residence times and mild reaction conditions. Electron‐neutral and electron‐donating substrates (e.g., **6a–f**) afforded complete conversions and excellent isolated yields (83%–99%) at reduced residence times due to their ability to facilitate the oxidative addition step of catalytic cycle, owing to increased electron density on the aryl iodide moiety. Under continuous‐flow conditions, the target compound **6a** was successfully scaled up with a productivity of 7.1 mmol h^−1^, corresponding to 171 mmol day^−1^, based on a 95% isolated yield from 5 mmol of starting material and a residence time of 40 min. In contrast, halogenated and electron‐withdrawing substrates such as **6g–j** gave slightly lower yields due to the lower electron density on the aryl iodide that slows oxidative addition to Pd(0). The chlorinated derivatives **6g** and **6h** showed only a modest drop in reactivity, reflecting the moderate ^–^I and weak ^+^M effects of chlorine group. The fluorinated compound **6j**, although more deactivating, still provided good yield under mild flow conditions. Notably, the incorporation of fluorine group on compound **6j** represents a common strategy in medicinal chemistry to modulate lipophilicity, metabolic stability, and binding affinity. Heteroaromatic substrates **6k–n** derived from corresponding halides further demonstrated the broad applicability of the method. The formation of thiophene derivative **6k** proceeded efficiently (77%), highlighting the exceptional tolerance of the catalytic system toward sulfur‐containing heterocycles, which are notoriously prone to catalyst deactivation under conventional Pd‐carbonylation conditions. The naphthyl substrate **6l** also provided moderate yields (54%), showing that extended π‐conjugation and steric hindrance could interfere with the catalytic steps. The pyridyl derivative **6m**, not previously synthesized in the cited literature, was obtained in good yield (71%), probably related with the possible Pd coordination to the nitrogen atom, which may transiently deactivate the catalyst. Meanwhile, the 3‐chloropyridine analog **6n** was isolated in moderate yield (50%), likely due to the combined steric and electronic inductive effects of the chlorine substituent. However, this compound represents a valuable synthetic intermediate for the preparation of alkynyl derivatives, known as tyrosine kinase inhibitors [22, 24, 25, 92, 93, 94]. Compounds bearing functional handles for further derivatization were also tolerated. The nitrile derivative **6o** (65%) and nitro compound **6p** (79%) can be employed as precursors to primary amines through reduction, offering access to valuable intermediates for medicinal chemistry and/or subsequent cross‐coupling reactions. The acetyl‐protected substrate **6q** (66%) was successfully isolated without hydrolysis, in contrast to previous reports where complete deprotection occurred under K_2_CO_3_‐mediated conditions [53]. This stability can be ascribed to the milder basicity of DABCO. Notably, the introduction of the acetyl moiety offers a dual advantage: it may operate as a prodrug motif, enabling gradual in vivo hydrolysis to release the active compound, or as a versatile synthetic intermediate for subsequent functionalization. Similarly, the methyl ester derivative **6r** was obtained in good yield (68%). This substrate provides a valuable synthetic handle for subsequent amidation or hydrolysis transformations, allowing access to carboxamide or alcohol analogs. Conversely, the acetophenone derivative **6s** afforded a significantly lower yield (20%), likely due to the coordination of the carbonyl moiety to the palladium center, which may inhibit catalytic turnover. Despite its lower yield, **6s** remains synthetically interesting as potential precursor for reductive amination strategies, providing access to secondary amines of pharmacological interest.

**TABLE 2:** Substrate scope for palladium‐catalyzed *N*‐aroylation of sulfoximines (**6a–s**).^a^, ^b^

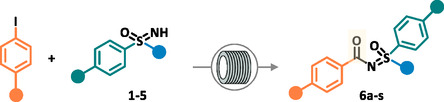	
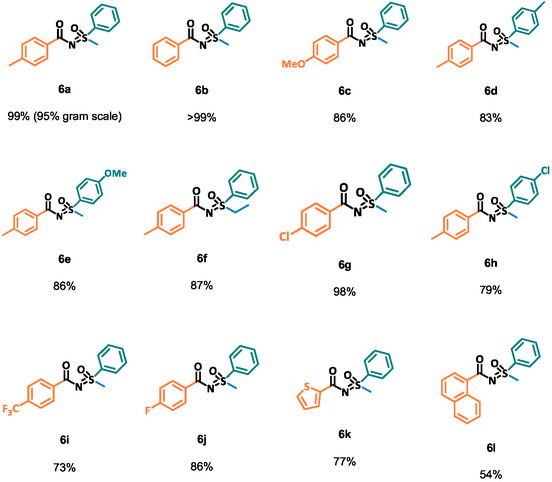	
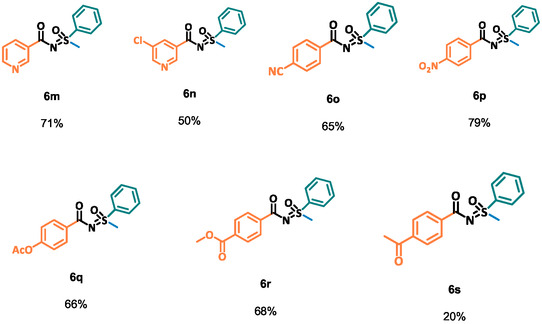	

a All reactions were performed with 0.5 mmol of 4‐iodotoluene (1 eq.), (methylsulfonimidoyl)benzene (**1**, 1.5 eq.), DABCO (1 eq.), Pd(OAc)_2_ (0.02 eq.), and *N*‐xantphos (0.2 eq.) in 1 mL of DMF. The reaction mixture was pumped at 100 μL min^−1^, mixed with a stream of CO at 1.00 mL min^−1^, and reacted inside a 10 mL coil reactor for 40 min at 80°C and 6 bar (BPR).

b Isolated yield.

To better understand and optimize the system, the actual equivalents of CO were calculated using the ideal gas law (Equation (1)), considering pressure, temperature, and volume. The CO volume was determined as the product of the mass flow controller (MFC) flow rate and the residence time (*τ*). The reaction was performed at 6 bar (≈5.92 atm) and 80°C (353 K). Based on these conditions, the amount of CO used corresponds to approximately 20 eq. required for full conversion of the model compound **6a** under standard conditions.

(1) $$n\mathrm{CO}=\frac{P\left(\mathrm{atm}\right)V(L)}{R(L\cdot \mathrm{atm}\cdot {\mathrm{mol}}^{-1}\cdot K^{-1})T(K)}$$

**SCHEME 2:**
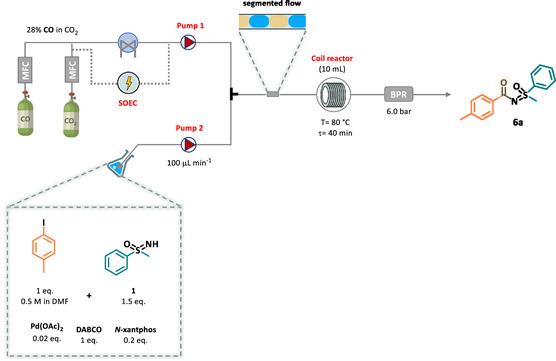
General flow setup for palladium‐catalyzed *N*‐aroylation of sulfoximines by using CO/CO_2_ gas mixture.

To establish the optimal operating conditions for the in‐house SOEC described in detail in our recent publication [77], the CO/CO_2_ ratio was determined ex situ by using two MFCs to precisely tune CO/CO_2_ delivery (Scheme 2). Using a CO MFC flow rate of 1.25 mL min^−1^ and a residence time of 40 min, quantitative conversion was expected. The flow rate of pump 1 was set to maintain the same ratio relative to the MFC flow considering that CO constitutes 28% of the CO_2_ stream. Under these optimized conditions, full conversion was achieved (Table 3 **,** entry 3). Subsequently, the reaction was performed using half the CO flow rate, corresponding theoretically to 10 eq. However, the residence time was also halved, resulting in only 5 effective eq. of CO, and conversion decreased to 90%–92% (entry 1). Finally, a CO MFC flow rate of 0.93 mL min^−1^ (≈11 eq.) with a 30 min residence time afforded 99% conversion (entry 2). These results demonstrate that both reaction time and the amount of CO could be significantly reduced while maintaining high performance.

**TABLE 3:** CO/CO_2_ gas mixture by using two MFCs.^a^

Entry	Pump 1 flow rate, mL min^−1^	MFC CO flow rate, mL min^−1^	MFC tot flow rate, mL min^−1^	Residence time, *τ*, min	CO, eq.^b^	Conversion, %^c^
1	1.80	0.62	2.22	20	5	90–92
2	2.40	0.93	3.33	30	11	99
3	3.60	1.25	4.45	40	20	>99

a All reactions were performed with 0.5 mmol of 4‐iodotoluene (1 eq.), (methylsulfonimidoyl)benzene (**1**, 1.5 eq.), DABCO (1 eq.), Pd(OAc)_2_ (0.02 eq.), and *N*‐xantphos (0.2 eq.) in 1 mL of DMF. The reaction mixture was pumped, mixed with a stream of CO in CO_2_ at variable flow rates (28% of CO in CO_2_), and reacted inside a 10 mL coil reactor at 80°C and 6 bar (BPR).

b Determined according to the equation *n*CO = *P* (atm) *V* (L)/*RT* (K).

c Determined by ^1^H‐NMR of the crude reaction mixture by comparing the methyl of tolyl group (3H, s) of **6a** (2.40 ppm) with the methyl group of 4‐iodotoluene (2.29 ppm) of the reaction mixture or aromatic protons in *α*‐positions of tolyl groups (2H, m) (7.23–7.18 for **6a** and 6.94–6.90 ppm for 4‐iodotoluene, respectively).

**FIGURE 2:**
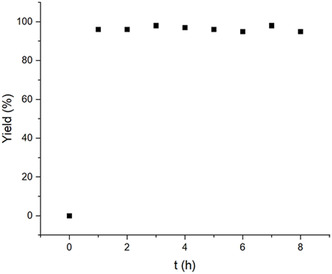
Long‐term stability experiment: yield (%) versus time (h). Reaction conditions: the reaction was performed by using 4‐iodotoluene (2.50 g, 11.5 mmol, 1 equiv.) in DMF (0.11 M) at 100 μL min^−1^ (pump B), mixed with a CO/CO_2_ stream (3.60 GmL min^−1^) controlled by a MFC (8.00 mL min^−1^). Pump C was connected to SOEC (750°C, 0.288 A, 2.0 V, corresponding to 28% CO). The reaction mixture was reacted inside a 10 mL coil reactor (total residence time: 480 min) at 80°C and 6 bar (BPR, pump A). Yields were determined by qNMR using CH_2_Br_2_ as internal standard.

With all key parameters optimized, a custom‐designed SOEC was integrated online with the flow setup, enabling a streamlined continuous process for CO generation and utilization with tunable composition. The SOEC features a specialized cathode with an electrochemically active zone made from pure Gd‐doped CeO_2_, ideally suited for dry CO_2_ electrolysis (see Section S3) [90, 95]. Under the same conditions as the above‐mentioned ex situ syntheses and a gas output of ≈8 mL min^−1^ (controlled by MFC), a CO content of 14% in CO_2_ (SOEC parameters: 0.148 A, 1.48 V) delivered a conversion of 95% and 87% yield, as determined by qNMR analysis. Notably, increasing the CO content to 28% (0.288 A, 2.0 V) resulted in quantitative conversion and yield within 40 min, fully consistent with results previously obtained using two MFCs (see Section S3.1). Long‐term stability of the integrated SOEC‐flow system was evaluated under optimized conditions (CO content 28%; 0.288 A, 2.0 V). The system was operated continuously for 8 h with fractions collected at hourly intervals. All crude samples were analyzed by qNMR monitoring the reaction yield over time. Consistent results were obtained throughout the experiment, confirming the stable performance of the system under prolonged continuous operation (Figure 2).

To intensify the process, we developed a heterogeneous Pd‐based catalyst (**7**, see Section S4.1) as a sustainable strategy to overcome homogeneous catalysis drawbacks, such as high cost, difficulties in recycling, and complex purification, while facilitating the streamline integration with continuous‐flow technology. For this purpose, the heterogeneous Pd‐based catalyst (**7**) was prepared by using the commercial Merrifield resin as the solid support. The synthesis involved the covalent immobilization of *N*‐xantphos as the ligand onto chlorinated Merrifield resin in anhydrous THF under reflux using NaH as base (step 1) [96]. The resulting grayish supported ligand (**8**) was then treated with Pd(OAc)_2_ in anhydrous THF at 25°C, yielding the target heterogeneous Pd‐based catalyst (**7**) as a black resin (step 2). The synthetic procedures are reported in detail in the Supporting Information.

Catalyst **7** was characterized via ICP analysis (Pd loading = 1.0688 mmol g^−1^) and tested in both batch and continuous‐flow conditions using a packed‐bed column (see Sections S4.2 and S4.3). Initially, the reaction was investigated by using Pd/C 10% w/w as supported catalyst, diluted with celite (1:19 w/w) as reported in the literature [53]. Catalyst packing strongly influenced residence time, requiring careful adjustment of solvent and gas flow rates. Under optimized conditions, the reaction reached ∼60% conversion in short residence time (Table 4, entry 1). In contrast, the heterogeneous catalyst **7** exhibited excellent performance, achieving quantitative conversion in batch and 96% conversion under flow mode (entries 2–3). Extending residence time by halving the flow rate did not further improve conversion (entry 4). Catalyst retention and potential Pd leaching were further investigated using an alternative diluent. Replacing celite with high‐loading Merrifield resin, which swells significantly in DMF (≈3.4× its original volume), led to a drop in conversion to 29% (entry 5). The catalyst recovered after the continuous‐flow experiment was also analyzed by ICP, showing a Pd loading of 0.8370 mmol g^−1^. This indicates a partial loss of Pd; however, the catalytic activity remained high for short‐term experiments under continuous‐flow conditions with a conversion of 96% (Table 4, entry 3). Further studies on the long‐term stability of the heterogeneous catalytic system under continuous‐flow conditions were performed for 8 h using the integrated electrolysis setup. Reaction fractions were collected hourly and analyzed by ICP. Results showed a gradual decrease in conversion over time with constant leaching of Pd into the product fraction. These findings suggest that further optimization of the immobilized catalyst and its compatibility with the selected solvents is required. A more detailed investigation of the Pd leaching pathways will be addressed in future work.

**TABLE 4:** Preliminary experiments by using supported Pd‐based resin as heterogeneous catalyst (**7**).^a^

Entry	Conditions	Cat.	Diluent	Pump CO flow rate, mL min^−1^	Pump solvent flow rate, mL min^−1^	Residence time, *τ*, min	Conversion, %^b^
1	Flow	Pd/C	Celite	0.50	0.80	2	60
2	Batch	**7** c	—	—	—	720	>99
3	Flow	**7**	Celite	0.50	0.80	2	96
4	Flow	**7**	Celite	0.25	0.40	4	96
5	Flow	**7**	Merrifield resin HL^d^	0.50	0.80	2	29

a All reactions were performed with 0.5 mmol of 4‐iodotoluene (1 eq.) in 1 mL of DMF. The standard conditions were modified by increasing the solvent and gas flow rates; otherwise, the column would have unpacked. A tubular reactor (DIBA 1.6 × 150 mm) was packed with the catalyst (0.1 eq.) and diluted with solid support (950 mg for celite and 600 mg for Merrifield resin, 1:19 and 1:13 w/w, respectively). The reaction mixture was carried out at 80°C and 6 bar (BPR). The crude was basified with NaOH (0.1 M) for removing the excess of sulfoximine and extracted with Et_2_O.

b Determined by ^1^H‐NMR of the crude reaction mixture by comparing the methyl of tolyl group (3H, s) of **6a** (2.40 ppm) with the methyl group of 4‐iodotoluene (2.29 ppm) of the reaction mixture or aromatic protons in *α*‐positions of tolyl groups (2H, m) (7.23–7.18 for **6a** and 6.94–6.90 ppm for 4‐iodotoluene, respectively).

c 0.02 eq. of **7** was used under batch conditions.

d Swelling phenomena were observed in DMF (≈3.4× its original volume).

Despite this optimization, DMF remains a drawback as a solvent due to its high toxicity, although it efficiently dissolves all reaction components, including the reaction products. In line with green chemistry principles, dihydrolevoglucosenon (commercially known as Cyrene) was evaluated as a sustainable solvent alternative [97, 98]. The reaction was first tested on the model substrates in batch at 80°C for 12 h, affording 94% conversion. Encouraged by this result, the reaction was translated into flow system. Although conversion was quantitative, product precipitation occurred at the reactor outlet junction with the BPR. The issue was solved by immersing the reactor outlet in an ultrasonic bath, which prevented blockage and allowed quantitative conversion after Et_2_O washings. The use of a cosolvent or an online dilution strategy will be investigated for future developments.

## 3 Conclusion

A continuous‐flow Pd‐catalyzed carbonylative *N*‐aroylation of sulfoximines has been developed combining safety, sustainability, and efficiency. The flow setup enables precise control of CO dosage, superior mass and heat transfer, and reduced reaction times compared to batch operation. Integration of a SOEC provides a sustainable route to on‐demand CO generation from CO_2_, aligning with green chemistry and circular carbon principles. The identification of DABCO as an optimal base ensured nearly quantitative conversions across a broad substrate scope, while the development of a heterogeneous Pd‐based catalyst enhanced recyclability and operational robustness. Furthermore, successful implementation in Cyrene, a renewable solvent, underscores the environmental compatibility of the process. Overall, this study demonstrates a modular and scalable platform that combines continuous‐flow technology, CO_2_‐derived carbonyl feedstock, and heterogeneous catalysis to access *N*‐aroyl sulfoximines efficiently providing a model for sustainable carbonylation chemistry in pharmaceutical and fine chemical manufacturing.

## Funding

This work was supported by the European Research Council (864991), Austrian Science Fund (COE5 and I5478).

## Conflicts of Interest

The authors declare no conflicts of interest.

## Supporting information

Supplementary Material

## Data Availability

The data that support the findings of this study are available in the supplementary material of this article.

## References

[1] Q.‐M. Pu , K.‐J. Jiao , X.‐T. Gao , and S.‐W. Chen , “Recent Advances in Electrochemical Transformation of Sulfoximines,” Tetrahedron 193 (2026): 135139.

[2] Q. Zeng and A. A. Nking’wa , eds., In Sulfoximines: Synthesis, Transformation and Applications (Springer, 2025): 55–112.

[3] Z. W. Boyer , N. Y. Kwon , and J. A. Ellman , “Ruthenium‐Catalyzed Enantioselective Alkylation of Sulfenamides: A General Approach for the Synthesis of Drug Relevant S‐Methyl and S‐Cyclopropyl Sulfoximines,” Journal of American Chemical Society 147 (2025): 147. 10.1021/jacs.5c03841 PMC12083213 40289728

[4] M. Andresini , A. Tota , L. Degennaro , J. A. Bull , and R. Luisi , “Synthesis and Transformations of NH‐Sulfoximines,” Chemistry – A European Journal 27 (2021): 17293–17321. 34519376 10.1002/chem.202102619 PMC9291533

[5] S. Wiezorek , P. Lamers , and C. Bolm , “Conversion and Degradation Pathways of Sulfoximines,” Chemical Society Reviews 48 (2019): 5408. 31535112 10.1039/c9cs00483a

[6] L. Degennaro , A. Tota , S. De Angelis , et al., “A Convenient, Mild, and Green Synthesis of NH‐Sulfoximines in Flow Reactors,” European Journal of Organic Chemistry 2017 (2017): 6486.

[7] N. Duchemin , R. Buccafusca , M. Daumas , V. Ferey , and S. Arseniyadis , “Facile, Stereoselective Preparation and Pd‐Catalyzed Suzuki‐Miyaura Cross‐Coupling of Alkenyl Sulfoximine,” Chemrxiv: the Preprint Server for Chemistry (2019), 10.26434/chemrxiv.8091314.v1.

[8] V. Bizet , C. M. M. Hendriks , and C. Bolm , “Sulfur Imidations: Access to Sulfimides and Sulfoximines,” Chemical Society Reviews 44 (2015): 3378. 25941981 10.1039/c5cs00208g

[9] H. Okamura and C. Bolm , “Sulfoximines: Synthesis and Catalytic Applications,” Chemistry Letters 33 (2004): 482.

[10] P. I. Arvidsson , “Sulfilimines: An Underexplored Bioisostere for Drug Design?,” Journal of Medicinal Chemistry 68 (2025): 4056. 39887148 10.1021/acs.jmedchem.5c00195 PMC11874043

[11] M. Skóra , M. Nowacki , and M. Dawidowski , “Sulfoximine Derivatives—Their Pharmacochemical Properties, Synthesis, and Potential in Drug Discovery,” Prospects in Pharmaceutical Sciences 24, no. 2 (2025): 104, 10.56782/pps.626.

[12] U. Lücking , “New Opportunities for the Utilization of the Sulfoximine Group in Medicinal Chemistry from the Drug Designer's Perspective,” Chemistry – A European Journal 28 (2022): e202201993. 35789054 10.1002/chem.202201993

[13] Y. Han , K. Xing , J. Zhang , et al., “Application of Sulfoximines in Medicinal Chemistry from 2013 to 2020,” European Journal of Medicinal Chemistry 209 (2021): 112885. 33227576 10.1016/j.ejmech.2020.112885

[14] P. Mäder and L. Kattner , “Sulfoximines as Rising Stars in Modern Drug Discovery? Current Status and Perspective on an Emerging Functional Group in Medicinal Chemistry,” Journal of Medicinal Chemistry 63 (2020): 14243. 32870008 10.1021/acs.jmedchem.0c00960

[15] T. C. Sparks , G. B. Watson , M. R. Loso , C. Geng , J. M. Babcock , and J. D. Thomas , “Sulfoxaflor and the Sulfoximine Insecticides: Chemistry, Mode of Action and Basis for Efficacy on Resistant Insects,” Pesticide Biochemistry and Physiology 107 (2013): 1. 25149228 10.1016/j.pestbp.2013.05.014

[16] J. Park , H. Baars , S. Mersmann , et al., “N‐Cyano Sulfoximines: COX Inhibition, Anticancer Activity, Cellular Toxicity, and Mutagenicity,” ChemMedChem 8 (2013): 217. 23225780 10.1002/cmdc.201200403

[17] J. A. Sirvent and U. Lücking , “Novel Pieces for the Emerging Picture of Sulfoximines in Drug Discovery: Synthesis and Evaluation of Sulfoximine Analogues of Marketed Drugs and Advanced Clinical Candidates,” ChemMedChem 12 (2017): 487. 28221724 10.1002/cmdc.201700044 PMC5485063

[18] U. Lücking , R. Jautelat , M. Krüger , et al., “Siemeister (Bayer Pharma AG), “The Lab Oddity Prevails: Discovery of Pan‐CDK Inhibitor (R)‐S‐Cyclopropyl‐S‐(4‐{[4‐{[(1R,2R)‐2‐Hydroxy‐1‐Methylpropyl]oxy}‐5‐(trifluoromethyl)pyrimidin‐2‐Yl]amino}phenyl)sulfoximide (BAY 1000394) for the Treatment of Cancer,” ChemMedChem 8 (2013): 1067. 23671017 10.1002/cmdc.201300096

[19] U. Lücking , A. Scholz , P. Lienau , et al., “Identification of Atuveciclib (BAY 1143572), the First Highly Selective, Clinical PTEFb/CDK9 Inhibitor for the Treatment of Cancer,” ChemMedChem 12 (2017): 1776. 28961375 10.1002/cmdc.201700447 PMC5698704

[20] A. Scholz , U. Lücking , G. Siemeister , et al., “Abstract 3022: BAY 1143572, a First‐in‐Class, Highly Selective, Potent and Orally Available Inhibitor of PTEFb/CDK9 Currently in Phase I, Shows Convincing Anti‐Tumor Activity in Preclinical Models of Acute Myeloid Leukemia,” Cancer Research 76 (2016): 3022.

[21] H. Xu , H. Zhang , S. Jia , et al., “Diethenyl Sulfoximine (DESI) as an Irreversible Lysine‐Targeting Warhead Enables the Design of Covalent Allosteric EGFR Inhibitor,” Chemistry ‐ A European Journal 31 (2025): e202501389. 40552597 10.1002/chem.202501389

[22] J. A. Wurster , E. C. Hull III , R. D. Yee , S. Boral , T. C. Malone , and S. Wang , WO 2022094218A1 (Allergan, Inc., 2022).

[23] D. P. Walker , M. P. Zawistoski , M. A. McGlynn , et al., “Sulfoximine‐Substituted Trifluoromethylpyrimidine Analogs as Inhibitors of Proline‐Rich Tyrosine Kinase 2 (PYK2) Show Reduced hERG Activity,” Bioorganic & Medicinal Chemistry Letters 19 (2009): 3253. 19428251 10.1016/j.bmcl.2009.04.093

[24] J. A. Wurster , E. C. Hull III , R. D. Yee , S. Boral , T. C. Malone , and S. Wang , US 2009/0196906A1 (Allergan, Inc., 2009).

[25] J. A. Wurster , E. C. Hull III , R. D. Yee , S. Boral , T. C. Malone , and S. Wang , WO 2008/061236A2 (Allergan, Inc., 2008).

[26] M. Suleman , T. Huang , T. Zhou , Z. Chen , and B. Shi , “Recent Advances in Asymmetric Synthesis of Chiral‐at‐Sulfur Sulfoximines,” ACS Catalysis 15 (2025): 5511.

[27] S. Teng , Z. P. Shultz , C. Shan , L. Wojtas , and J. M. Lopchuk , “Asymmetric Synthesis of Sulfoximines, Sulfonimidoyl Fluorides, and Sulfonimidamides Enabled by an Enantiopure Bifunctional S(VI) Reagent,” Nature Chemistry 16 (2024): 183. 10.1038/s41557-023-01419-3 PMC11000591 38238465

[28] S. Sau , K. Mukherjee , K. Kondalarao , and V. Sahoo , “Probing Chiral Sulfoximine Auxiliaries in Ru(II)‐Catalyzed One‐Pot Asymmetric C–H Hydroarylation and Annulations with Alkynes,” Organic Letters 25 (2023): 7667. 37844260 10.1021/acs.orglett.3c02969

[29] G. Proietti , J. Kuzmin , A. Z. Temerdashev , and P. Dinér , “Accessing Perfluoroaryl Sulfonimidamides and Sulfoximines via Photogenerated Perfluoroaryl Nitrenes: Synthesis and Application as a Chiral Auxiliary,” Journal of Organic Chemistry 86 (2021): 17119. 34766772 10.1021/acs.joc.1c02241 PMC8650101

[30] S. Otocka , M. Kwiatkowska , L. Madalińska , and P. Kiełbasiński , “Chiral Organosulfur Ligands/Catalysts with a Stereogenic Sulfur Atom: Applications in Asymmetric Synthesis,” Chemical Reviews 117 (2017): 4147. 28191933 10.1021/acs.chemrev.6b00517

[31] H.‐J. Gais , “Development of New Methods for Asymmetric Synthesis Based on Sulfoximines,” Heteroatom Chemistry 18 (2007): 472.

[32] H. A. E. Adam , S. Zhou , and Q. Zeng , “Advances in Cross‐Coupling and Oxidative Coupling Reactions of NH‐Sulfoximines – A Review,” Chemical Communications 61 (1934): 2025. 10.1039/d4cc05308g 39757832

[33] J. A. Bull , L. Degennaro , and R. Luisi , “Straightforward Strategies for the Preparation of NH‐Sulfoximines: A Serendipitous Story,” Synlett 28 (2017): 2525.

[34] P. Ghosh , B. Ganguly , and S. Das , “N‐H and C‐H Functionalization of Sulfoximine: Recent Advancement and Prospects,” Asian Journal of Organic Chemistry 9 (2035): 2020.

[35] M. Reggelin and C. Zur , “Sulfoximines: Structures, Properties and Synthetic Applications,” Synthesis 2000 (2000): 1.

[36] D. L. Priebbenow and C. Bolm , “C–H Activation of Methyl Arenes in the MnO_2_‐Mediated Aroylation of N‐Chlorosulfoximines,” Organic Letters 16 (2014): 1650. 24588424 10.1021/ol5003016

[37] M. Muneeswara , S. S. Kotha , and G. Sekar , “Iron‐Catalyzed One‐Pot N‐Aroylation of NH‐Sulfoximines with Methylarenes through Benzylic C–H Bond Oxidation,” Synthesis 48 (2016): 1541.

[38] Z. Zhao , Y. Li , Y. Wu , X. Zhang , and J. Wang , “Metal‐Free Synthesis of Sulfoximines via Oxidative Imination of Sulfides,” RSC Advances 5 (2015): 75386.

[39] Y. Zou , Y. Chen , L. Liu , J. Sun , and Y. Wu , “Direct and Efficient Synthesis of Sulfoximines under Mild Conditions,” Chemical Communications 51 (2015): 14889.26302682

[40] S.‐L. Huang , J. R. Manning , and D. J. Cram , “Sulfoximines. Preparation and Rearrangement Reactions,” Journal of Organic Chemistry 44 (1979): 2510.

[41] C. Bolm , O. Simic , and G. Schlingloff , “Sulfoximines as Chiral Auxiliaries in Asymmetric Synthesis,” Synthesis 2002 (2002): 879.

[42] C. Bolm , G. Schlingloff , and O. Simic , “Sulfoximines in Organometallic Chemistry,” Journal of Organometallic Chemistry 687 (2003): 444.

[43] G. Y. Cho and C. Bolm , “Asymmetric Transformations Using Chiral Sulfoximines,” Organic Letters 7 (2005): 1351. 15787504 10.1021/ol050176b

[44] C. P. R. Hackenberger , K. Schwarzer , R. Seiwert , and R. Kunz , “Sulfoximines in Stereoselective Synthesis,” Chemistry ‐ A European Journal 10 (2004): 2942.15214076

[45] M. R. Yadav , R. K. Rit , M. Shankar , and A. K. Sahoo , “Recent Developments in Sulfoximine Chemistry,” Chemistry ‐ A European Journal 18 (2012): 5541.22461080

[46] T. Siu and C. Bolm , “Asymmetric Reactions Mediated by Chiral Sulfoximines,” Organic Letters 4 (2002): 1839.12027627

[47] L. Wang , J. Li , J. Wang , and Y. Zhang , “Catalytic Enantioselective Synthesis of Sulfoximines,” Advanced Synthesis & Catalysis 355 (2013): 1490.

[48] W.‐J. Qin , Y. Li , J. Sun , and X.‐Y. Shi , “Synthesis and Reactivity of Sulfoximine Derivatives,” Tetrahedron 71 (2015): 1182.

[49] Y. Wang , H. Yang , Y. Zheng , et al., “Carbon Monoxide Enabling Synergistic Carbonylation and (hetero)aryl Migration,” Nature Catalysis 7 (2024): 1065.

[50] R. M. B. Carrilho , M. J. F. Calvete , G. Mikle , L. Kollár , and M. M. Pereira , “Carbon Monoxide as a C1 Building Block in Fine Chemical Synthesis,” Chinese Journal of Chemistry 42 (2024): 199.

[51] S.‐R. Guo , P. S. Kumar , Y.‐Q. Yuan , and M.‐H. Yang , “Palladium Catalyzed Aroylation of NH‐Sulfoximines with Aryl Halides Using Chloroform as the CO Precursor,” Tetrahedron Letters 58 (2017): 2681.

[52] N. Sharma and G. Sekar , “Palladium Nanoparticles Catalyzed Aroylation of NH‐Sulfoximines with Aryl Iodides,” RSC Advances 6 (2016): 37226.

[53] B. D. Bala , N. Sharma , and G. Sekar , “Sulfoximinocarbonylation of Aryl Halides Using Heterogeneous Pd/C Catalyst,” RSC Advances 6 (2016): 97152.

[54] M. Afzal , S. Agarwal , R. H. Elshaikh , et al., “Carbon Monoxide Poisoning: Diagnosis, Prognostic Factors, Treatment Strategies, and Future Perspectives,” Diagnostics 15 (2025): 581. 40075828 10.3390/diagnostics15050581 PMC11899572

[55] A. Y. Abramov , I. Myers , and P. R. Angelova , “Carbon Monoxide: A Pleiotropic Redox Regulator of Life and Death,” Antioxidants 13 (2024): 1121. 39334780 10.3390/antiox13091121 PMC11428877

[56] C. A. Ohlin , P. J. Dyson , and G. Laurenczy , “Carbon Monoxide Solubility in Ionic Liquids: Determination, Prediction and Relevance to Hydroformylation,” Chemical Communications 9 (2004): 1070. 10.1039/b401537a 15116189

[57] A. Henni , P. Tontiwachwuthikul , and A. Chakma , “Solubilities of Carbon Dioxide in Polyethylene Glycol Ethers,” Canadian Journal of Chemical Engineering 83 (2005): 358.

[58] R. Koelliker and H. Thies , “Solubility of Carbon Monoxide in n‐Hexane Between 293 and 473 K and CO Pressures up to 200 bar,” Journal of Chemical and Engineering Data 38 (1993): 437.

[59] S. P. Tonner , M. S. Wainwright , D. L. Trimm , and N. W. Cant , “Solubility of Carbon Monoxide in Alcohols,” Journal of Chemical & Engineering Data 28 (1983): 59.

[60] S. E. Raby‐Buck , J. Devlin , P. Gupta , et al., “Continuous Flow Chemistry for Molecular Synthesis,” Nature Reviews Methods Primers 5 (2025): 44.

[61] A. I. Alfano , J. García‐Lacuna , O. M. Griffiths , S. V. Ley , and M. Baumann , “Continuous Flow Synthesis Enabling Reaction Discovery,” Chemical Science 15 (2024): 4618. 38550700 10.1039/d3sc06808k PMC10967013

[62] L. Capaldo , Z. Wen , and T. Noël , “A Field Guide to Flow Chemistry for Synthetic Organic Chemists,” Chemical Science 14 (2023): 4230. 37123197 10.1039/d3sc00992k PMC10132167

[63] C. A. Hone and C. O. Kappe , “Towards the Standardization of Flow Chemistry Protocols for Organic Reactions,” Chemistry – Methods 1 (2021): 454.

[64] M. Guidi , P. H. Seeberger , and K. Gilmore , “How to Approach Flow Chemistry,” Chemical Society Reviews 49 (2020): 8910. 33140749 10.1039/c9cs00832b

[65] M. B. Plutschack , B. Pieber , K. Gilmore , and P. H. Seeberger , “The Hitchhiker's Guide to Flow Chemistry,” Chemical Reviews 117 (2017): 11796. 28570059 10.1021/acs.chemrev.7b00183

[66] C. J. Mallia and I. R. Baxendale , “The Use of Gases in Flow Synthesis,” Organic Process Research and Development 20 (2016): 327.

[67] S. V. F. Hansen , Z. E. Wilson , T. Ulven , and S. V. Ley , “Controlled Generation and use of Carbon Monoxide in Flow,” Reaction Chemistry and Engineering 1 (2016): 280.

[68] K. Rao , A. Sharma , G. K. Rathod , A. S. Barahdia , and R. Jain , “Aminocarbonylation Using CO Surrogates,” Organic and Biomolecular Chemistry 23 (2025): 980. 39666374 10.1039/d4ob01639d

[69] L. A. A. Aronica , “Carbon Monoxide Surrogates for Palladium‐Catalyzed Suzuki‐Miyaura and Sonogashira Carbonylative Cross‐Coupling Reactions,” Inorganica Chimica Acta 558 (2023): 121763.

[70] M. V. Khedkar , S. R. Khan , T. L. Lambat , R. G. Chaudhary , and A. A. Abdala , “CO Surrogates: A Green Alternative in Palladium‐Catalyzed CO Gas Free Carbonylation Reactions,” Current Organic Chemistry 24 (2020): 2588.

[71] A. Ładosz , A. Friedli , A. Lhuillery , and G. Rueedi , “Carbonylations in Flow: Tube‐in‐Tube Reactor vs. Gas–Liquid Slug Flow,” Reaction Chemistry and Engineering 9 (2024): 3172.

[72] M. Brzozowski , M. O’Brien , S. V. Ley , and A. Polyzos , “Flow Chemistry: Intelligent Processing of Gas–Liquid Transformations Using a Tube‐in‐Tube Reactor,” Accounts of Chemical Research 48 (2015): 349. 25611216 10.1021/ar500359m

[73] E. R. Murphy , J. R. Martinelli , N. Zaborenko , S. L. Buchwald , and K. F. Jensen , “Accelerating Reactions with Microreactors at Elevated Temperatures and Pressures: Profiling Aminocarbonylation Reactions,” Angewandte Chemie International Edition 46 (2007): 1734. 17397088 10.1002/anie.200604175

[74] C. B. Kelly , X. Lee , M. A. Mercadante , and N. E. Leadbeater , “A Continuous‐Flow Approach to Palladium‐Catalyzed Alkoxycarbonylation Reactions,” Organic Process Research & Development 15 (2011): 717.

[75] P. W. Miller , N. J. Long , A. J. de Mello , R. Vilar , J. Passchier , and A. Gee , “Rapid Formation of Amides via Carbonylative Coupling Reactions Using a Microfluidic Device,” Chemical Communications (2006): 546–548. 16432578 10.1039/b515710b

[76] C. Brancour , T. Fukuyama , Y. Mukai , T. Skrydstrup , and I. Ryu , “Modernized Low Pressure Carbonylation Methods in Batch and Flow Employing Common Acids as a CO Source,” Organic Letters 15 (2013): 2794. 23697868 10.1021/ol401092a

[77] X. Gong , P. W. Miller , A. D. Gee , N. J. Long , A. J. de Mello , and R. Vilar , “Gas–Liquid Segmented Flow Microfluidics for Screening Pd‐Catalyzed Carbonylation Reactions,” Chemistry – A European Journal 18 (2012): 2768. 22331821 10.1002/chem.201104059

[78] Q. Liu , L. Wu , R. Jackstell , and M. Beller , “Using Carbon Dioxide as a Building Block in Organic Synthesis,” Nature Communications 6 (2015): 5933. 10.1038/ncomms6933 25600683

[79] S. Sengupta , A. Jha , P. Shende , R. Maskara , and A. K. Das , “Catalytic Performance of Co and Ni Doped Fe‐Based Catalysts for the Hydrogenation of CO_2_ to CO via Reverse Water‐Gas Shift Reaction,” Journal of Environmental Chemical Engineering 7 (2019): 102911.

[80] R. M. Bown , M. Joyce , Q. Zhang , T. R. Reina , and M. S. Duyar , “Identifying Commercial Opportunities for the Reverse Water Gas Shift Reaction,” Energy Technology 9 (2021): 2100554.

[81] R. Sang , Y. Hu , R. Razzaq , et al., “A Practical Concept for Catalytic Carbonylations Using Carbon Dioxide,” Nature Communications 13 (2022): 4432. 10.1038/s41467-022-32030-8 PMC9338997 35908063

[82] X. He , Y. Cao , X.‐D. Lang , N. Wang , and L.‐N. He , “Integrative Photoreduction of CO_2_ with Subsequent Carbonylation: Photocatalysis for Reductive Functionalization of CO_2_ ,” ChemSusChem 11 (2018): 3382–3387. 30102840 10.1002/cssc.201801621

[83] J. Zhang , X. Kang , Y. Yan , X. Ding , L. He , and Y. Li , “Cascade Electrocatalytic and Thermocatalytic Reduction of CO_2_ to Propionaldehyde,” Angewandte Chemie International Edition 63 (2024): e202315777. 38233351 10.1002/anie.202315777

[84] H. Shi , S. Jia , M. Dong , H. Wu , Z. Huang , and K. Dong , “Coupling CO_2_ Electroreduction to CO with Alkyne Alkoxycarbonylation,” Organic Letters 26 (2024): 8982. 39412186 10.1021/acs.orglett.4c02431

[85] H. M. Dodge , B. S. Natinsky , B. J. Jolly , et al., “Polyketones from Carbon Dioxide and Ethylene by Integrating Electrochemical and Organometallic Catalysis,” ACS Catalysis 13 (2023): 4053.

[86] R. Miró , E. Fernández‐Llamazares , C. Godard , M. D. de los Bernardos , and A. Gual , “Synergism between Iron Porphyrin and Dicationic Ionic Liquids: Tandem CO_2_ Electroreduction–Carbonylation Reactions,” Chemical Communications 58 (2022): 10552. 36047332 10.1039/d2cc03641j

[87] L. Ponsard , E. Nicolas , N. H. Tran , et al., “Coupling Electrocatalytic CO_2_ Reduction with Thermocatalysis Enables the Formation of a Lactone Monomer,” ChemSusChem 14 (2021): 2198. 33687795 10.1002/cssc.202100459

[88] M. T. Jensen , M. H. Rønne , A. K. Ravn , et al., “Scalable Carbon Dioxide Electroreduction Coupled to Carbonylation Chemistry,” Nature Communications 8 (2017): 489. 10.1038/s41467-017-00559-8 PMC5591205 28887452

[89] M. Gaudeau , M. Zhang , M. Tatoulian , C. Lescot , and S. Ognier , “Fast Carbonylation Reaction from CO_2_ Using Plasma Gas/Liquid Microreactors for Radiolabeling Applications,” Reaction Chemistry & Engineering 5 (2020): 1981–1991.

[90] K. Stagel , K. Rath , P. M. Kathe , et al., “Online Coupling High‐Temperature Electrolysis with Carbonylation Reactions: A Powerful Method for Continuous Carbon Dioxide Utilization,” Angewandte Chemie International Edition 64 (2025): e202420578. 40190051 10.1002/anie.202420578 PMC12105702

[91] C. Li , Y. Yang , X. Zheng , C. Zhang , H. Cai , and W. Lin , “Photocatalyzed Sulfoximination/Amidation of (Het)arylethenes Tethered N‐Tosyl Amide: A Versatile Entry to Sulfoximidoyl β‐ and γ‐Lactams,” Organic Chemistry Frontiers 11 (2024): 4508.

[92] K. R. Thete , V. Vara , A. A. Khan , and M. Muthukrishnan , “Phosphine‐Catalyzed Nucleophilic Ring Opening of Cyclopropenones with Sulfoximines to Access N‐α,β‐Unsaturated Acyl Sulfoximines,” Journal of Organic Chemistry 90 (2025): 10643. 40690324 10.1021/acs.joc.5c00883

[93] T. Alam , S. Gupta , and B. K. Patel , “Electrochemical NH‐Sulfoximidation with α‐Keto Acids,” ChemPhysChem 25 (2024): e202400599. 38884606 10.1002/cphc.202400599

[94] N. Chakraborty , K. K. Rajbongshi , A. Dahiya , B. Das , A. Vaishnani , and B. K. Patel , “PIDA/I_2_‐Mediated Photo‐Induced Aerobic N‐Acylation of Sulfoximines with Methylarenes,” Organic & Biomolecular Chemistry 22 (2024): 2375. 38436055 10.1039/d4ob00175c

[95] A. Nenning , M. Holzmann , J. Fleig , and A. K. Opitz , “Excellent Kinetics of Single‐Phase Gd‐Doped Ceria Fuel Electrodes in Solid Oxide Cells,” Materials Advances 2 (2021): 5422.

[96] F. M. S. Rodrigues , L. D. Dias , M. J. F. Calvete , et al., “Immobilization of Rh(I)‐N‐Xantphos and Fe(II)‐C‐Scorpionate onto Magnetic Nanoparticles: Reusable Catalytic System for Sequential Hydroformylation/Acetalization,” Catalysts 11 (2021): 608.

[97] N. A. Stini , P. L. Gkizis , and C. G. Kokotos , “Cyrene: A Bio‐Based Novel and Sustainable Solvent for Organic Synthesis,” Green Chemistry 24 (2022): 6435.

[98] A. Citarella , A. Amenta , D. Passarella , and N. Micale , “Cyrene: A Green Solvent for the Synthesis of Bioactive Molecules and Functional Biomaterials,” International Journal of Molecular Sciences 23 (2022): 15960. 36555601 10.3390/ijms232415960 PMC9783252

