# Supplementary material for: Closing the Carbon Loop: Continuous‐Flow Synthesis of N‐Aroyl Sulfoximines via Electrochemical CO2 Reduction
Source: ChemSusChem. 2026 Jun 27;19(13):e70810. doi: 10.1002/cssc.70810 (PMC13309917; doi:10.1002/cssc.70810)
Supplement: Supplementary file 1 — Supplementary Material [file CSSC-19-e70810-s001.pdf]

Supporting Information

Closing the Carbon Loop: Continuous Flow Synthesis of *N*-Aroyl Sulfoximines via Electrochemical CO_2 Reduction

Giada Moroni[a], Maximilian Weiss[b], Giulia Russo[a,c], Gloria Biscontini[a], Olga Lanaridi[b], Andreas Limbeck[b], Tobias Huber[b], Alexander Karl Opitz[b], Katharina Bica-Schröder[a]*

[a] Institute of Applied Synthetic Chemistry, TU Wien, Getreidemarkt 9/163, Vienna 1060, Austria

[b] Institute of Chemical Technologies and Analytics, TU Wien, Getreidemarkt 9/164, Vienna 1060, Austria

[c] Department of Chemistry, Life Sciences and Environmental Sustainability, University of Parma, Parco Area delle Scienze 17/A, 43122, Parma, Italy

Table of contents

1. General remarks **1**

2. Experimental procedures **2**

2.1. General procedure for the preparation of sulfoximines **2**

2.2. General procedure for aroylation of *N*H-sulfoximines under batch conditions **2**

2.3. General procedure for aroylation of *N*H-sulfoximines under flow conditions **3**

2.4. Large scale preparation of aroylated *N*H-sulfoximines model compound (**6a**) under flow conditions **6**

3. Solid Oxide Electrolysis Cell (SOEC) set-up **6**

3.1. Experiments for aroylation of *N*H-sulfoximines under flow conditions by using CO/CO_2 gas mixture on model compound (**6a**) **8**

4. Synthesis and characterization of heterogeneous Pd-based catalyst (**7**) **9**

4.1. Synthetic procedures for the preparation of heterogeneous Pd-based catalyst (**7**) **9**

4.2. Characterization of heterogeneous Pd-based catalyst (**7**) and and its stability after flow reactions **9**

4.3. General continuous flow procedure for aroylation of *N*H-sulfoximines by using heterogeneous Pd-based catalyst (**7**) **10**

5. Screening of Cyrene™ as solvent for *N*-aroylation of sulfoximines under flow conditions **11**

6. Copies of NMR spectra **12**

7. References **37**

1. General Remarks

Unless otherwise noted, all purchased chemicals from commercial suppliers were used without further purification. Cyrene™ was purchased from Sigma Aldrich.Column chromatography was performed on standard glass columns using Merck (40-60 µm) silica gel with pre-distilled solvents. For TLC analysis, pre-coated aluminum-backed plates were used (Merck, silica gel 60 F254). All compounds were detected at 254 nm. ^{1}H-, ^{13}C-, and ^{19}F spectra were recorded from $CDCl_3$ solutions on a Bruker Avance UltraShield 400 MHz (^{1}H: 400 MHz, ^{13}C: 101 MHz, ^{19}F: 376 MHz) NMR instrument. Chemical shifts are reported in parts per million (ppm) and were calibrated to the residual solvent signal (e.g., $CDCl_3$, ^{1}H: 7.26 ppm, ^{13}C: 77.0 ppm). Coupling constants are reported in hertz (Hz). The assignments are based on the comparison with reported spectra. High-resolution mass spectrometry (HRMS) was carried out using an Agilent 1100/1200 HPLC with a 6230 AJS ESI-TOF MS. Infrared (IR) spectra were recorded with the aid of a Perkin-Elmer Spectrum65 FT IS spectrometer with absorption maxima ($ν_{max}$) quoted in wavenumbers (cm^{-1}). Continuous flow experiments were performed with the aid of a Vapourtec® E-Series flow chemistry device using a standard 10 mL coil reactor. Mass flow controllers were purchased from Brooks Instruments; SLA5850 series with four channel secondary electronics (Model 0254). The MFCs were purchased with a configuration that allowed a maximum inlet pressure of 7.5 atm and an outlet pressure of 1 atm. Carbon monoxide and carbon dioxide were provided from the respective cylinders equipped with a low-pressure regulator, outputting 5 bar (2 bar when the electrochemical cell was employed). For the continuous-flow experiments, the gases were introduced using a V-3 pump after passing through the gas mixer

N.B. Carbon monoxide (CO) is a highly toxic, colorless, and odorless gas. All experiments involving CO, including batch and continuous flow reactions, were performed in a well-ventilated fume hood using appropriate gas-handling and pressure-rated equipment with leak-free connections. Continuous monitoring and standard laboratory safety protocols for toxic gases were strictly followed.

2. Experimental procedures:

2.1. General procedure for the preparation of sulfoximines [78]:

Sulfide compounds (thioanisol, *p*-tolyl methyl sulfide, 4-methoxythioanisole, and ethyl phenyl sulfide) (1 eq.), (diacetoxyiodo) benzene (2.1 eq.), and ammonium bicarbonate (3.0 eq.) were added to a 250 mL flask containing a stirring bar. MeOH (0.4 M) was added, and the reaction was stirred at 25 °C for 3 hours. After the completion of the reaction, the solvent was removed under reduced pressure. The reaction crude was filtered through a Hirsh and washed with CH_2Cl_2 to remove inorganic salts. The reaction crude was purified by flash chromatography on silica gel by using CH_2Cl_2/EtOAc (from 100:0 to 70:30) as eluent solvent system obtaining the desired products (**1-5**).

imino(methyl)(phenyl)-λ^6-sulfanone (1). The spectroscopic data are in agreement with those reported in the literature [6]. Yield: >99%. 6.67 g, 43.0000 mmol; light yellow oil. Rf: 0.27 (CH_2Cl_2/EtOAc 75:25). ^{1}H-NMR (400 MHz, $CDCl_3$): δ= 8.04 – 7.99 (m, 2H), 7.65 – 7.59 (m, 1H), 7.59 – 7.52 (m, 2H), 3.11 (s, 3H), 2.49 (s, 1H). ^{13}C-NMR (101 MHz, $CDCl_3$): δ= 143.7, 133.2, 129.4, 127.8, 46.3.

imino(methyl)(*p*-tolyl)-λ^6-sulfanone (2). The spectroscopic data are in agreement with those reported in the literature [6]. Yield: 90%. 1.10 g, 6.4993 mmol; colorless oil or white solid after drying. Rf: 0.42 (CH_2Cl_2/MeOH 95:5). ^{1}H-NMR (400 MHz, $CDCl_3$): δ = 7.89 (dd, J = 8.3, 1.6 Hz, 2H), 7.39 – 7.32 (m, 2H), 3.18 – 3.08 (s, 3H), 2.44 (s, 3H). ^{13}C-NMR (101.6 MHz, $CDCl_3$): δ= 144.3, 140.1, 130.1, 127.9, 46.3, 21.5.

imino(4-methoxyphenyl)(methyl)-λ^6-sulfanone (3). The spectroscopic data are in agreement with those reported in the literature [86]. Yield: 98%. 1.18 g, 6.3541 mmol; colorless oil or white solid after drying. Rf: 0.35 (CH_2Cl_2/MeOH 95:5). ^{1}H-NMR (400 MHz, $CDCl_3$): δ= 7.94 – 7.84 (m, 2H), 6.98 – 6.95 (m, 2H), 3.84 (s, 3H), 3.52 (brs, 1H), 3.06 (s, 3H). ^{13}C-NMR (101.6 MHz, $CDCl_3$): δ= 163.4, 135.0, 129.9, 114.5, 55.8, 46.6.

ethyl(imino)(phenyl)-λ^6-sulfanone (4): The spectroscopic data are in agreement with those reported in the literature [87]. Yield: 97%. 1.19 g, 7.0310 mmol; pale-yellow oil. Rf: 0.44 (CH_2Cl_2/MeOH 95:5). ^{1}H-NMR (400 MHz, $CDCl_3$): δ= 8.00 – 7.93 (m, 2H), 7.65 – 7.58 (m, 1H), 7.58 – 7.51 (m, 2H), 3.17 (q, J = 7.3 Hz, 2H), 1.25 (td, J = 7.4, 0.7 Hz, 3H). ^{13}C-NMR (101 MHz, CDCl3): δ= 141.5, 133.2, 129.3, 128.7, 51.9, 8.0.

(4-chlorophenyl)(imino)(methyl)-λ^6-sulfanone (5): The spectroscopic data are in agreement with those reported in the literature [86]. Yield: 98%. 1.17 g, 6.1686 mmol; pale-yellow oil. Rf: 0.50 (CH_2Cl_2/MeOH 95:5). ^{1}H-NMR (400 MHz, $CDCl_3$): δ= 7.97 – 7.92 (m, 2H), 7.55 – 7.49 (m, 2H), 3.10 (s, 3H). ^{13}C-NMR (101 MHz, $CDCl_3$): δ= 142.2, 139.9, 129.7, 129.4, 46.3.

2.2. General procedure for aroylation of *NH*-sulfoximines under batch conditions [39]:

In a vial with a septum cap equipped with a magnetic stir bar, 4-iodotoluene (109 mg, 0.5 mmol, 1 eq.), (methylsulfonimidoyl)benzene (**1**, 116 mg, 0.75 mmol, 1.5 eq.), palladium catalyst (Pd/C 10 % *w/w* or $Pd(OAc)_2$; 0.1 mmol, 0.02 eq.), *N*-xantphos (55 mg, 0.1 mmol, 0.2 eq.), and base (K_2CO_3, DIPEA, pyridine, DBU, Et_3N, TBAF, pyrrolidine, DMAP, morpholine, piperidine, DABCO; 0.5 mmol, 1 eq.) were dissolved in DMF (1 mL, 0.5 M). CO balloon (atmospheric pressure) was introduced, and the reaction mixture was stirred at 80 °C for 12 h. After that, the reaction mixture was allowed to cool down to 25 °C. The reaction crude was basified with NaOH (0.1 M) to remove unreacted sulfoximine in excess and extracted with CH_2Cl_2 (x3). The combined organic layers were washed with an aqueous solution of LiCl 20% *w/v*, brine, dried over anhydrous Na_2SO_4, and concentrated under reduced pressure. The reaction crude was characterized by ^{1}H-NMR (1 scan) and the conversion was determined by comparing the methyl of tolyl group (3H, s) of **6a** (2.40 ppm) with the methyl group of 4-iodotoluene (2.29 ppm) of the reaction mixture or aromatic protons in *α*-positions of tolyl groups (2H, m) (7.23-7.18 for **6a** and 6.94-6.90 ppm for 4-iodotoluene, respectively).

2.3. General procedure for aroylation of *NH*-sulfoximines under flow conditions:

A solution of aryl iodide (0.5 mmol, 1 eq.), sulfoximine (0.75 mmol, 1.5 eq.), $Pd(OAc)_2$ (2.2 mg, 0.01 mmol, 0.02 eq.), *N*-xantphos (55 mg, 0.1 mmol, 0.2 eq.), and 1,4-diazabicyclo (2.2.2)octan (DABCO, 56 mg, 0.5 mmol, 1 eq.) dissolved in DMF (1 mL, 0.5 M) was pumped at 100 µL min^{-1} and mixed with a streaming of CO at 1.00 mL min^{-1} controlled by a MFC (1.25 mL min^{-1}). The reaction mixture was allowed to react inside a 10 mL coil reactor for 40 minutes of residence time at 80 °C and 6 bar (BPR). After the death volume, the outcome was collected for further 40 minutes. The crude was basified with NaOH (0.1 M) to remove unreacted sulfoximine in excess and extracted with CH_2Cl_2 (x3) (with exception of **6m**, **6n**, and **6q** an aqueous work-up was performed). The combined organic layers were washed with an aqueous solution of LiCl 20% *w/v*, brine, dried over anhydrous Na_2SO_4, and concentrated under reduced pressure. The reaction mixture was purified by flash chromatography on silica gel affording the *N*-aroylated sulfoximines (**6a-s**).

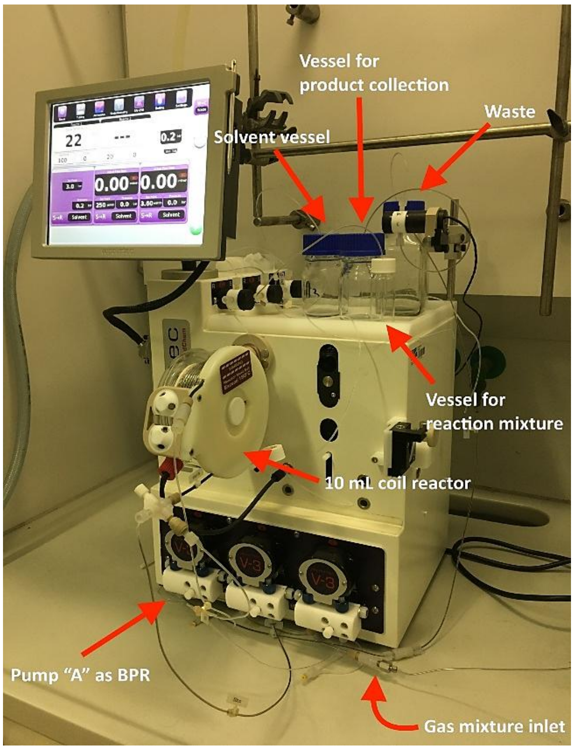

Figure S1: Continuous flow apparatus (Vapourtec ® E-Series easy-MedChem) employed for the sulfoximinocarbonylation.

4-methyl-*N*-(methyl(oxo)(phenyl)-λ⁶-sulfaneylidene)benzamide (6a): The spectroscopic data are in agreement with those reported in the literature [38-40]. Conv.: > 99%. Yield: 99%. 135 mg, 0.4938 mmol; white solid. Rf: 0.24 (*n*-hexane/EtOAc 7:3). ^{1}H-NMR (400 MHz, $CDCl_3$): δ= 8.09 – 8.01 (m, 4H), 7.71 – 7.64 (m, 1H), 7.64 – 7.57 (m, 2H), 7.24 – 7.18 (m, 2H), 3.46 (s, 3H), 2.40 (s, 3H). ^{13}C-NMR (101.6 MHz, $CDCl_3$): δ= 174.4, 142.8, 139.3, 133.8, 133.1, 129.8, 129.6, 128.9, 127.3, 44.5, 21.7.

***N*-(methyl(oxo)(phenyl)-λ⁶-sulfaneylidene)benzamide (6b).** The spectroscopic data are in agreement with those reported in the literature [38-40]. Yield: > 99%. 132 mg, 0.5090 mmol; pale-pink solid. Rf: 0.20 (*n*-hexane/EtOAc 7:3). ^{1}H-NMR (400 MHz, $CDCl_3$): δ= 8.21 – 8.14 (m, 2H), 8.09 – 8.03 (m, 2H), 7.72 – 7.57 (m, 3H), 7.54 – 7.48 (m, 1H), 7.45 – 7.38 (m, 2H), 3.46 (s, 3H). ^{13}C-NMR (101.6 MHz, $CDCl_3$): δ= 174.3, 139.1, 135.7, 133.9, 132.3, 129.8, 129.5, 128.1, 127.3, 44.5.

4-methoxy-*N*-(methyl(oxo)(phenyl)-λ⁶-sulfaneylidene)benzamide (6c): The spectroscopic data agree with those reported in the literature [39]. Yield: 86%. 124 mg, 0.4285 mmol; brownish solid. Rf: 0.09 (*n*-hexane/EtOAc 7:3). ^{1}H-NMR (400 MHz, $CDCl_3$): δ= 8.17 – 8.10 (m, 2H), 8.08 – 8.02 (m, 2H), 7.72 – 7.57 (m, 3H), 6.94 – 6.87 (m, 2H), 3.85 (d, J = 0.7 Hz, 3H), 3.45 (d, J = 0.8 Hz, 3H). ^{13}C-NMR (101.6 MHz, $CDCl_3$): δ= 173.9, 163.0, 139.4, 133.8, 131.6, 129.8, 128.4, 127.3, 113.3, 55.5, 44.5.

4-methyl-*N*-(methyl(oxo)(*p*-tolyl)-λ^6-sulfaneylidene)benzamide (6d): The spectroscopic data agree with those reported in the literature [39]. Conv.: > 99%. Yield: 83%. 119 mg, 0.4141 mmol; brownish solid. Rf: 0.22 (*n*-hexane/EtOAc 7:3). ^{1}H-NMR (400 MHz, $CDCl_3$): δ= 8.09 – 8.03 (m, 2H), 7.95 – 7.88 (m, 2H), 7.40 – 7.35 (m, 2H), 7.23 – 7.16 (m, 2H), 3.43 (s, 3H), 2.45 (s, 3H), 2.39 (s, 3H). ^{13}C-NMR (101.6 MHz, $CDCl_3$): δ= 174.3, 144.9, 142.7, 136.2, 133.2, 130.4, 129.6, 128.9, 127.3, 44.6, 21.7.

***N*-((4-methoxyphenyl)(methyl)(oxo)-λ^6-sulfaneylidene)-4-methylbenzamide (6e):** The spectroscopic data agree with those reported in the literature [38-40]. Conv.: > 99%. Yield: 86%. 131 mg, 0.4318 mmol; white solid. Rf: 0.12 (*n*-hexane/EtOAc 7:3). ^{1}H-NMR (400 MHz, $CDCl_3$): δ= 8.09 – 8.03 (m, 2H), 8.00 – 7.93 (m, 2H), 7.23 – 7.17 (m, 2H), 7.08 – 7.02 (m, 2H), 3.87 (s, 3H), 3.44 (s, 3H), 2.39 (s, 3H). ^{13}C-NMR (101.6 MHz, $CDCl_3$): δ= 174.3, 163.9, 142.7, 133.2, 130.3, 129.6, 129.5, 128.8, 115.0, 55.9, 44.8, 21.7.

***N*-(ethyl(oxo)(phenyl)-λ^6-sulfaneylidene)-4-methylbenzamide (6f):** The spectroscopic data agree with those reported in the literature [39-40]. Conv.: > 99%. Yield: 87%. 125 mg, 0.4350 mmol; pinkish solid. Rf: 0.20 (*n*-hexane/EtOAc 7:3). ^{1}H-NMR (400 MHz, $CDCl_3$): δ= 8.10 – 8.04 (m, 2H), 8.02 – 7.96 (m, 2H), 7.70 – 7.63 (m, 1H), 7.59 (ddt, J = 8.2, 6.8, 1.4 Hz, 2H), 7.24 – 7.17 (m, 2H), 3.60 (q, J = 7.4 Hz, 2H), 2.40 (s, 3H), 1.30 (t, J = 7.4 Hz, 3H). ^{13}C-NMR (101.6 MHz, $CDCl_3$): δ= 174.3, 142.7, 136.8, 133.8, 133.2, 129.7, 129.6, 128.8, 128.1, 50.7, 21.7, 7.4.

4-chloro-*N*-(methyl(oxo)(phenyl)-λ^6-sulfaneylidene)benzamide (6g): The spectroscopic data agree with those reported in the literature [39-40]. Yield: 98%. 144 mg, 0.4902 mmol; white solid. Rf: 0.20 (*n*-hexane/EtOAc 7:3). ^{1}H-NMR (400 MHz, $CDCl_3$): δ= 8.13 – 8.07 (m, 2H), 8.07 – 8.01 (m, 2H), 7.73 – 7.58 (m, 3H), 7.40 – 7.34 (m, 2H), 3.46 (s, 3H). ^{13}C-NMR (101.6 MHz, $CDCl_3$): δ= 173.3, 138.9, 138.5, 134.2, 134.0, 131.0, 129.8, 128.4, 127.2, 44.5.

***N*-((4-chlorophenyl)(methyl)(oxo)-λ^6-sulfaneylidene)-4-methylbenzamide (6h):** The spectroscopic data agree with those reported in the literature [38-40]. Conv.: > 99%. Yield: 79%. 121 mg, 0.3931 mmol; white solid. Rf: 0.27 (*n*-hexane/EtOAc 7:3). ^{1}H-NMR (400 MHz, $CDCl_3$): δ= 8.07 – 8.00 (m, 2H), 8.00 – 7.93 (m, 2H), 7.60 – 7.52 (m, 2H), 7.23 – 7.16 (m, 2H), 3.43 (s, 3H), 2.39 (s, 3H). ^{13}C-NMR (101.6 MHz, $CDCl_3$): δ= 174.2, 143.0, 140.6, 137.7, 132.7, 130.1, 129.6, 128.9, 128.8, 44.5, 21.7.

***N*-(methyl(oxo)(phenyl)-λ^6-sulfaneylidene)-4-(trifluoromethyl)benzamide (6i):** The spectroscopic data agree with those reported in the literature [38-39]. Yield: 73%. 120 mg, 0.3666 mmol; brownish solid. Rf: 0.36 (*n*-hexane/EtOAc 6:4). ^{1}H-NMR (400 MHz, $CDCl_3$): δ= 8.31 – 8.21 (m, 2H), 8.06 – 7.99 (m, 2H), 7.73 – 7.55 (m, 5H), 3.46 (s, 3H). ^{19}F-NMR (374 MHz, $CDCl_3$): -62.8. ^{13}C-NMR (101.6 MHz, $CDCl_3$): δ= 172.9, 138.8, 138.6, 134.1, 133.5 (q, J = 32.2), 129.8 (x2), 127.1, 125.1 (q, J = 4.0), 124.0 (q, J = 272.6), 44.4.

4-fluoro-*N*-(methyl(oxo)(phenyl)-λ^6-sulfaneylidene)benzamide (6j): The spectroscopic data agree with those reported in the literature [39]. Yield: 86%. 120 mg, 0.4327 mmol; pinkish solid. Rf: 0.20 (*n*-hexane/EtOAc 7:3). ^{1}H-NMR (400 MHz, $CDCl_3$): δ= 8.21 – 8.14 (m, 2H), 8.08 – 8.02 (m, 2H), 7.73 – 7.66 (m, 1H), 7.66 – 7.59 (m, 2H), 7.11 – 7.03 (m, 2H), 3.47 (s, 3H). ^{19}F-NMR (374 MHz, $CDCl_3$): -107.6. ^{13}C-NMR (101.6 MHz, $CDCl_3$): δ= 173.3, 165.6, (d, J = 253.,5 Hz) 139.1, 134.0, 132.0 (d, J = 9.1 Hz), 129.9, 127.3, 115.1 (d, J = 21.1 Hz), 44.5.

***N*-(methyl(oxo)(phenyl)-λ^6-sulfaneylidene)thiophene-2-carboxamide (6k):** The spectroscopic data agree with those reported in the literature [40]. Yield: 77%. 102 mg, 0.3850 mmol; brownish solid. Rf: 0.23 (*n*-hexane/EtOAc 7:3). ^{1}H-NMR (400 MHz, $CDCl_3$): δ= 8.07 – 8.02 (m, 2H), 7.79 (dd, J = 3.7, 1.3 Hz, 1H), 7.71 – 7.65 (m, 1H), 7.61 (ddt, J = 8.3, 6.8, 1.4 Hz, 2H), 7.47 (dd, J = 5.0, 1.3 Hz, 1H), 7.06 (dd, J = 5.0, 3.7 Hz, 1H), 3.45 (s, 3H). ^{13}C-NMR (101.6 MHz, $CDCl_3$): δ= 169.0, 141.3, 138.9, 134.0, 132.2, 131.7, 129.8, 127.8, 127.3, 44.5.

***N*-(methyl(oxo)(phenyl)-λ^6-sulfaneylidene)-2-naphthamide (6l):** The spectroscopic data agree with those reported in the literature [39-40]. Yield: 54%. 84 mg, 0.2715 mmol; dense dark red oil. Rf: 0.30 and 0.16 (*n*-hexane/EtOAc 7:3 and *n*-hexane/acetone 8:2, respectively). ^{1}H-NMR (400 MHz, $CDCl_3$): δ= 9.01 (dt, J = 8.6, 1.0 Hz, 1H), 8.35 (dd, J = 7.2, 1.3 Hz, 1H), 8.14 – 8.07 (m, 2H), 7.97 (dt, J = 8.2, 1.1 Hz, 1H), 7.88 – 7.83 (m,

1H), 7.73 – 7.68 (m, 1H), 7.67 – 7.60 (m, 2H), 7.54 (ddd, J = 8.6, 6.8, 1.6 Hz, 1H), 7.51 – 7.45 (m, 2H), 3.50 (s, 3H). ^{13}C-NMR (101.6 MHz, $CDCl_3$): δ= 176.7, 139.2, 134.1, 133.9, 133.0, 132.5, 131.5, 130.0, 129.9, 128.4, 127.3 (x2), 126.6, 126.0, 124.6, 44.7.

***N*-(methyl(oxo)(phenyl)-λ^6-sulfaneylidene)nicotinamide (6m):** Yield: 71%. 93 mg, 0.3572 mmol; pale-yellow solid; mp: 82.1–83.9 °C; Rf: 0.29 (DCM/EtOAc 7:3 + 1% Et_3N). ^{1}H-NMR (400 MHz, $CDCl_3$): δ= 9.34 (dd, *J* = 2.2, 0.9 Hz, 1H), 8.70 (dd, *J* = 4.8, 1.8 Hz, 1H), 8.36 (dt, *J* = 7.9, 2.0 Hz, 1H), 8.07 – 8.00 (m, 2H), 7.73 – 7.65 (m, 1H), 7.61 (ddt, *J* = 8.3, 6.8, 1.3 Hz, 2H), 7.33 (ddd, *J* = 7.9, 4.8, 0.9 Hz, 1H), 3.47 (s, 3H). ^{13}C-NMR (101.6 MHz, $CDCl_3$): δ= 172.7, 152.7, 151.1, 138.6, 136.8, 134.1, 131.2, 129.9, 127.2, 123.1, 44.5. IR ATR (ν_{max}/cm^{-1}): 3020, 2924, 1982, 1626, 1584, 1476, 1448, 1412, 1326, 1288, 1218, 1190, 1140, 1092, 1024, 988, 972, 926, 836, 778, 742, 702, 686, 620, 524, 500. HRMS (*m/z*): [MH]$^+$ calcd for $C_{14}H_{12}N_2O_2S$: 261.0692; found: 261.0698.

5-chloro-*N*-(methyl(oxo)(phenyl)-λ^6-sulfaneylidene)nicotinamide (6n): Yield: 50%. 74 mg, 0.2510 mmol; yellow solid; mp: 120.2–121.0 °C; Rf: 0.48 and 0.68 (DCM/EtOAc 7:3 + 1% Et_3N and DCM/acetone 8:2 + 1% Et_3N, respectively). ^{1}H-NMR (400 MHz, $CDCl_3$): δ= 9.21 (d, J = 1.8 Hz, 1H), 8.68 (d, J = 2.4 Hz, 1H), 8.36 (dd, J = 2.5, 1.8 Hz, 1H), 8.08 – 8.02 (m, 2H), 7.73 (ddt, J = 8.3, 6.6, 1.3 Hz, 1H), 7.65 (ddt, J = 8.3, 6.8, 1.3 Hz, 2H), 3.49 (s, 3H). ^{13}C-NMR (101.6 MHz, $CDCl_3$): δ= 171.5, 151.6, 148.8, 138.4, 136.6, 134.4, 132.3, 132.0, 130.0, 127.2, 44.6. IR ATR (ν_{max}/cm^{-1}): 3066, 3022, 2928, 2166, 2116, 1622, 1580, 1562, 1478, 1438, 1412, 1322, 1300, 1280, 1208, 1180, 1136, 1092, 1026, 1004, 978, 958, 898, 830, 796, 756, 738, 684. HRMS (*m/z*): [MNa]$^+$ calcd for $C_{13}H_{11}ClN_2O_2SNa$: 317.0122; found: 317.0123.

4-cyano-*N*-(methyl(oxo)(phenyl)-λ^6-sulfaneylidene)benzamide (6o): The spectroscopic data agree with those reported in the literature [38-40]. Yield: 65%. 93 mg, 0.3271 mmol; yellow solid. Rf: 0.27 and 0.31 (*n*-hexane/EtOAc 6:4 and *n*-hexane/acetone 6:4). ^{1}H-NMR (400 MHz, $CDCl_3$): δ= 8.28 – 8.23 (m, 2H), 8.07 – 8.02 (m, 2H), 7.75 – 7.69 (m, 3H), 7.67 – 7.61 (m, 2H), 3.49 (s, 3H). ^{13}C-NMR (101.6 MHz, $CDCl_3$): δ= 172.5, 139.6, 138.6, 134.3, 132.1, 130.0 (x2), 127.2, 118.6, 115.5, 44.5.

***N*-(methyl(oxo)(phenyl)-λ^6-sulfaneylidene)-4-nitrobenzamide (6p):** The spectroscopic data agree with those reported in the literature [39-40]. Yield: 79%. 121 mg, 0.3976 mmol; yellowish solid. Rf: 0.17 and 0.54 (*n*-hexane/EtOAc 7:3 and *n*-hexane/acetone 6:4). ^{1}H-NMR (400 MHz, $CDCl_3$): δ= 8.35 – 8.29 (m, 2H), 8.28 – 8.23 (m, 2H), 8.08 – 8.03 (m, 2H), 7.76 – 7.70 (m, 1H), 7.69 – 7.62 (m, 2H), 3.50 (s, 3H). ^{13}C-NMR (101.6 MHz, $CDCl_3$): δ= 172.1, 150.1, 141.2, 138.4, 134.3, 130.5, 129.9, 127.2, 123.3, 44.4.

4-((methyl(oxo)(phenyl)-λ^6-sulfaneylidene)carbamoyl)phenyl acetate (6q): Conv.: > 99%. Yield: 66%. 105 mg, 0.3308 mmol; white solid; mp: 136.5–138.5 °C; Rf: 0.15 (*n*-hexane/EtOAc 65:35). ^{1}H-NMR (400 MHz, $CDCl_3$): δ= 8.22 – 8.17 (m, 2H), 8.07 – 8.01 (m, 2H), 7.72 – 7.66 (m, 1H), 7.65 – 7.58 (m, 2H), 7.16 – 7.11 (m, 2H), 3.46 (s, 3H), 2.32 (s, 3H). ^{13}C-NMR (101.6 MHz, $CDCl_3$): δ= 173.4, 169.2, 153.9, 139.1, 134.0, 133.4, 131.1, 129.8, 127.3, 121.3, 44.5, 21.3. IR ATR (ν_{max}/cm^{-1}): 3066, 3028, 2928, 2166, 1982, 1756, 1618, 1584, 1502, 1480, 1448, 1414, 1370, 1332, 1312, 1290, 1196, 1158, 1140, 1090, 1046, 1016, 984, 916, 864, 834, 810, 780, 762, 744, 704, 684, 582, 530, 500. HRMS (*m/z*): [MNa]$^+$ calcd for $C_{16}H_{15}NO_4SNa$: 340.0614; found: 340.0614.

methyl 4-((methyl(oxo)(phenyl)-λ^6-sulfaneylidene)carbamoyl)benzoate (6r): The spectroscopic data agree with those reported in the literature [39-40]. Conv.: > 99%. Yield: 68%. 108 mg, 0.3403 mmol; white solid. Rf: 0.24 and 0.48 (*n*-hexane/EtOAc 6:4 and *n*-hexane/acetone 6:4). ^{1}H-NMR (400 MHz, $CDCl_3$): δ= 8.26 – 8.18 (m, 2H), 8.09 – 8.00 (m, 4H), 7.72 – 7.66 (m, 1H), 7.62 (ddt, J = 8.3, 6.8, 1.4 Hz, 2H), 3.92 (s, 3H), 3.47 (s, 3H). ^{13}C-NMR (101.6 MHz, $CDCl_3$): δ= 173.4, 166.7, 139.6, 138.8, 134.1, 133.2, 129.9, 129.4 (x2), 127.2, 52.4, 44.4.

4-acetyl-*N*-(methyl(oxo)(phenyl)-λ$_6$-sulfaneylidene)benzamide (6s): The spectroscopic data agree with those reported in the literature [39]. Conv.: 30%. Yield: 20%. 30 mg, 0.0995 mmol; pinkish solid. Rf: 0.38 (*n*-hexane/EtOAc 6:4). ^{1}H-NMR (400 MHz, $CDCl_3$): δ= 8.27 – 8.21 (m, 2H), 8.09 – 8.04 (m, 2H), 8.01 – 7.96 (m, 2H), 7.74 – 7.69 (m, 1H), 7.67 – 7.61 (m, 2H), 3.49 (s, 3H), 2.64 (s, 3H). ^{13}C-NMR (101.6 MHz, $CDCl_3$): δ= 198.0, 173.4, 139.7, 139.6, 138.9, 134.1, 129.9, 129.8, 128.1, 127.3, 44.5, 27.0.

2.4. Large scale preparation of aroylated *NH*-sulfoximines model compound (6a) under flow conditions:

A solution of 4-iodotoluene (1.09 g, 5 mmol, 1 eq.), (methylsulfonimidoyl)benzene (**1**, 1.16 g, 0.75 mmol, 1.5 eq.), $Pd(OAc)_2$ (23 mg, 0.1 mmol, 0.02 eq.), *N*-xantphos (570 mg, 1 mmol, 0.2 eq.), and 1,4-diazabicyclo (2.2.2)octan (DABCO, 561 mg, 5 mmol, 1 eq.) dissolved in DMF (10 mL, 0.5 M) was pumped at 100 μL min^{-1} and mixed with a streaming of CO at 1.00 mL min^{-1} controlled by a MFC (1.25 mL min^{-1}). The reaction mixture was allowed to react inside a 10 mL coil-reactor for 40 minutes of residence time at 80 °C and 6 bar (BPR). After the death volume, the outcome was collected for further 140 minutes. The reaction crude was basified with NaOH (0.1 M) and extracted with CH_2Cl_2 (x3). The combined organic layers were washed with an aqueous solution of LiCl 20% *w/v*, brine, dried over anhydrous Na_2SO_4, and concentrated under reduced pressure. The reaction crude was characterized by ^{1}H-NMR (ns: 1) determining quant. conversion. The reaction mixture was purified by flash chromatography by using as solvent system *n*-hexane/EtOAc (from 100:0 to 70:30) obtaining 1.30 g as a white solid confirmed by NMR analysis (4.75 mmol, 95% yield).

3. Solid Oxide Electrolysis Cell (SOEC) set-up

The SOEC built by Huber Scientific was used for online CO production and consists of a dense YSZ tube closed on one end, which serves as electrolyte. The electrodes were applied in form of pastes and subsequently sintered at 1200°C for 3 h. The cathode was deposited on the outside of the YSZ tube and features a three-layer structure: a porous GDC layer acting as the electrochemically active layer, a GDC/Pt composite layer providing adhesion, and a porous Pt layer serving as the current collector. The latter two layers do not contribute catalytic activity, as already proven in earlier work [77, 82]. The air electrode on the inner side of the YSZ tube consists of Pt/YSZ paste (Tanaka, Japan). Both electrodes are electrically contacted using Pt wires, and the SOEC is placed inside a closely fitting quartz tube to minimise dead volume. This low dead volume of just a few cubic centimetres ensures very short flushing times.

To facilitate handling, a flexible damping element is integrated into the set-up. This element protects the ceramic tube from damage during installation and under increased mechanical stress. The tubular SOEC is connected to the gas lines by means of regular state-of-the art stainless steel tube fittings. Thereby the ceramic components are sealed using PTFE seals, whereas the quartz glass components are sealed with Viton O-rings. All sealing elements are designed for rapid and straightforward replacement. The sealed SOEC is installed in a tubular furnace equipped with a pressure gauge to monitor and control the absolute pressure. Temperature control is achieved using a PID controller in combination with a Type K thermocouple connected to a PC via a USB interface. The power supply providing the electrochemical pumping current can be operated manually or controlled via software when connected to a computer through a USB interface. A photo of the entire electrolysis set-up, with the SOEC already introduced into the tube furnace, is depicted in **Figure S2**.

For CO_2 electrolysis, the tube furnace was heated to 750°C. CO_2 was supplied to the cathode via a mass flow controller, and cathodic polarization of the GDC electrode enabled CO generation. To maintain a constant CO flux, polarization was carried out in galvanostatic mode. Prior to online coupling of the SOEC with the reactor, the CO concentration at the SOEC outlet was determined by a micro-gas chromatograph (Fusion 4-channel, Inficon, Switzerland), confirming a Faradaic efficiency of close to 1.

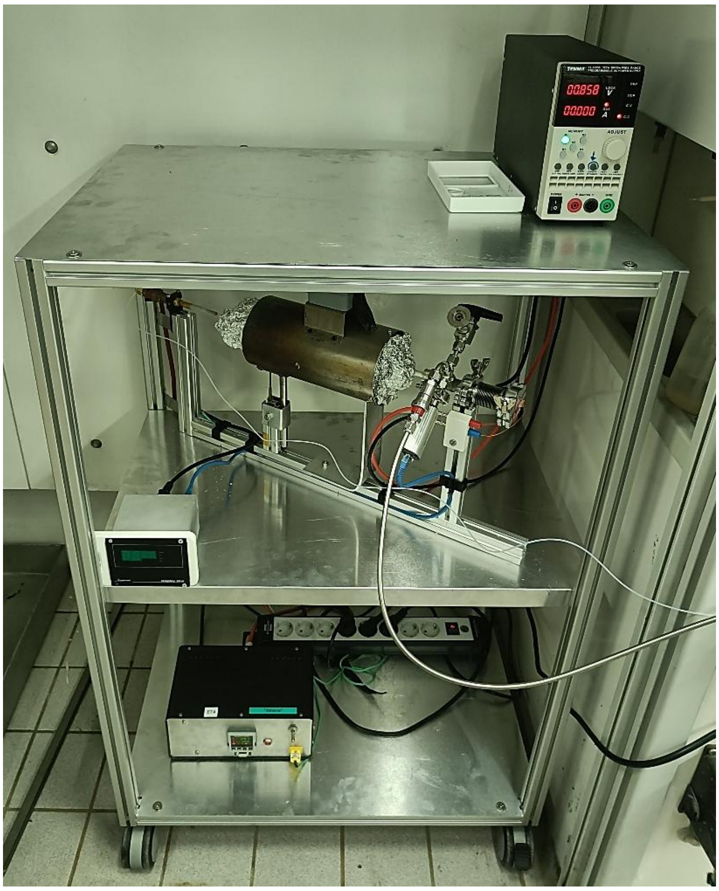

Figure S2. Solid Oxide Electrolysis Cell (SOEC) apparatus employed for the electrochemical reduction of CO_2.

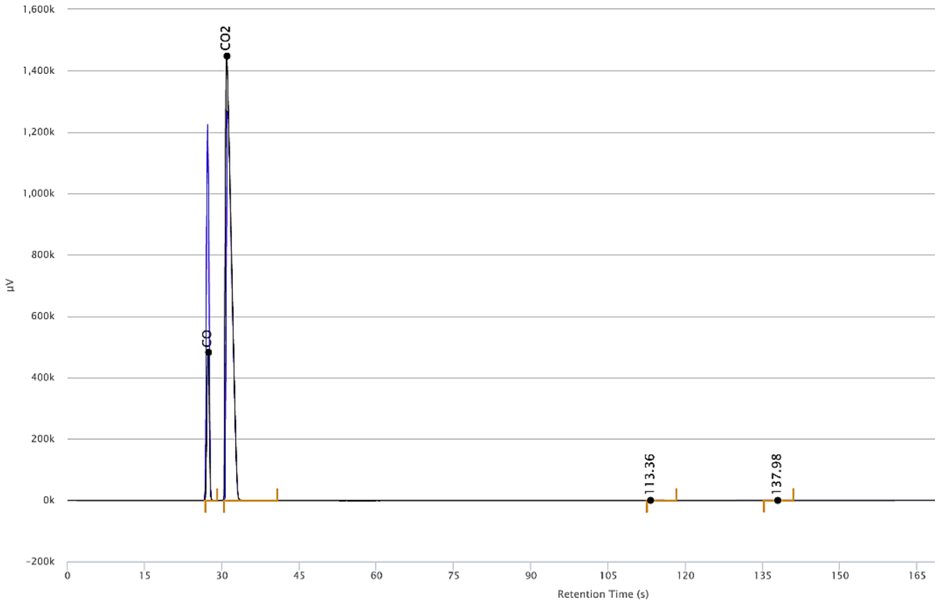

Figure S3. GC chromatograms of the outlet gas stream generated by the SOEC under a CO_2 flow rate of 7 sccm at pumping currents of 0.1 A (black line) and 0.3 A (purple line), corresponding to CO concentrations of approximately 11% and 32%, respectively. CO concentrations were quantified using calibration measurements performed with calibrated mass flow controllers (MFCs).

3.1 Experiments for aroylation of *NH*-sulfoximines under flow conditions by using CO/CO_2 gas mixture:

A vial with septum was charged with a solution of 4-iodotoluene (109 mg, 0.5 mmol, 1 eq.), (methylsulfonimidoyl)benzene (**1**, 116 mg, 0.75 mmol, 1.5 eq.), $Pd(OAc)_2$ (2.2 mg, 0.01 mmol, 0.02 eq.), *N*-xantphos (55 mg, 0.1 mmol. 0.2 eq.), and 1,4-diazabicyclo (2.2.2)octan (DABCO, 56 mg, 0.5 mmol, 1 eq.) dissolved in DMF (1 mL, 0.5 M). Pump A was used as a back-pressure regulator (BPR, 6 bar). Pump B was connected to the vial with the reaction mixture. Pump C was connected to the gas tube, where the pre-mixed gas mixture was introduced (see **Figure S1**). The gases were supplied from the respective cylinders and were pre-mixed with the aid of two mass flow controllers (MFCs). Alternatively, pump C was connected to SOEC at variable current corresponding to variable CO content and employing a flow rate of 8.0 mL min^{-1} (MFC). The tubing was primed with the reagent mixture and solvent, respectively. The 10 mL coil reactor was initially rinsed by a CO/CO_2/DMF flow for 30 minutes. Then, the reaction mixture was supplied to the reactor (pump B: 100 μL min^{-1}; pump C: 3.60 mL min^{-1} (or 8.0 mL min^{-1} if the electrochemical cell was employed) heated at 80 °C for τ= 40 min. The reaction mixture was basified with NaOH (0.1 M) and extracted with CH_2Cl_2 (x3). The combined organic layers were washed with an aqueous solution of LiCl 20% *w/v*, brine, dried over anhydrous Na_2SO_4, and concentrated under reduced pressure. The reaction crude was characterized by ^{1}H-NMR (ns: 1) determining the reaction conversion.

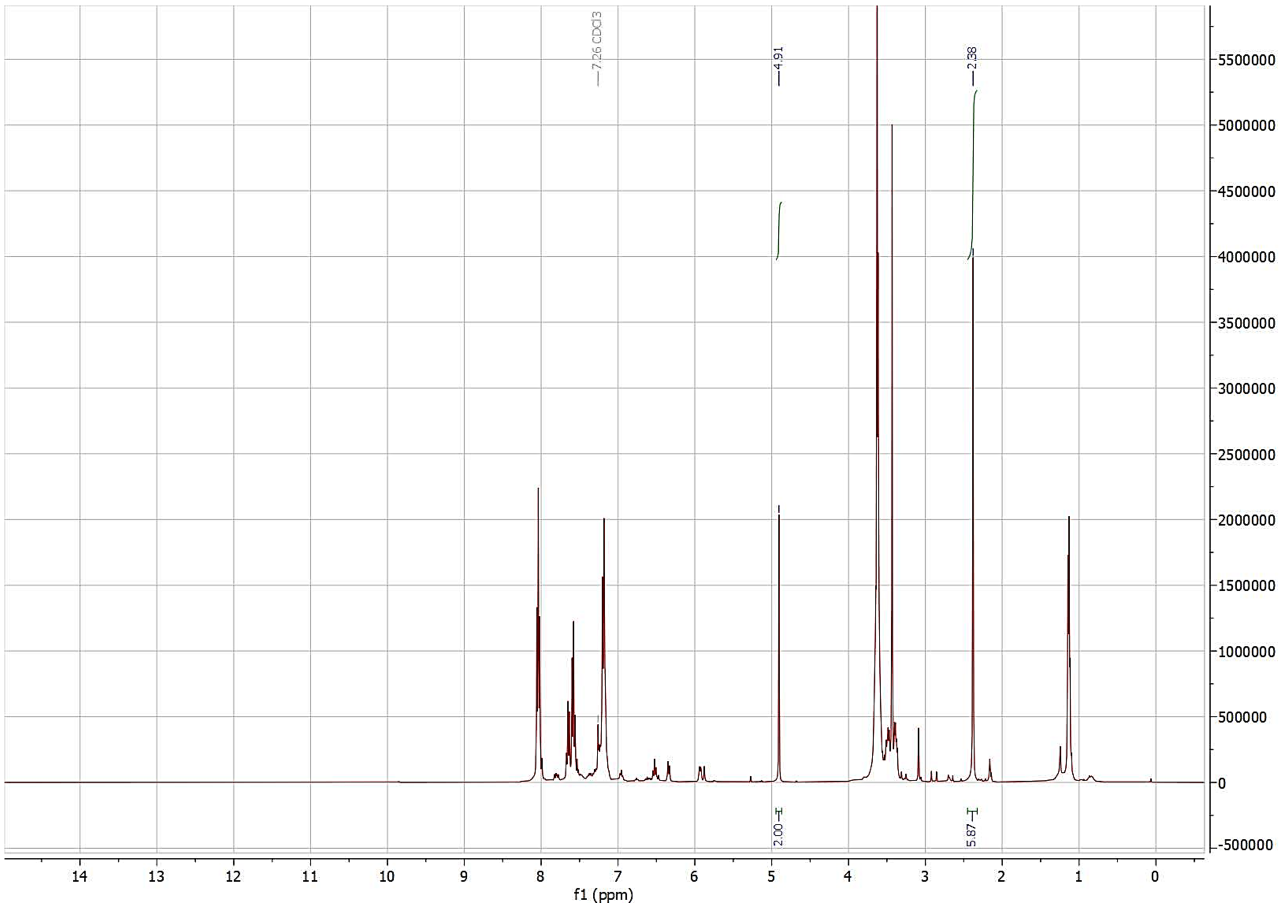

Figure S4. Quantitative ^{1}H-NMR spectrum of the crude reaction mixture ($CDCl_3$). Reaction performed under SOEC conditions (750 °C, 0.288 A, 2.0 V, corresponding to 28% CO). CH_2Br_2 (0.29 mmol) was used as internal standard. Quantification was performed by comparing the methyl signal of the tolyl group in **6a** (3H, s, 2.40 ppm) with the methylene signal of CH_2Br_2 (4.91 ppm).

4. Synthesis and characterization of heterogeneous Pd-based catalyst (7)

4.1. Synthetic procedures for the preparation of heterogeneous Pd-based catalyst (7) [83]

Step 1: *N*-xantphos (3.72 g, 6.76 mmol, 1.3 eq.) was dissolved in anhydrous THF (520 mL, 0.01 M) under inert atmosphere, then NaH (60% dispersed in mineral oil, 300 mg, 3.9 mmol, 1.5 eq.) was added to the solution and kept for 2 h at reflux under mechanical stirring. After that, Merrifield resin HL (100-200 mesh, loading= 1.3 mmol g^{-1}) (4.0 g, 5.20 mmol, 1 eq.) was added and the mixture was stirred at reflux for 16 h. The dark reaction mixture was filtered still warm under vacuum, and the functionalized resin was washed with fresh THF (100 mL), toluene (200 mL), H_2O (100 mL), and acetone (300 mL). The resulting functionalized resin was dried at 60 °C and 10-20 mbar for 12 h obtaining 7.70 g of a light brownish solid (**8**). The organic washing was collected and dried under vacuum. The washing was diluted with H_2O and extracted with CH_2Cl_2. The combined organic phases were washed with H_2O, brine, dried over anhydrous Na_2SO_4 and under reduced pressure obtaining 560 mg of a greyish solid. Theoretical loading (Merrifield resin + *N*-xantphos)= 1.3 mmol g^{-1}.

Step 2: To suspension of Merrifield resin + *N*-xantphos (7.00 g, 9.1 mmol, theoretical loading= 1.3 mmol g^{-1}, 1 eq.) in anhydrous THF (500 mL, 0.02 M), $Pd(OAc)_2$ (2.45 g, 10.92 mmol, 1.2 eq.) was added under inert atmosphere and the mixture was stirred at 25 °C for 72 h. After that, the resulting material was filtered through a Hirsh filter under inert atmosphere and washed with THF (100 mL x2) and acetone (100 mL x2). The solid was suspended with acetonitrile (100 mL x2) and stirred for 30 min at 25 °C. Then, was filtered through a Hirsh filter, resuspended with acetone (100 mL) and finally with CH_2Cl_2. The combined filtrates were concentrated under reduced pressure obtaining 412 mg of not complexed catalyst (dark oil). The supported *N*-xantphos complexed with palladium catalyst was dried under vacuum at 60 °C and 10-20 mmbar for 6 h obtaining 8.88 g as a brown/black solid (**7**). The solid was characterized by ICP analysis to determine Pd loading= 1.0688 mmol g^{-1}.

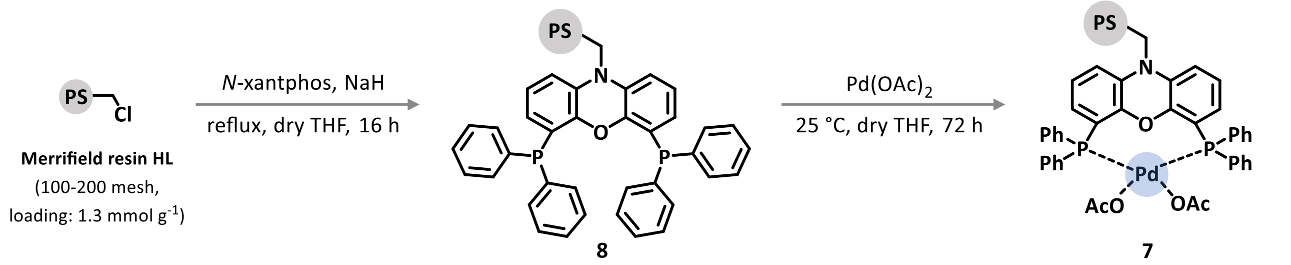

Scheme S1. Synthesis of heterogenous Pd-based catalyst **7**.

4.2. Characterization of heterogeneous Pd-based catalyst (7) and and its stability after flow reactions:

All reagents used for the measurements and digestion procedures were of analytical grade unless otherwise specified. Individual stock solutions of Pd (1000 ppm in 5% HCl) were obtained from Sigma-Aldrich (Germany) and used to prepare the calibration standards. Stock solution of In (1000 ppm in 2-3% HNO_3) was also supplied by Sigma-Aldrich (Germany). Concentrated HCl (37%), HNO_3 (65%), and HF (40%) were purchased from Merck (Germany), while H_2O_2 (30%) was obtained from Sigma-Aldrich (Germany). High-purity water (resistivity 18 MΩ·cm) was produced using an Easipure water purification system (Thermo, USA).

The Pd loading on the supported catalyst (**7**) was determined using a radial ICP-OES instrument (Agilent 5110, Agilent Technologies, Santa Clara, CA, USA). Prior to analysis, the supported catalyst (50 mg) was digested in a microwave system (Multiwave 5000, Anton Paar, Germany) using a mixture of mineral acids (7 mL HNO_3, 2 mL HCl, 1 mL H_2O_2) to ensure complete dissolution. The digestion program consisted of a 15 min ramp to 220 °C followed by a 20 min hold at the same temperature. A mixture of the recovered catalyst after flow experiment diluted with celite (50 mg) was digested separately under similar conditions using 0.8 mL HNO_3, 2.4 mL HCl, and 0.6 mL HF, with a 20 min ramp and a 40 min hold at 220 °C. After digestion, the samples were

appropriately diluted to obtain a final solution containing 2% (*v/v*) HCl. A six-point calibration curve was prepared for quantification. All measurements were performed within 24 h of digestion completion. Background-corrected emission signals were acquired and processed using ICP Expert software (Agilent, USA). The following emission lines were selected for data evaluation:

Element	Quantification line (nm)	Control lines (nm)
**Pd**	340.458	324.270, 342.122

4.3. General continuous flow procedure for aroylation of *NH*-sulfoximines by using heterogeneous Pd-based catalyst (7):

A solution of 4-iodotoluene (109 mg, 0.5 mmol, 1 eq.), (methylsulfonimidoyl)benzene (**1**, 116 mg, 0.75 mmol, 1.5 eq.), and 1,4-diazabicyclo (2.2.2)octan (DABCO, 56 mg, 0.5 mmol, 1 eq.) dissolved in DMF (1 mL, 0.5 M) was pumped (0.80 – 0.4 mL min^{-1}) and mixed with a streaming of CO (0.50 – 0.25 mL min^{-1}) controlled by a MFC (1.25 mL min^{-1}). A tubular reactor (DIBA 1.6 x 150 mm, bed volume mL= 0.3421 x bed high in cm (≃ 12 cm) = ≃ 4 mL after packing)) was packed with the heterogeneous catalyst (**7**, Pd loading= 1.0688 mmol g^{-1}, 56 mg, 0.05 mmol, 0.1 eq.) and diluted with solid inert support (950 mg for celite and 600 mg for Merrifield resin HL, 1:19 and 1:13 *w/w* ratios). The reaction mixture was carried out (τ= 2-4 min) at 80 °C and 6 bar (BPR). After the death volume, the outcome was collected for further 10 minutes. The crude was basified with NaOH (0.1 M) and extracted with Et_2O (x3). The combined organic layers were washed with an aqueous solution of LiCl 20% *w/v*, brine, dried over anhydrous Na_2SO_4, and concentrated under reduced pressure. The reaction crude was characterized by ^{1}H-NMR (ns: 1) determining the reaction conversion. The recovered catalyst after flow reaction was characterized by ICP analysis to determine Pd loading= 0.8370 mmol g^{-1}.

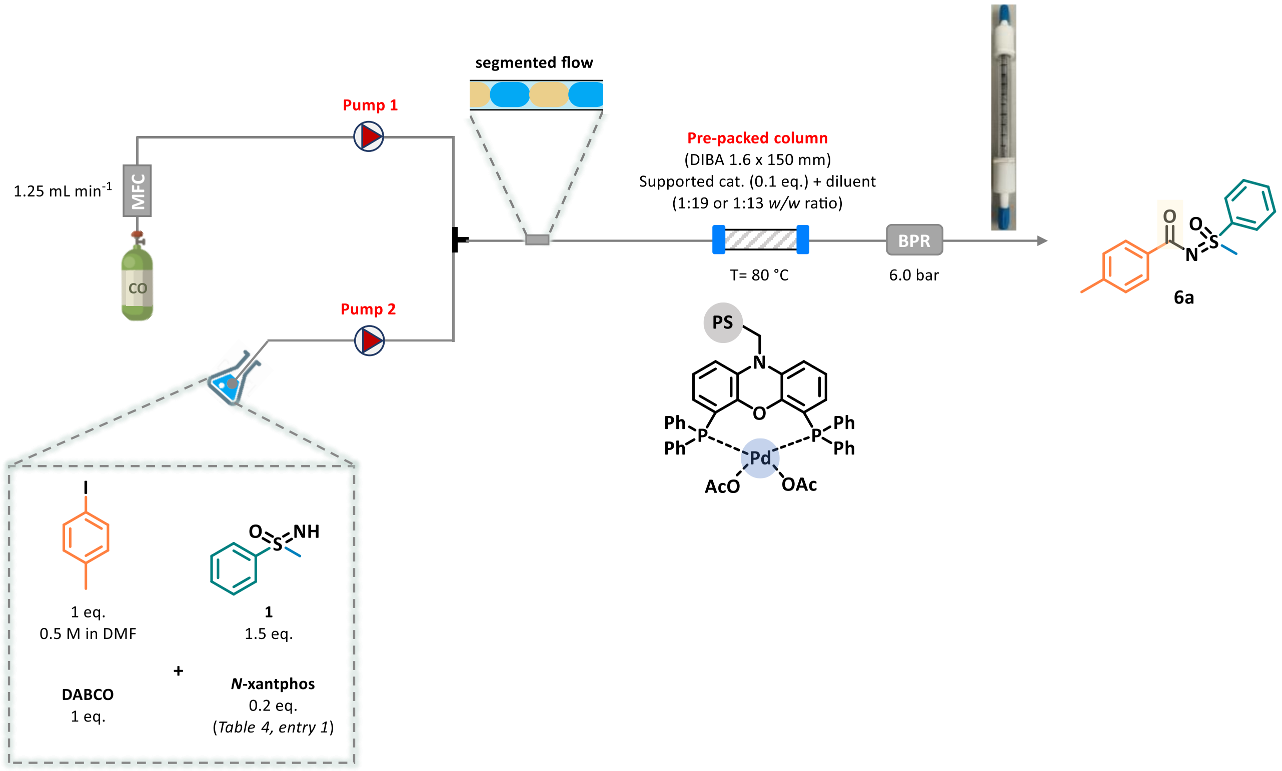

Scheme S2. Continuous flow procedure for the preparation of model compound **6a** by using heterogeneous Pd-based catalyst **7**.

5. Screening of Cyrene™ as solvent for *N*-aroylation of sulfoximines under flow conditions:

A solution of 4-iodotoluene (109 mg, 0.5 mmol, 1 eq.), (methylsulfonimidoyl)benzene (**1**, 116 mg, 0.75 mmol, 1.5 eq.), $Pd(OAc)_2$ (2.2 mg, 0.01 mmol, 0.02 eq.), *N*-xantphos (55 mg, 0.1 mmol, 0.2 eq.), and 1,4-diazabicyclo (2.2.2)octan (DABCO, 56 mg, 0.5 mmol, 1 eq.) dissolved in Cyrene™ (Dihydrolevoglucosenone, 1 mL, 0.5 M) was pumped at 100 µL min^{-1} and mixed with a streaming of CO at 1.00 mL min^{-1} controlled by a MFC (1.25 mL min^{-1}). The reaction mixture was allowed to react inside a 10 mL coil reactor for 40 minutes of residence time at 80 °C and 6 bar (BPR). An ultrasonic bath was positioned at the reactor outlet to prevent product precipitation at the junction. After the death volume, the outcome was collected for further 40 minutes. The precipitated desired compound (**6a**) was filtered, washed with Et_2O or acetone, and dried under reduced pressure.

6. Copy of NMR spectra

Compound 1

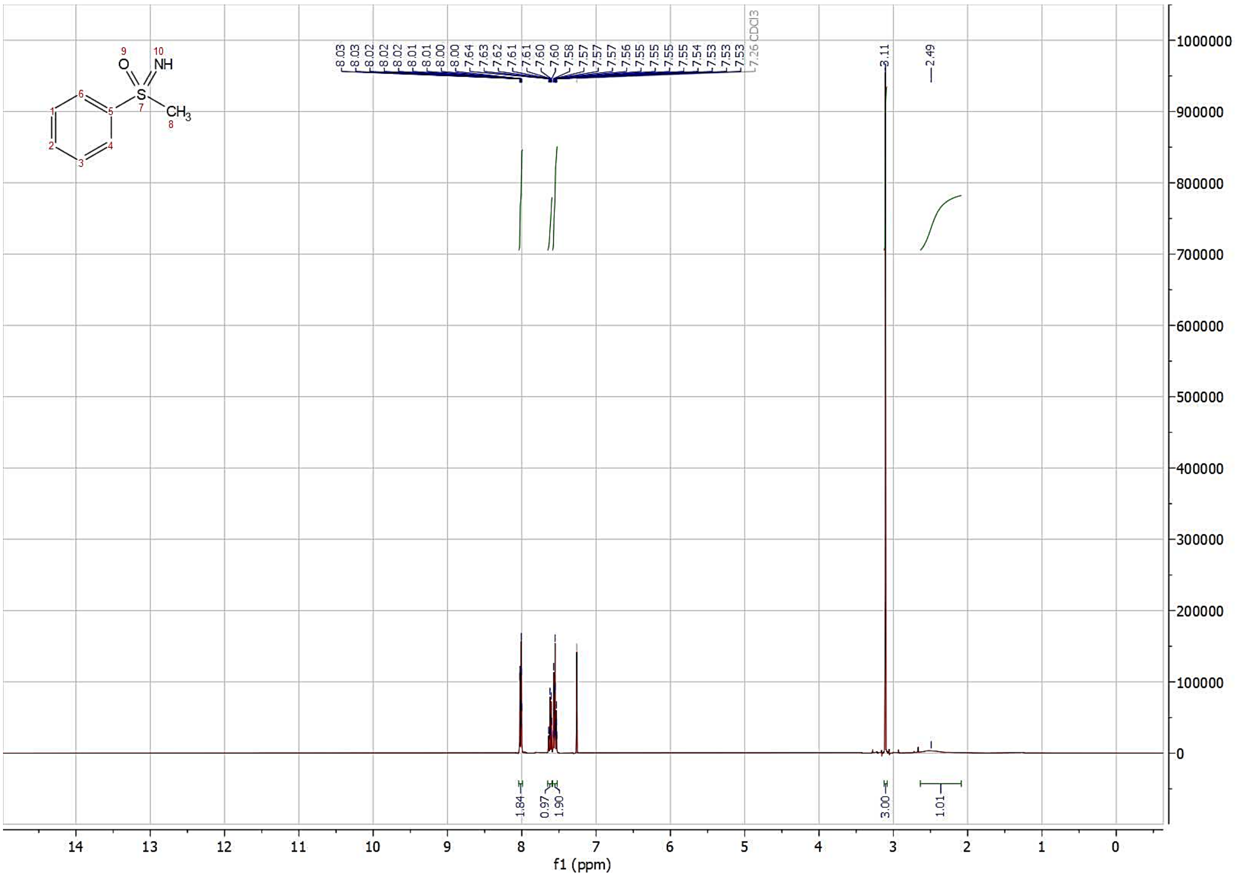

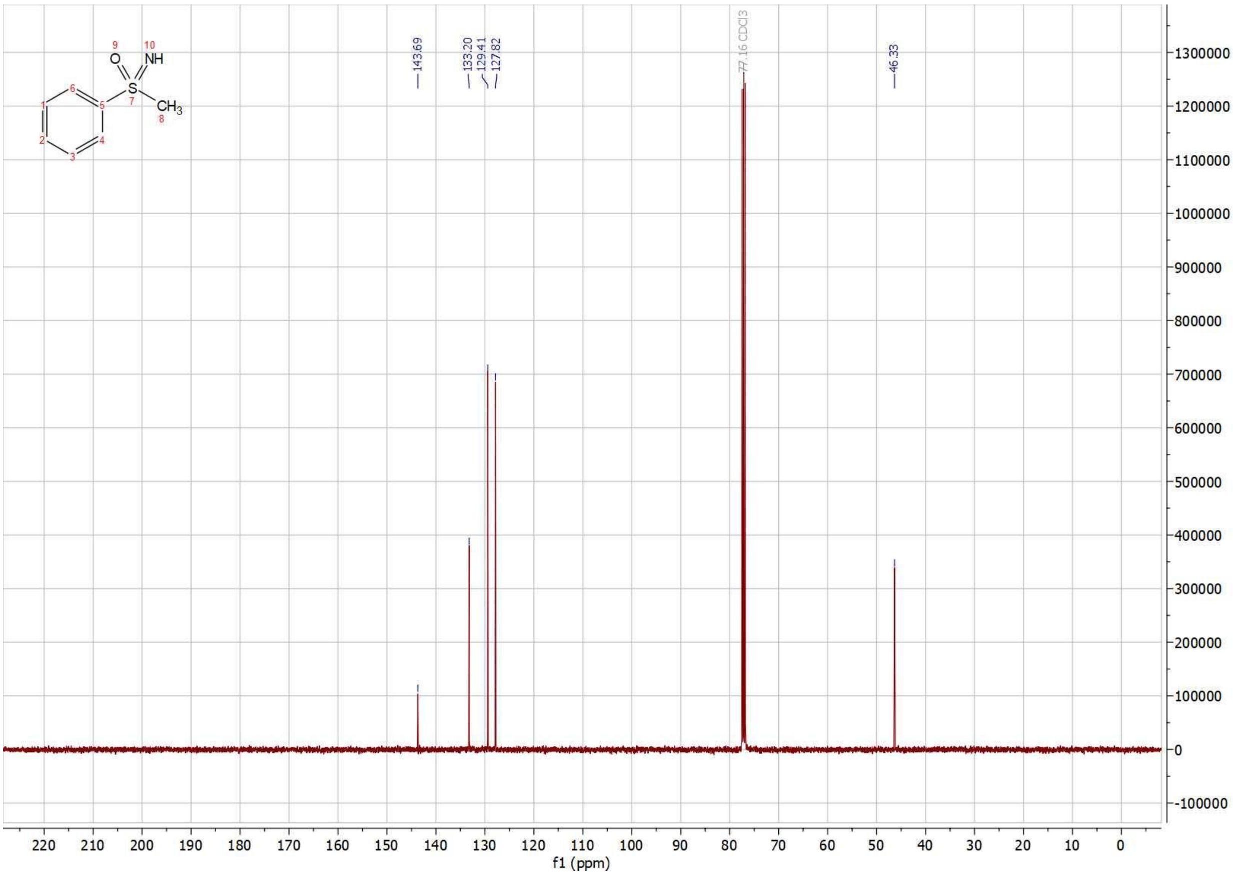

Compound 2

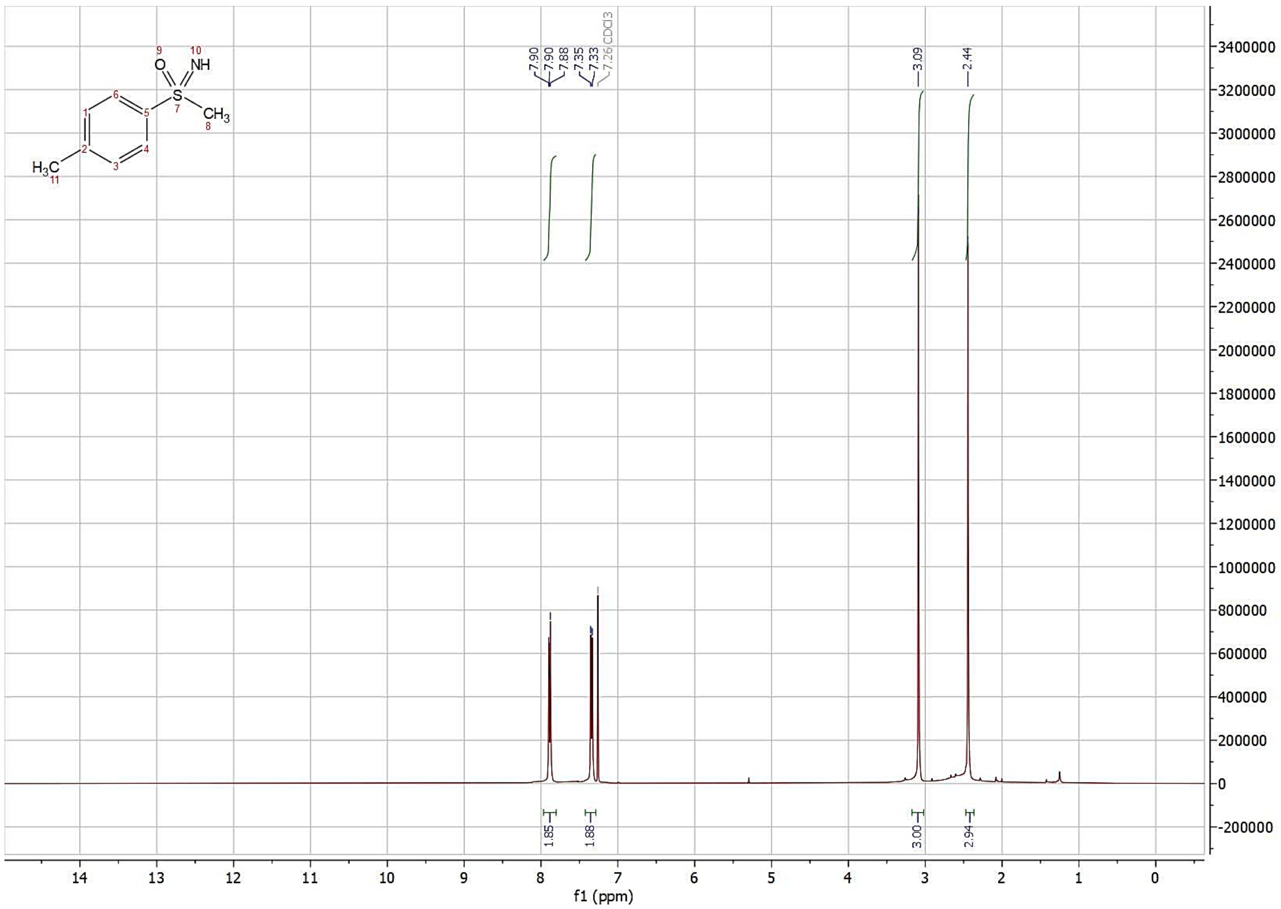

7.90
7.90
7.88
7.35
7.33
7.26 CDCl3
3.09
2.44
1.85
1.88
3.00
2.94
f1 (ppm)

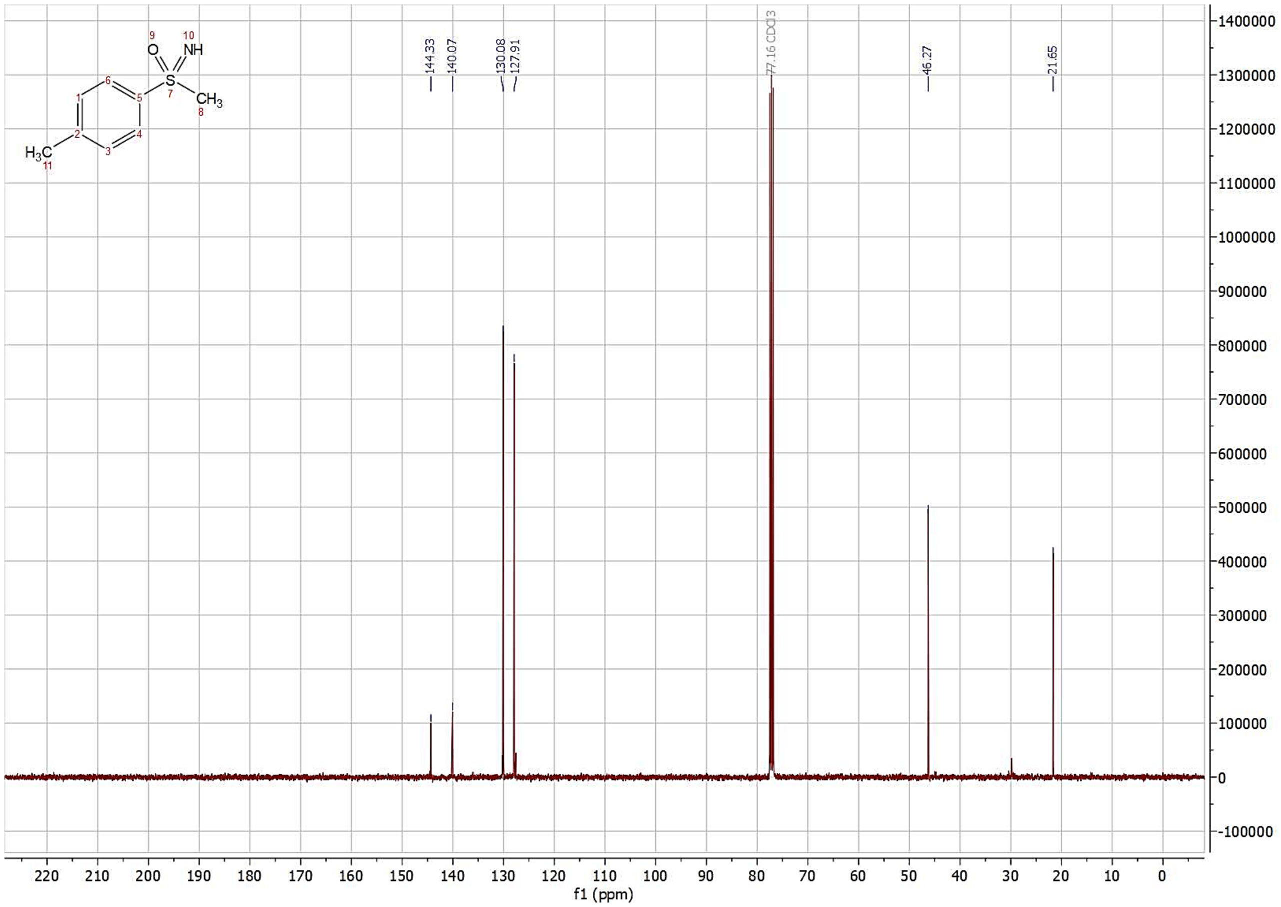

144.33
140.07
130.08
127.91
77.16 CDCl3
46.27
21.65
f1 (ppm)

Compound 3

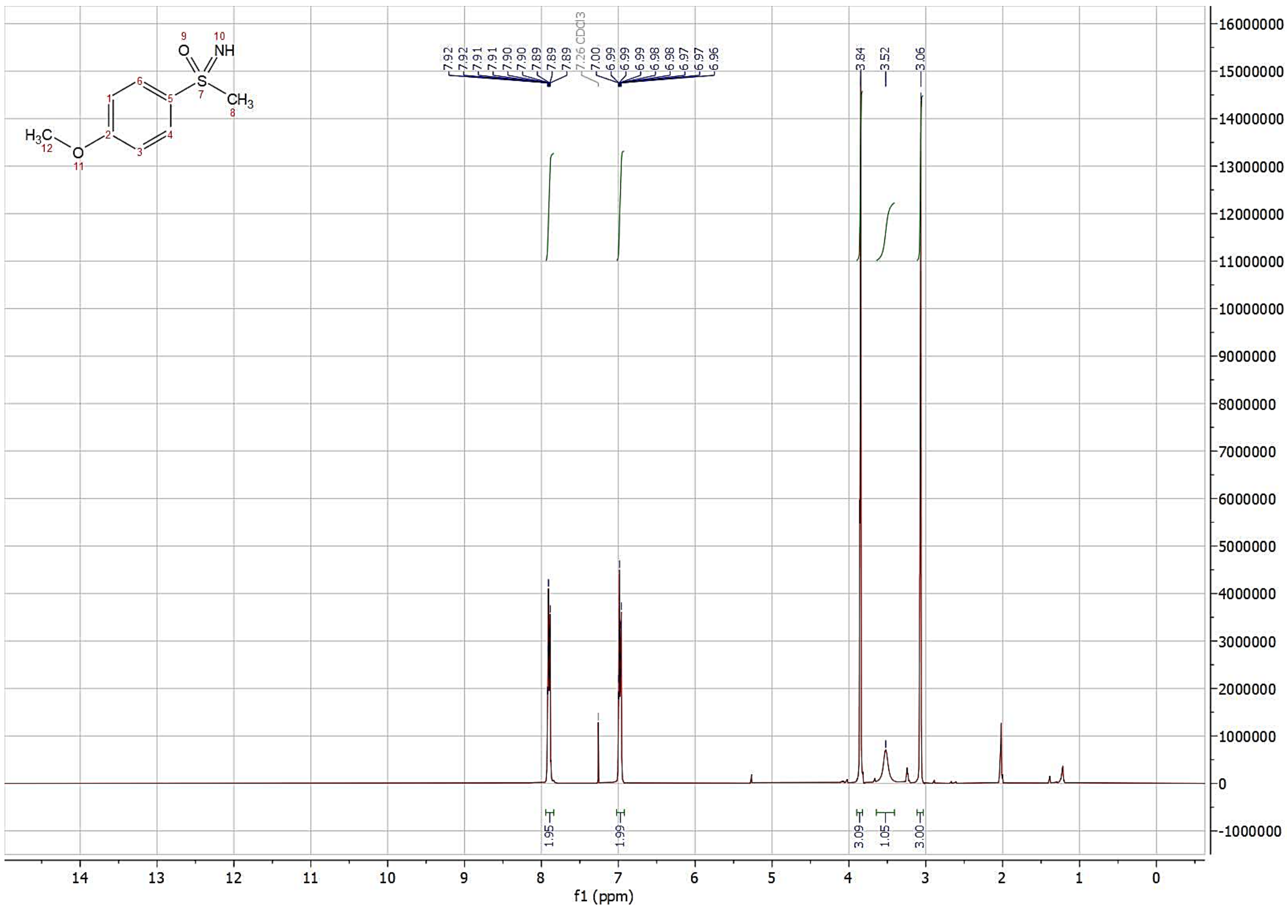

7.92
7.92
7.91
7.91
7.90
7.90
7.89
7.89
7.89
7.26 CDCl3
7.00
6.99
6.99
6.99
6.98
6.98
6.97
6.97
6.96
3.84
3.52
3.06
1.95
1.99
3.09
1.05
3.00
f1 (ppm)

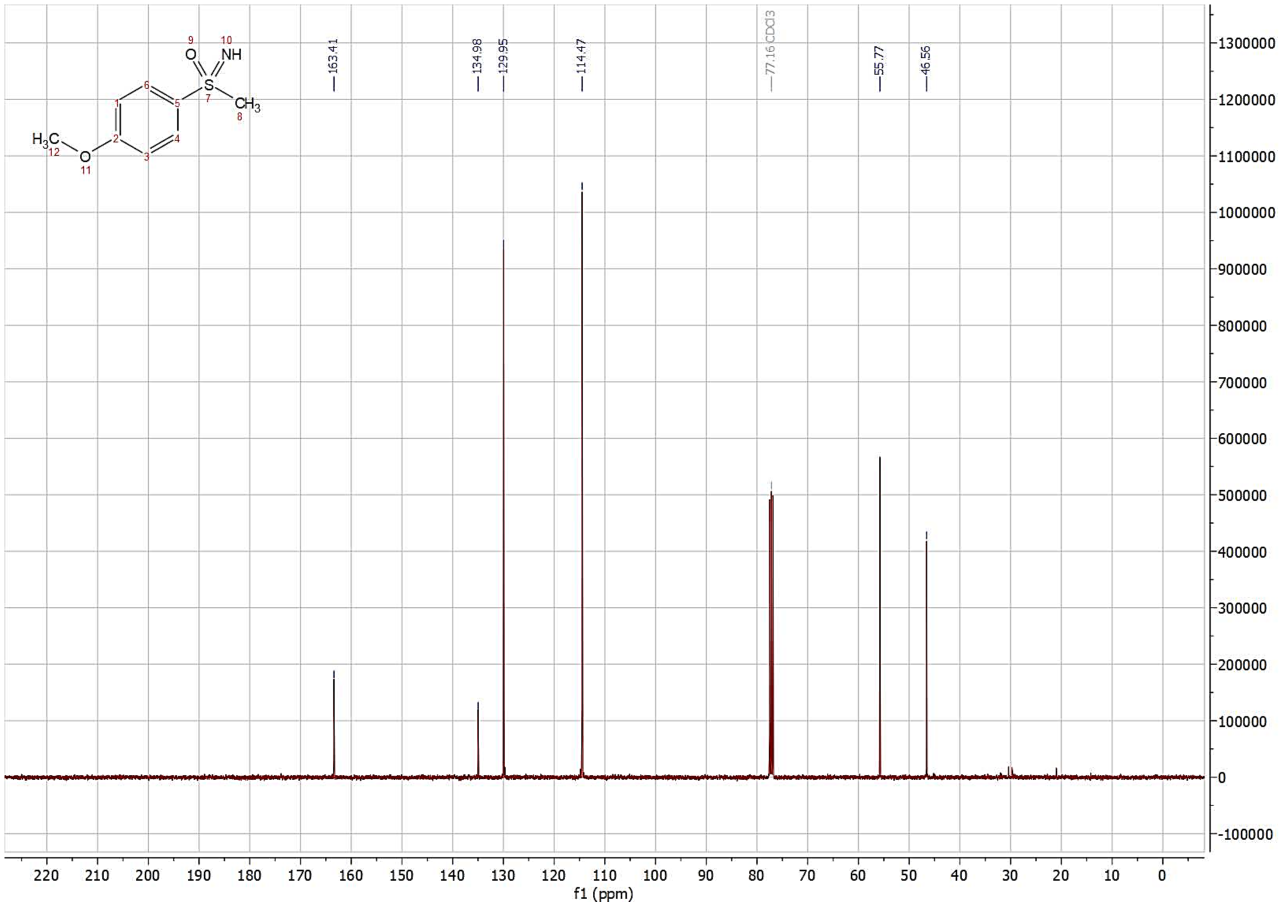

163.41
134.98
129.95
114.47
77.16 CDCl3
55.77
46.56
f1 (ppm)

Compound 4

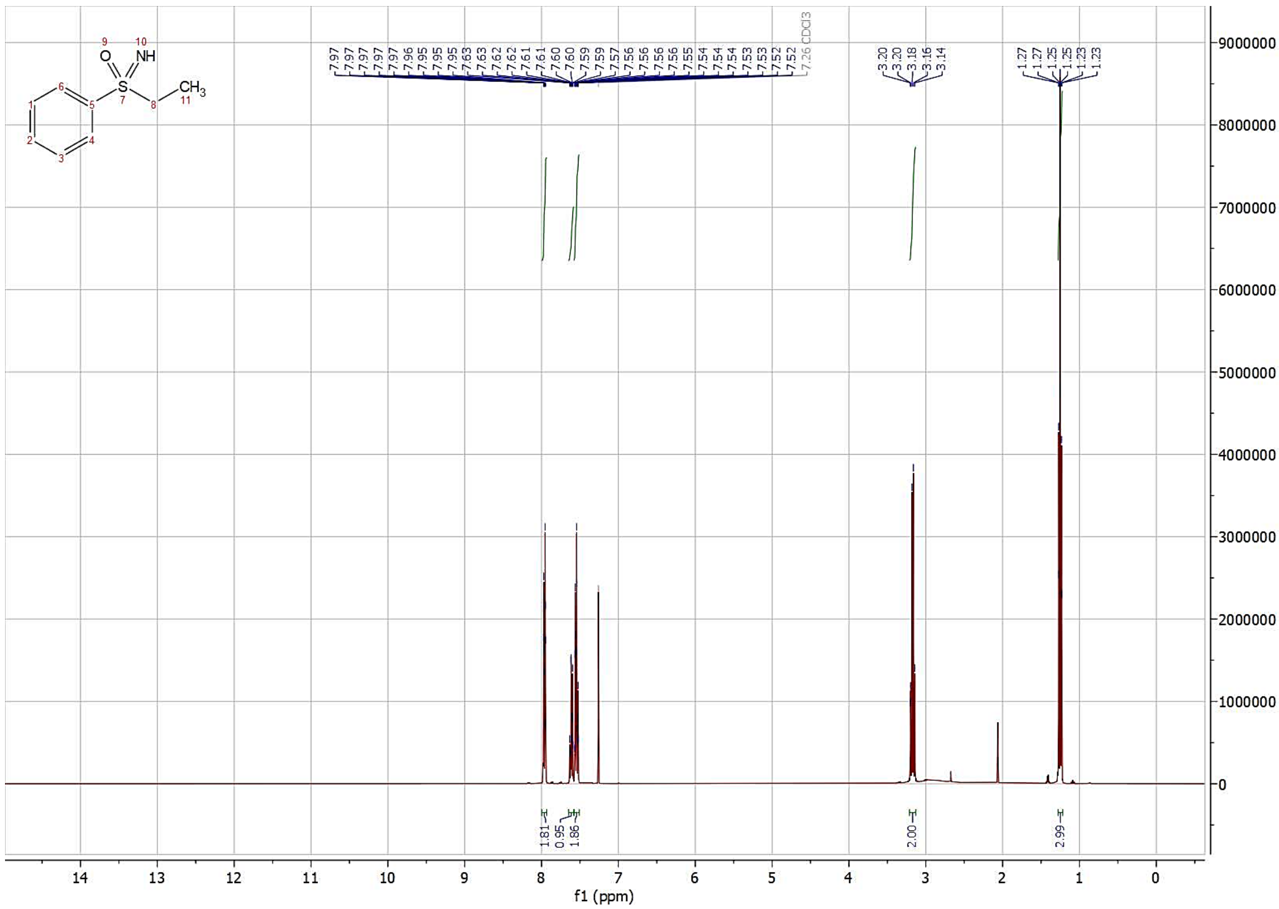

7.97
7.97
7.97
7.97
7.97
7.96
7.95
7.95
7.95
7.63
7.63
7.62
7.62
7.61
7.61
7.60
7.60
7.59
7.59
7.57
7.56
7.56
7.56
7.55
7.54
7.54
7.54
7.53
7.53
7.52
7.52
7.26 CDCl3
3.20
3.20
3.18
3.16
3.14
1.27
1.27
1.25
1.25
1.23
1.23
1.81
0.95
1.86
2.00
2.99
f1 (ppm)

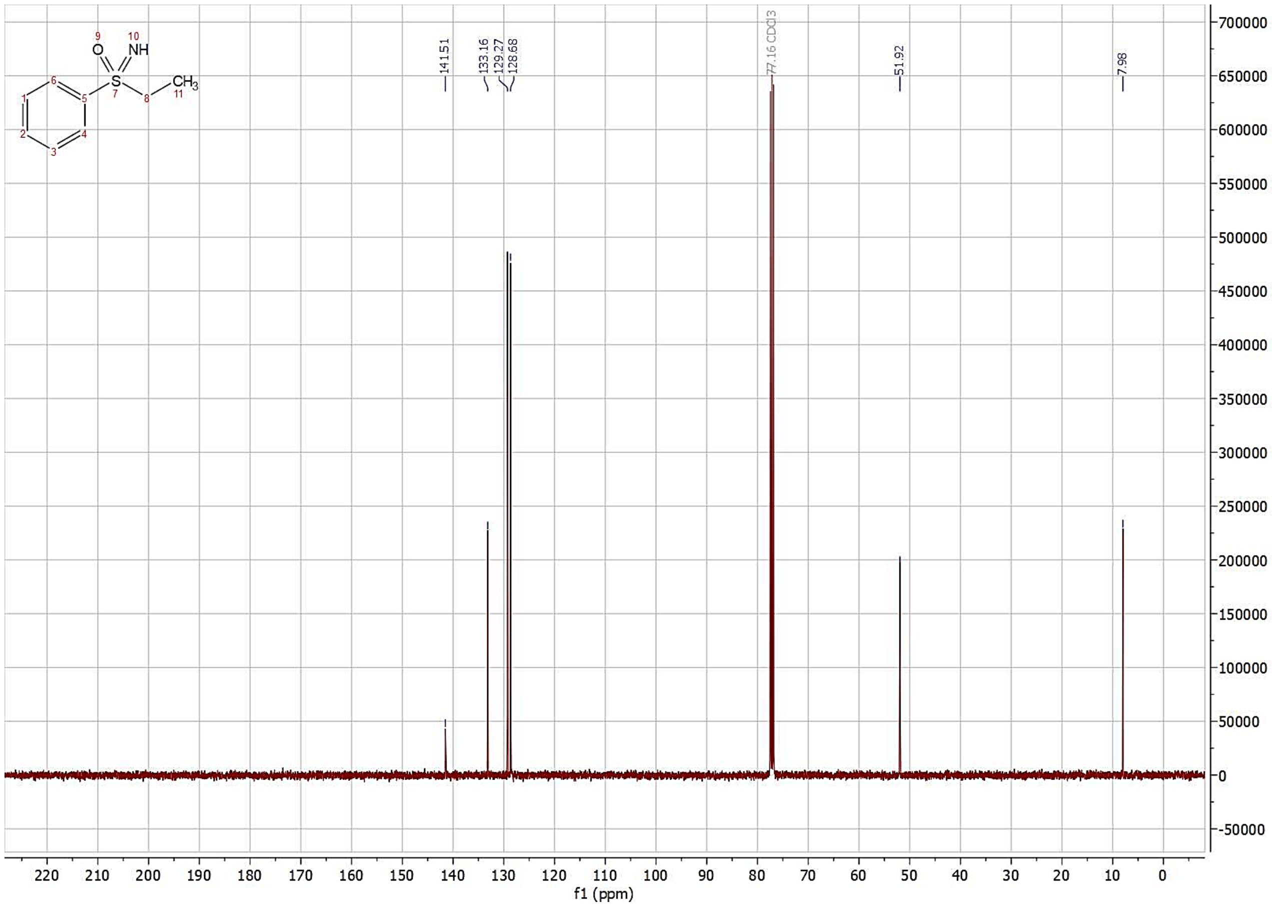

141.51
133.16
129.27
128.68
77.16 CDCl3
51.92
7.98
f1 (ppm)

Compound 5

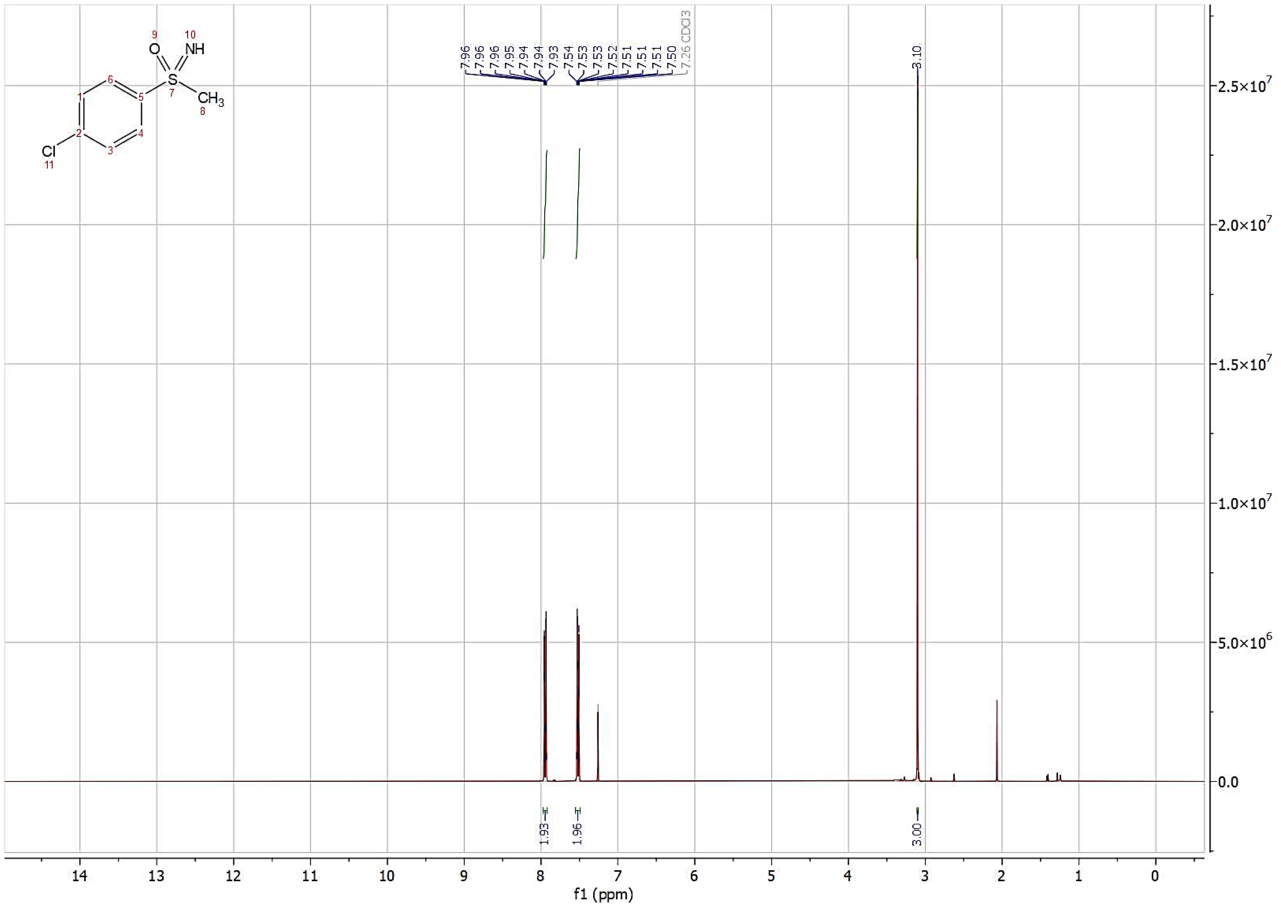

7.96
7.96
7.96
7.95
7.94
7.94
7.93
7.54
7.53
7.53
7.52
7.51
7.51
7.51
7.50
7.26 CDCl3
3.10
1.93
1.96
3.00
f1 (ppm)

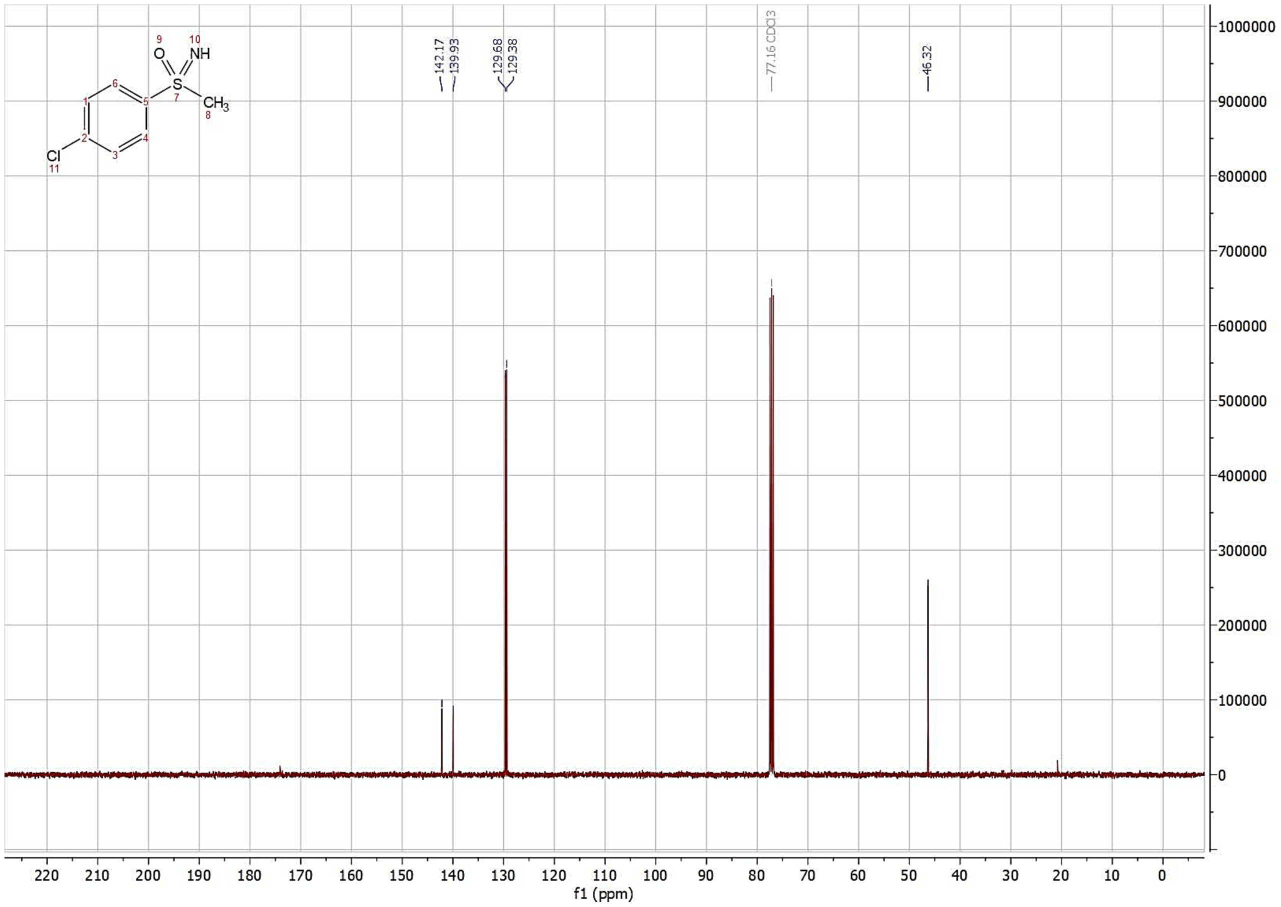

142.17
139.93
129.68
129.38
77.16 CDCl3
46.32
f1 (ppm)

Compound 6a

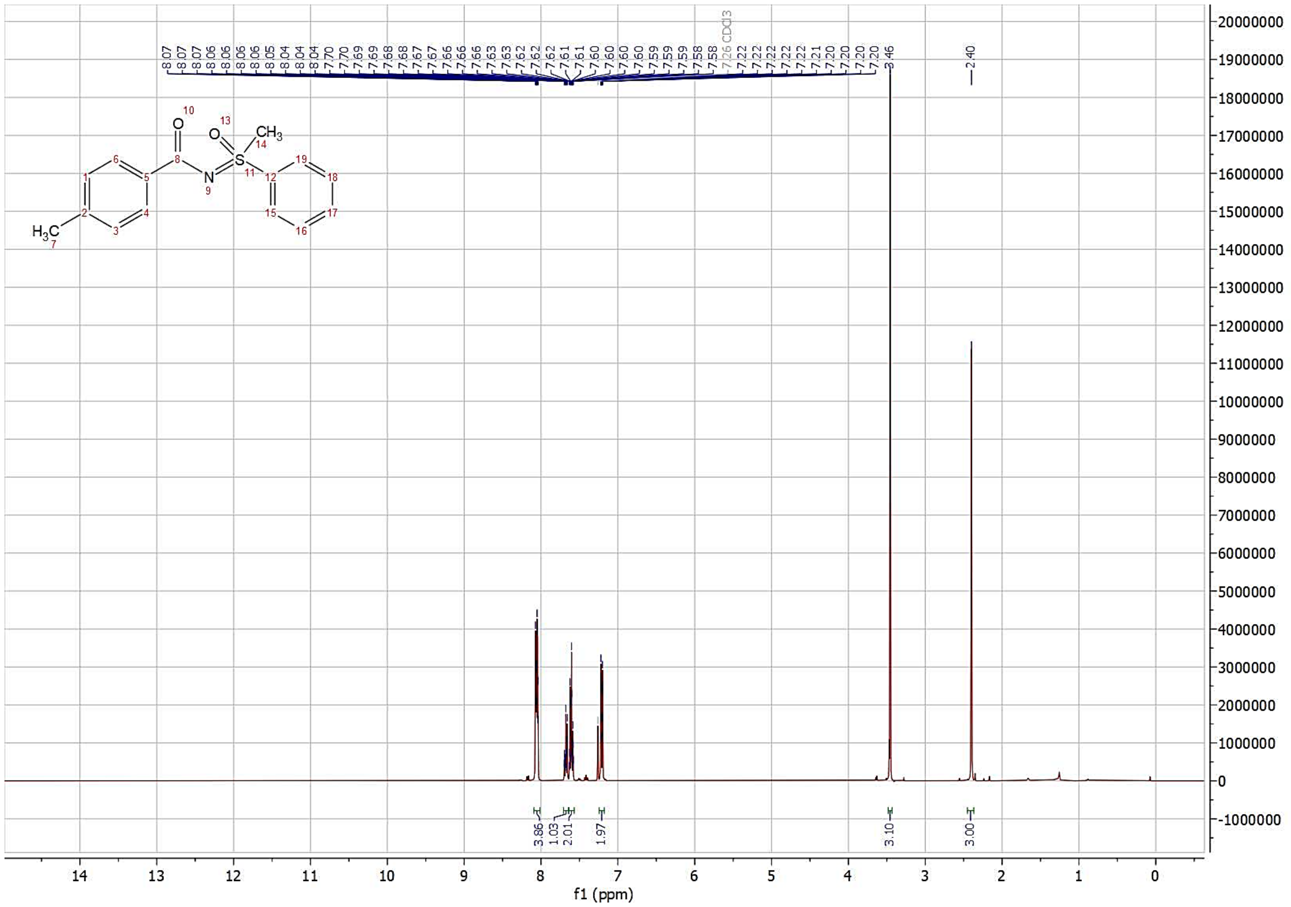

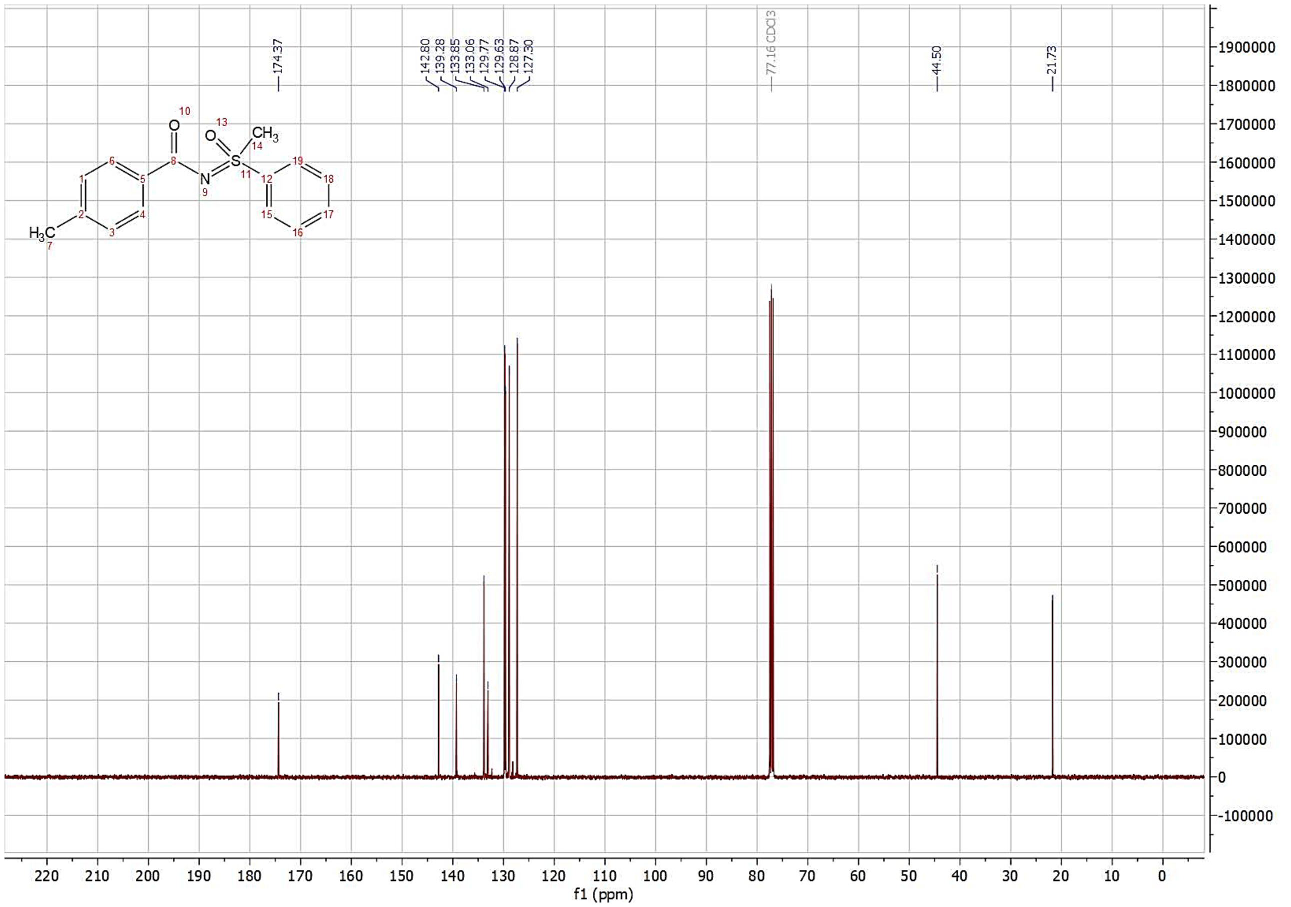

Compound 6b

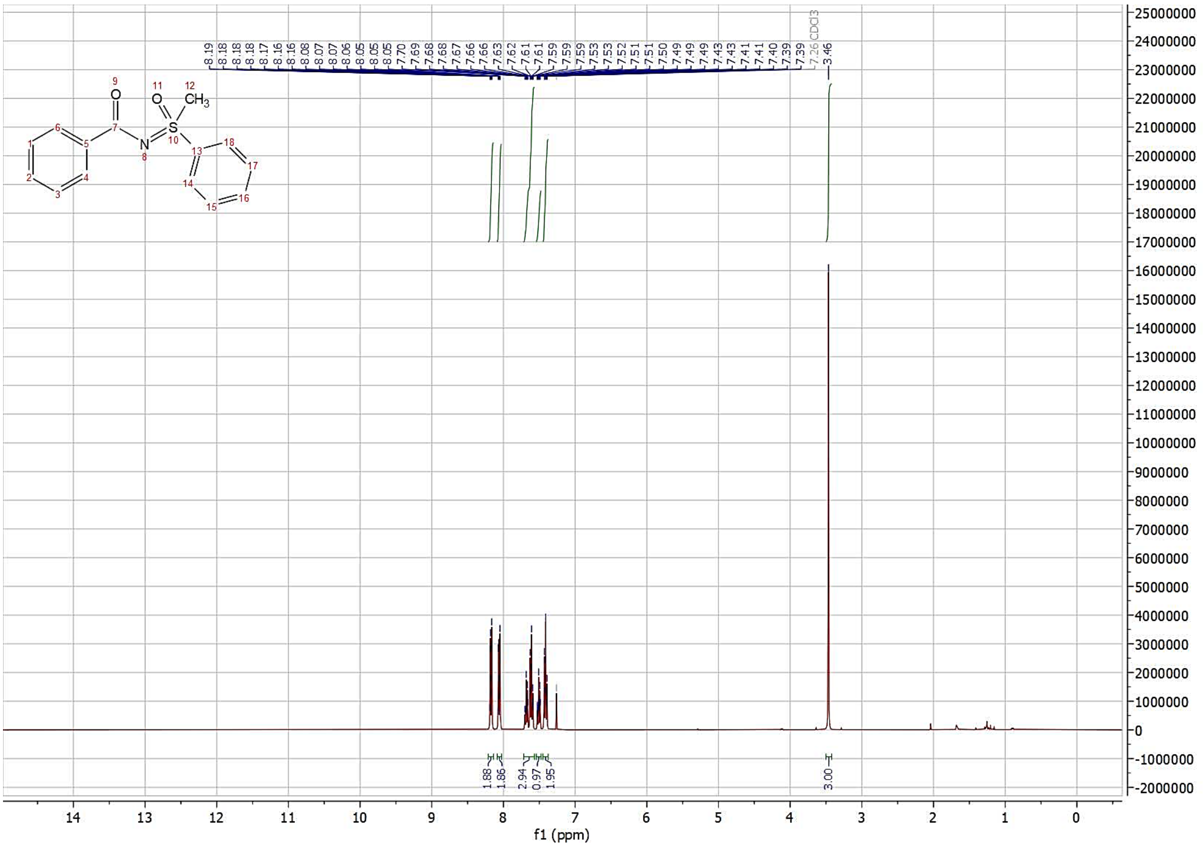
8.19
8.18
8.18
8.18
8.17
8.16
8.16
8.08
8.07
8.07
8.06
8.05
8.05
8.05
7.70
7.69
7.68
7.68
7.67
7.66
7.66
7.63
7.62
7.61
7.61
7.59
7.59
7.59
7.53
7.53
7.52
7.51
7.51
7.50
7.49
7.49
7.43
7.43
7.41
7.41
7.40
7.39
7.39
7.26 CDCl3
3.46
1.88
1.86
2.94
0.97
1.95
3.00
f1 (ppm)

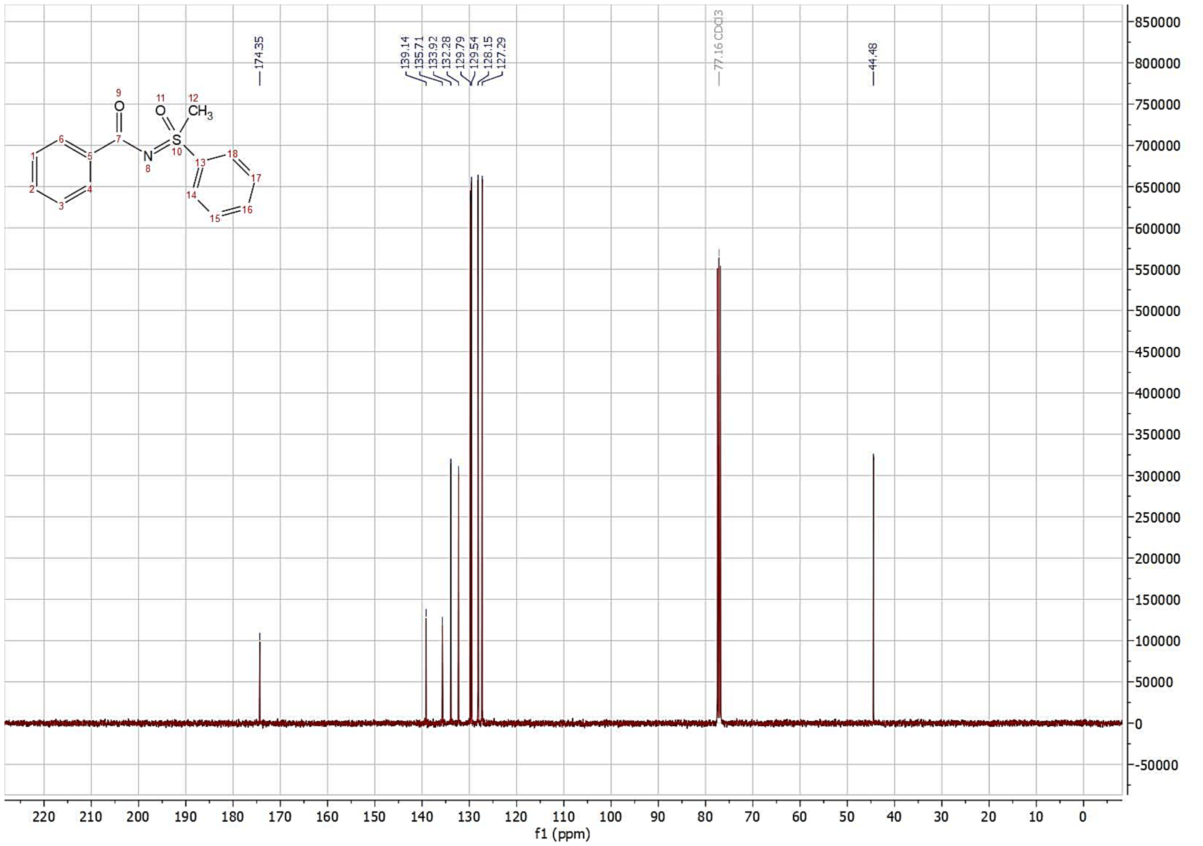
174.35
139.14
135.71
133.92
132.28
129.79
129.54
128.15
127.29
77.16 CDCl3
44.48
f1 (ppm)

Compound 6c

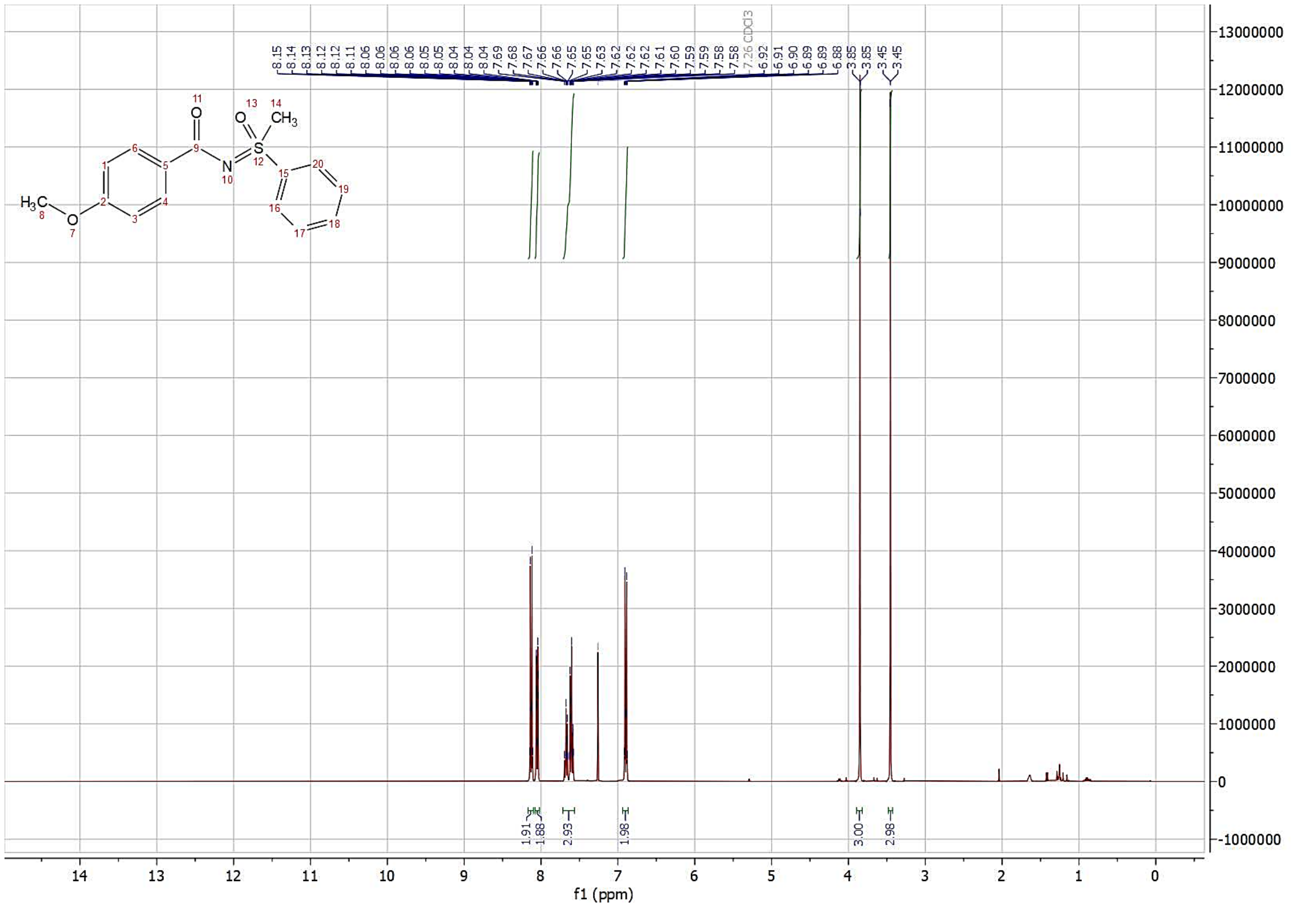

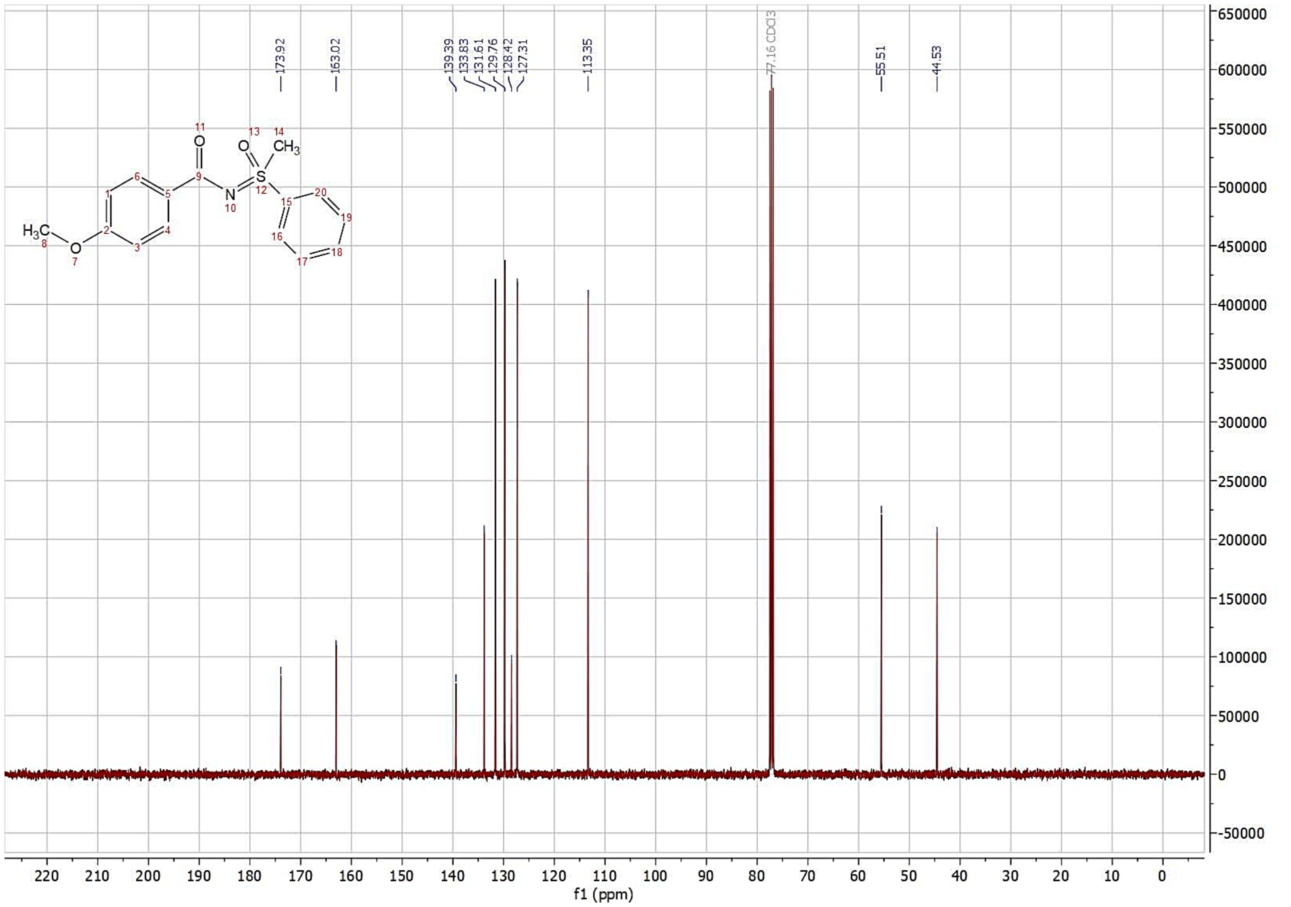

Compound 6d

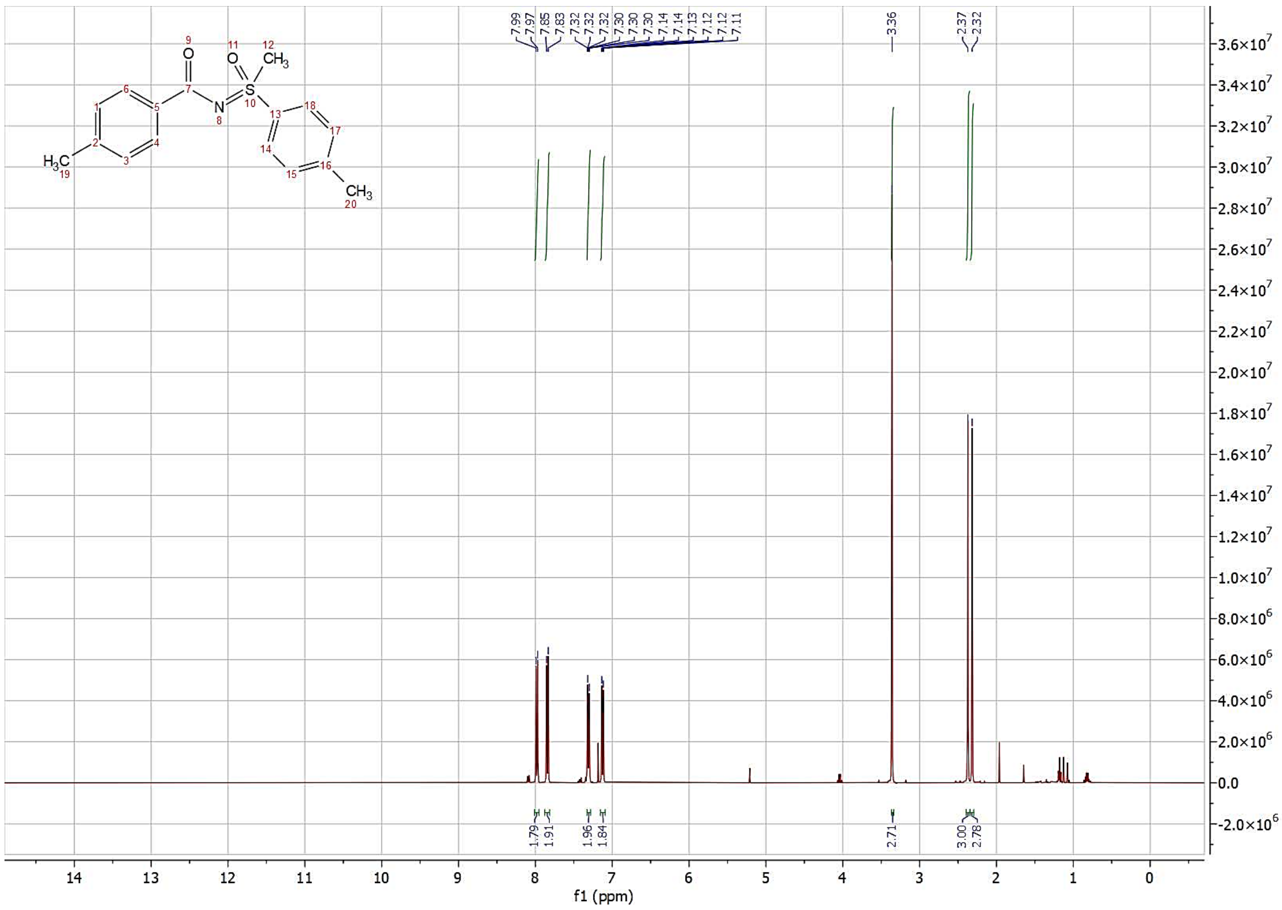

7.99
7.97
7.85
7.83
7.32
7.32
7.32
7.30
7.30
7.30
7.14
7.14
7.13
7.12
7.12
7.11
3.36
2.37
2.32
1.79
1.91
1.96
1.84
2.71
3.00
2.78
f1 (ppm)

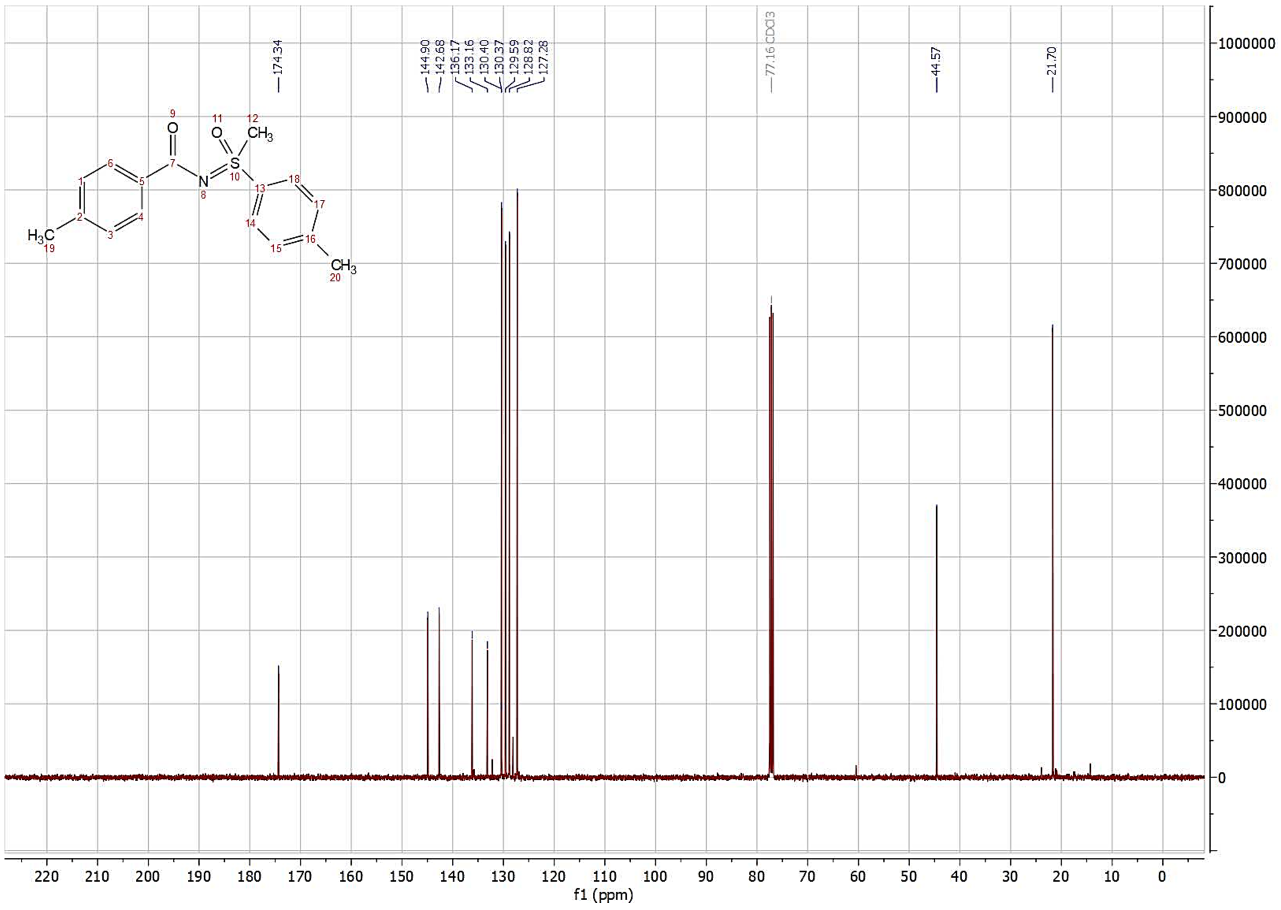

174.34
144.90
142.68
136.17
133.16
130.40
130.37
129.59
128.82
127.28
77.16 CDCl3
44.57
21.70
f1 (ppm)

Compound 6e

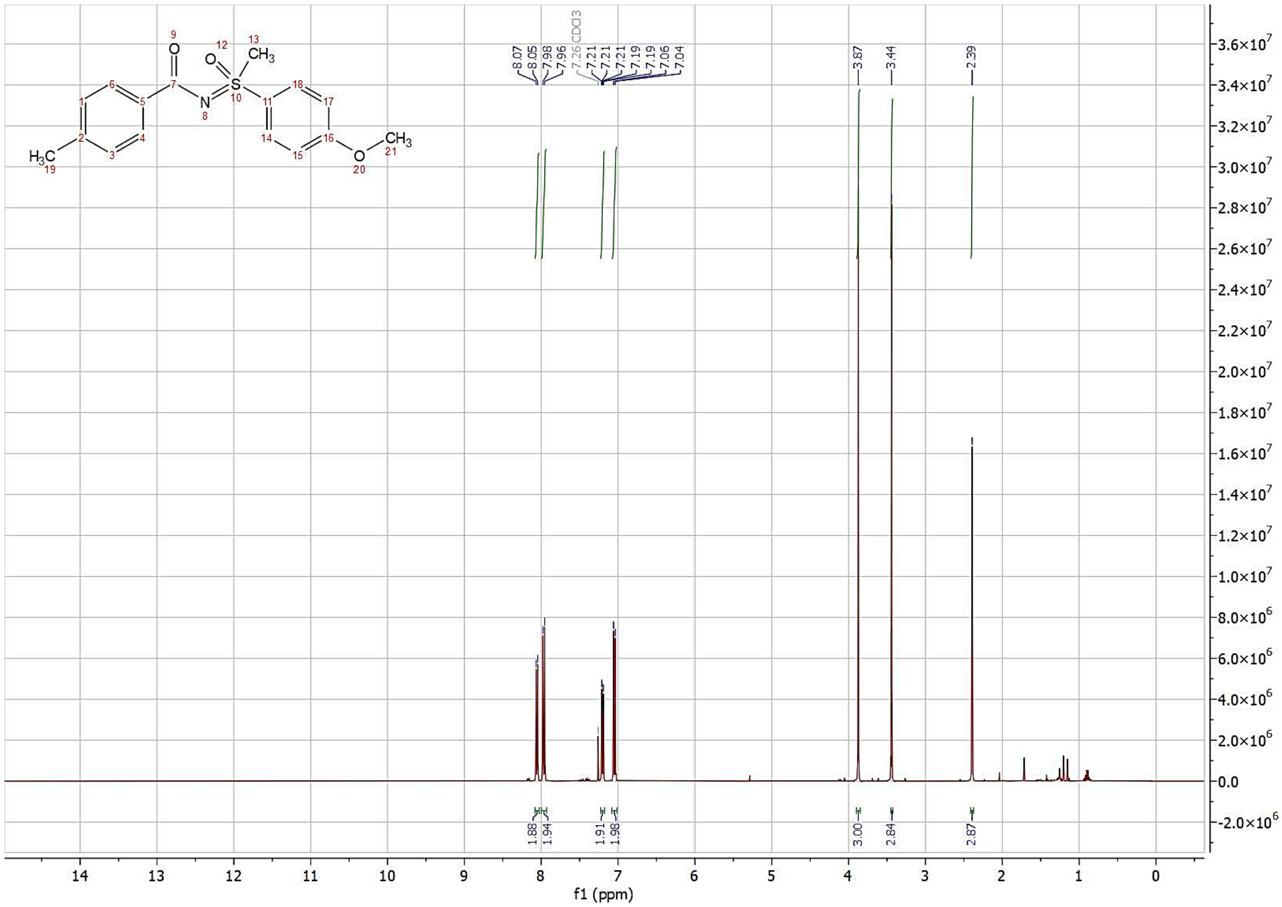

8.07
8.05
7.98
7.96
7.26 CDCl3
7.21
7.21
7.21
7.21
7.19
7.19
7.06
7.04
3.87
3.44
2.39
1.88
1.94
1.91
1.98
3.00
2.84
2.87
f1 (ppm)

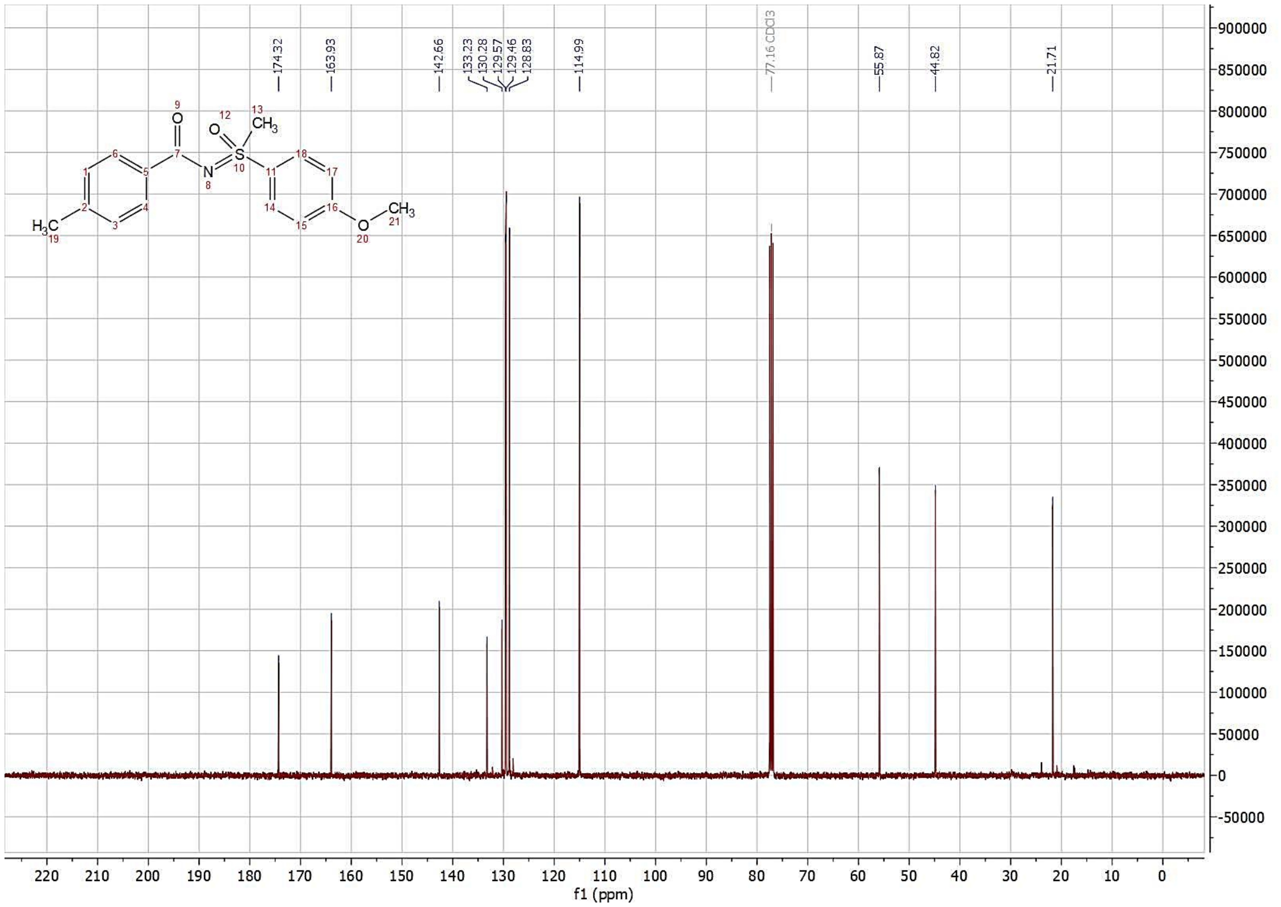

174.32
163.93
142.66
133.23
130.28
129.57
129.46
128.83
114.99
77.16 CDCl3
55.87
44.82
21.71
f1 (ppm)

Compound 6f

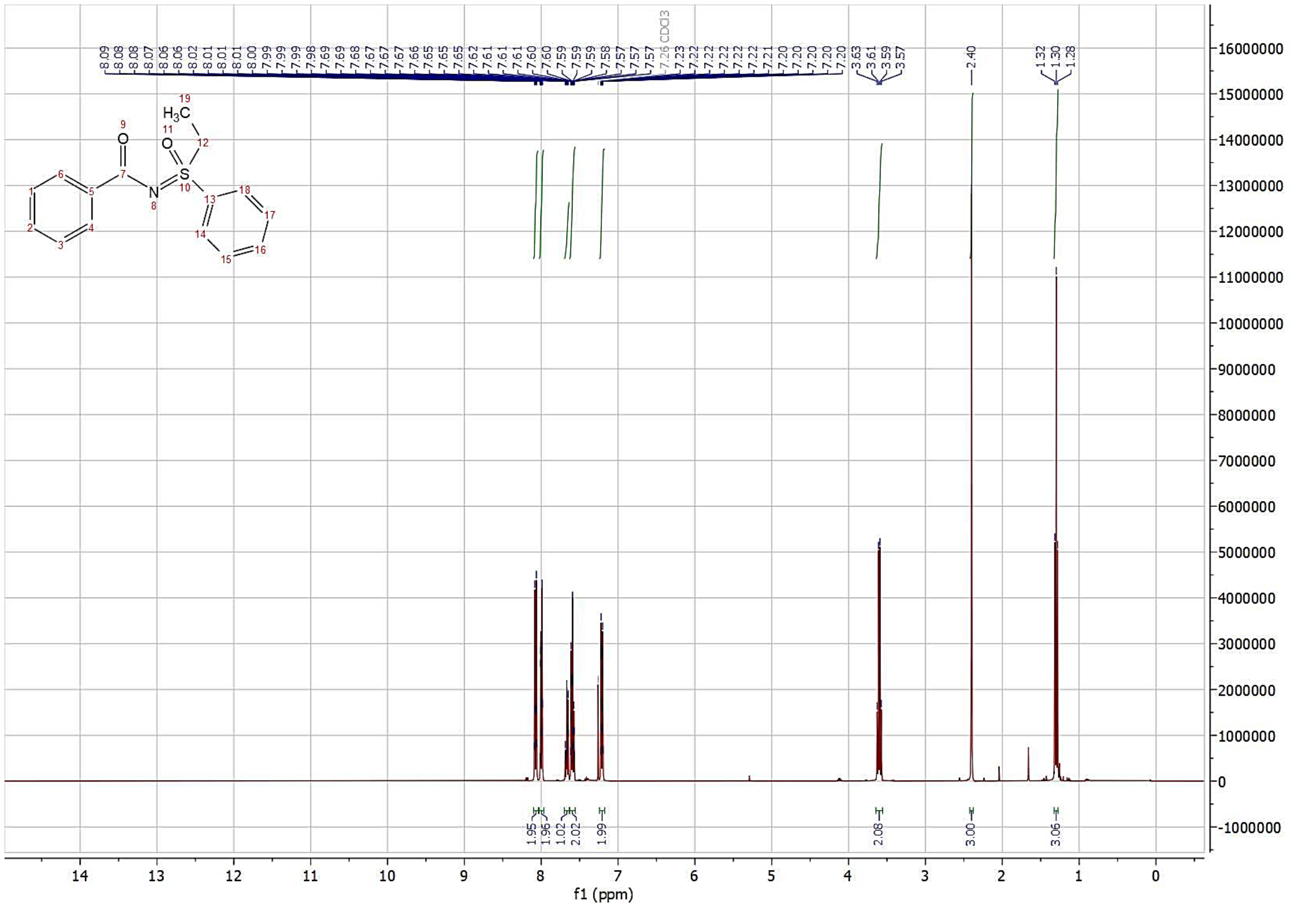

8.09 8.08 8.08 8.07 8.06 8.06 8.02 8.02 8.01 8.01 8.01 8.00 7.99 7.99 7.99 7.98 7.69 7.69 7.68 7.67 7.67 7.66 7.65 7.65 7.62 7.61 7.61 7.61 7.60 7.59 7.59 7.59 7.58 7.57 7.57 7.57 7.26 CDCl3 7.23 7.22 7.22 7.22 7.22 7.21 7.20 7.20 7.20 7.20 7.20
3.63 3.61 3.59 3.57
2.40
1.32 1.30 1.28
1.95 1.96 1.02 2.02 1.99 2.08 3.00 3.06
f1 (ppm)

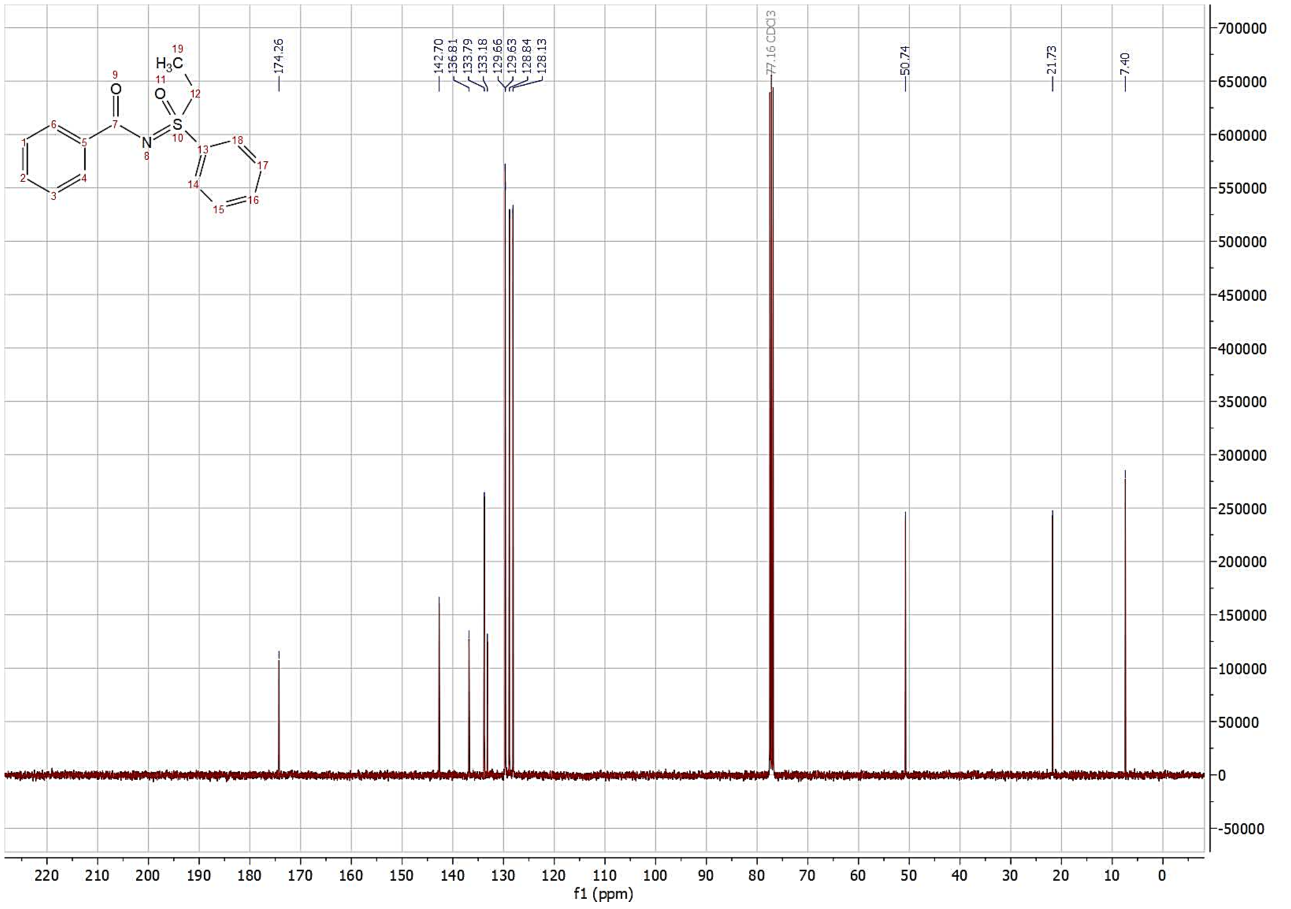

174.26
142.70 136.81 133.79 133.18 129.66 129.63 128.84 128.13
77.16 CDCl3
50.74
21.73
7.40
f1 (ppm)

Compound 6g

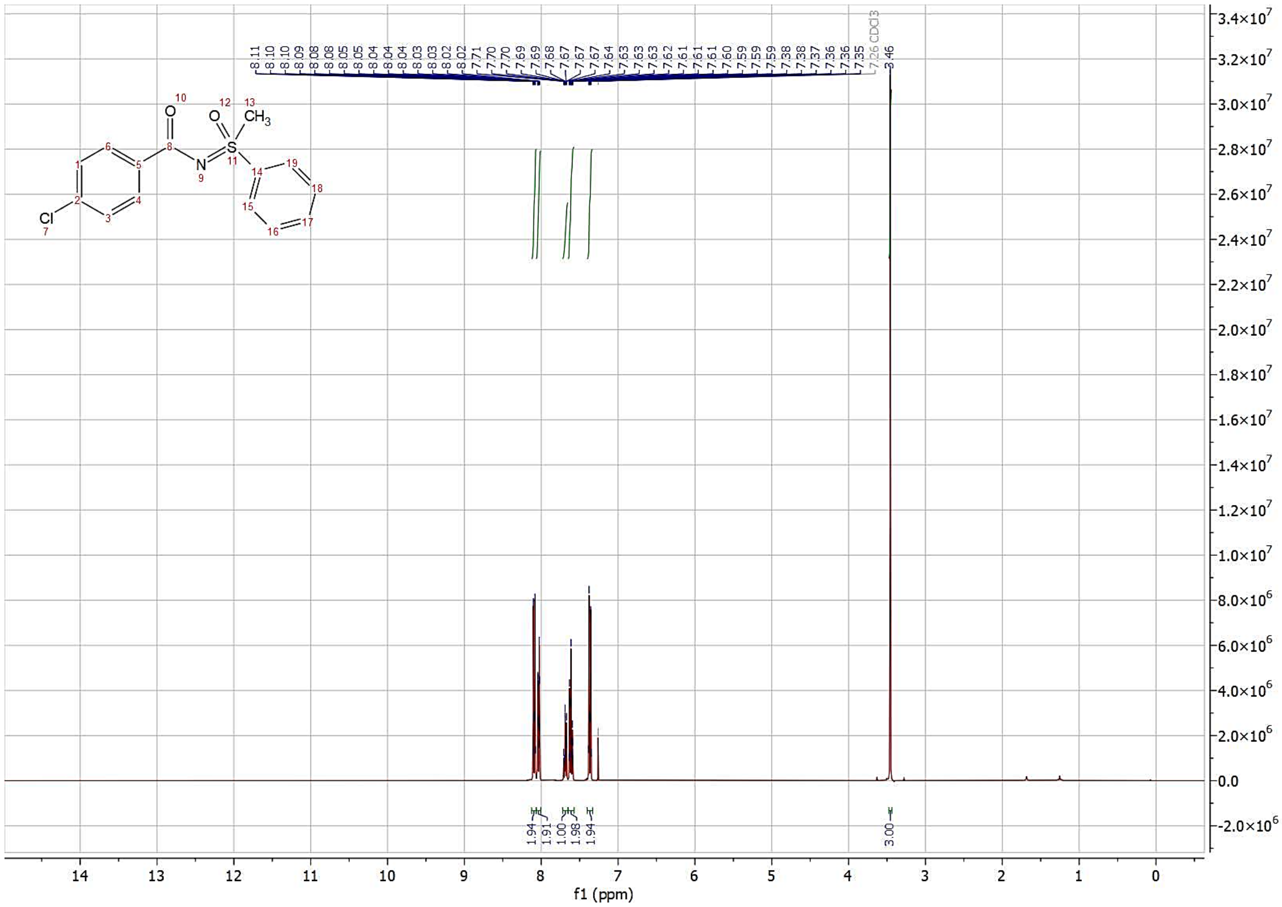

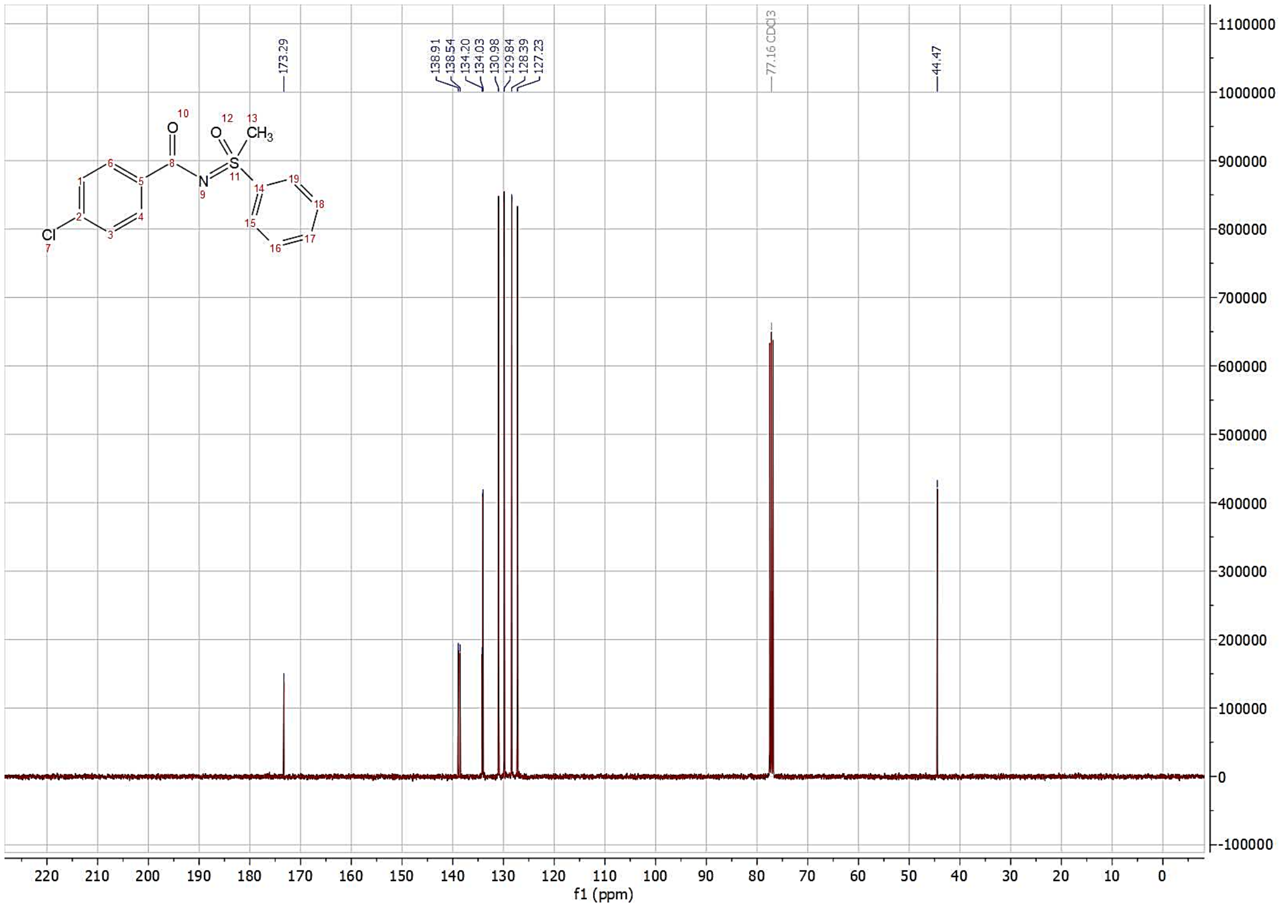

Compound 6h

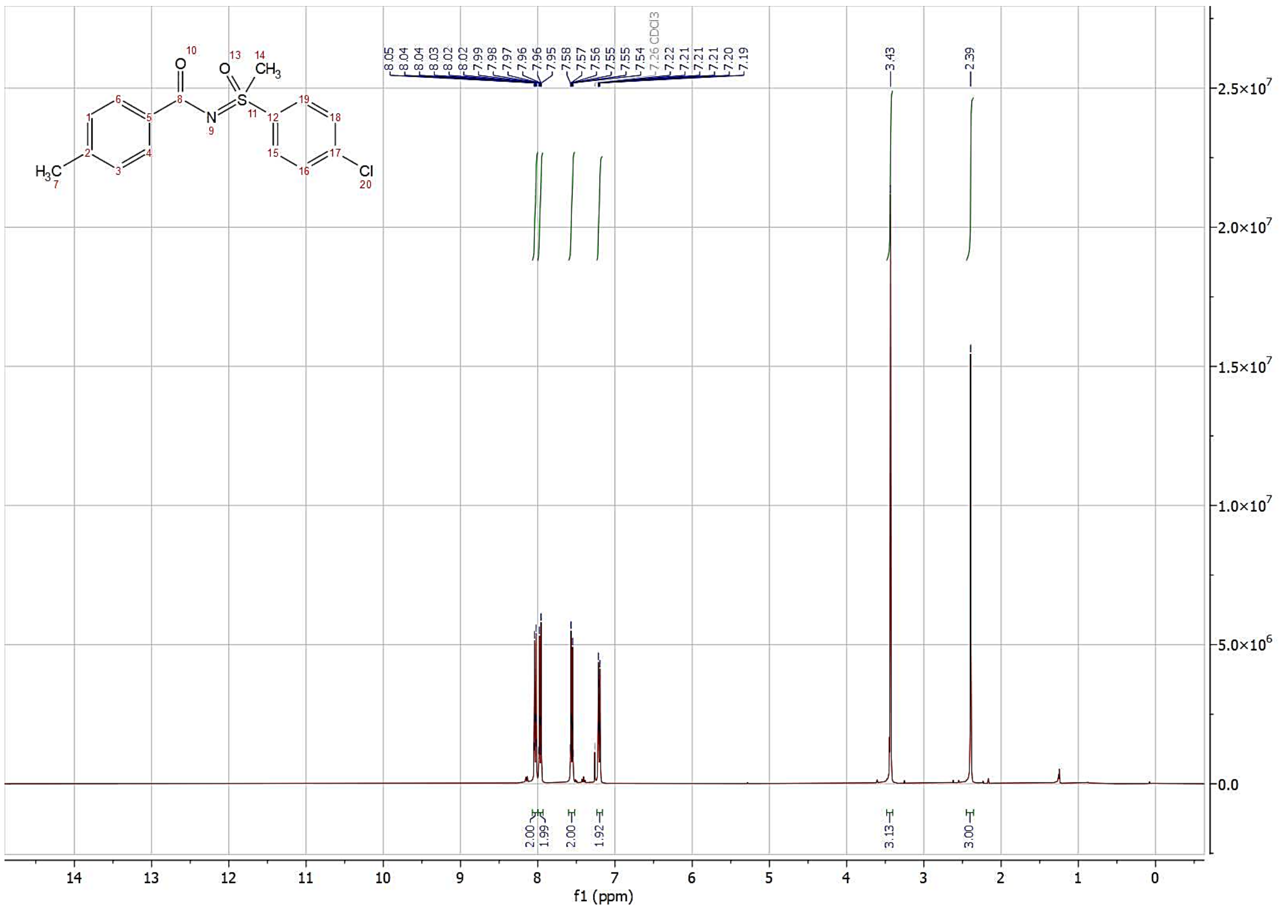

8.05 8.04 8.04 8.03 8.02 8.02 7.99 7.98 7.97 7.96 7.96 7.95 7.58 7.57 7.56 7.55 7.55 7.54 7.26 CDCl3 7.22 7.21 7.21 7.21 7.20 7.19
3.43
2.39
2.00 1.99 2.00 1.92 3.13 3.00
f1 (ppm)

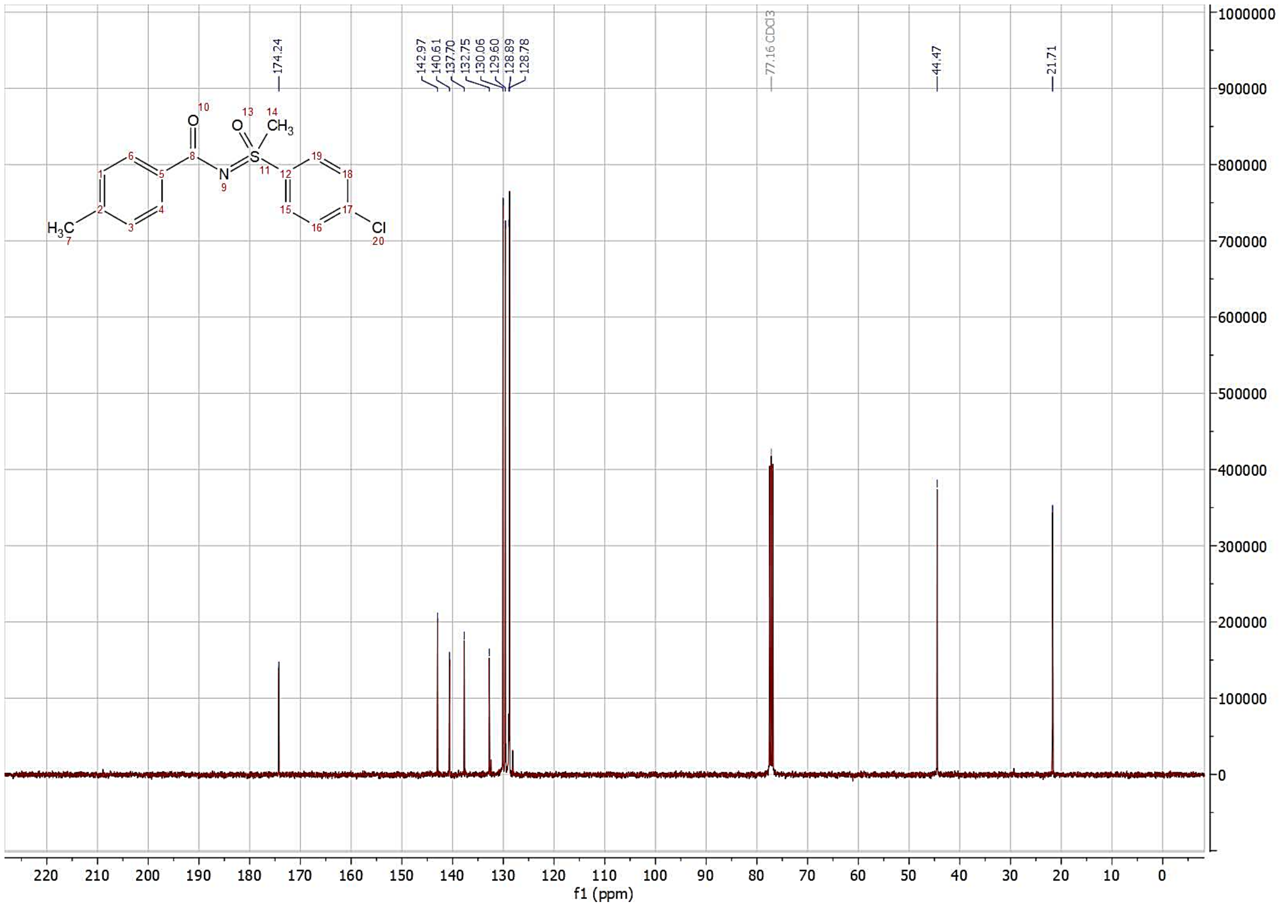

174.24
142.97 140.61 137.70 132.75 130.06 129.60 128.89 128.78
77.16 CDCl3
44.47
21.71
f1 (ppm)

Compound 6i

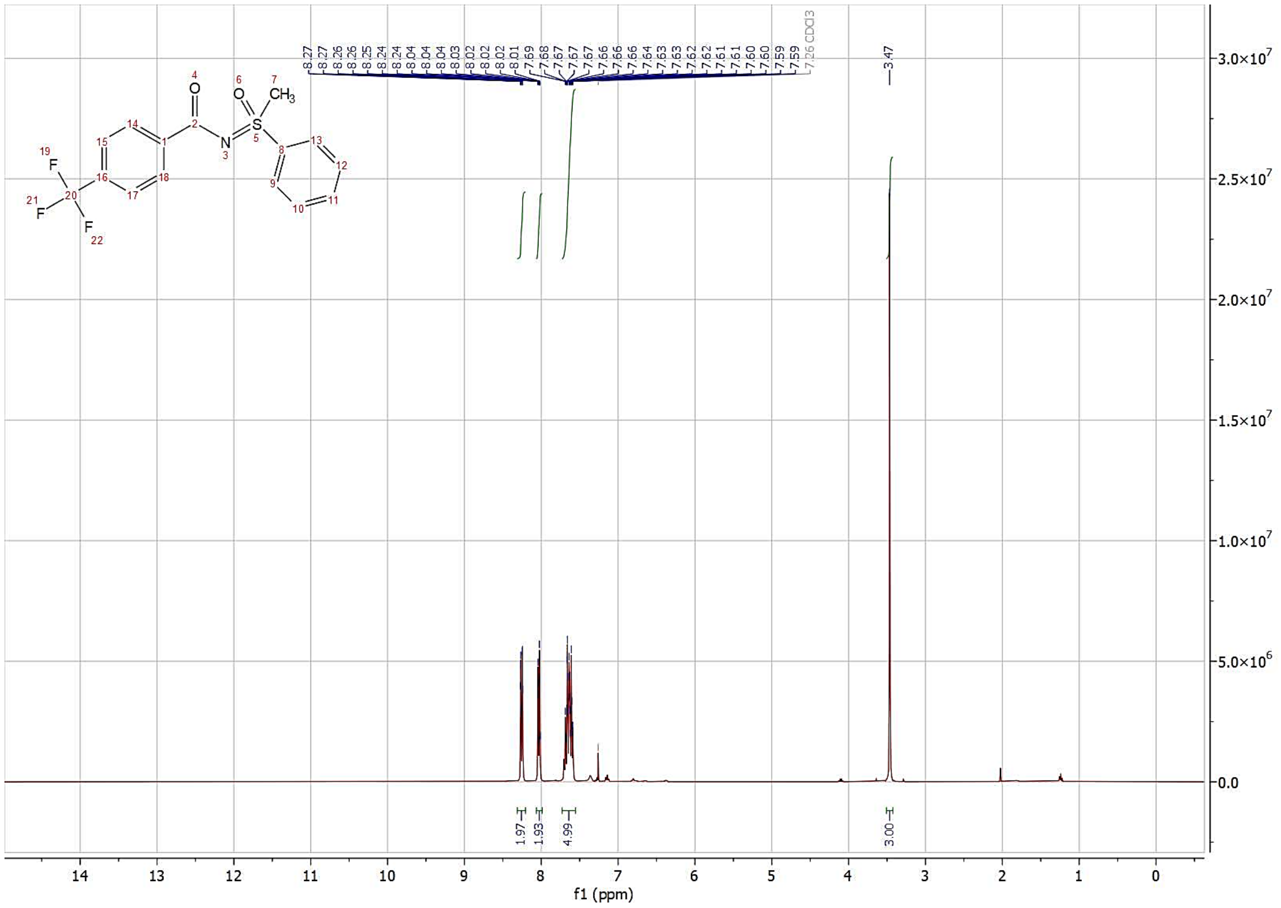
8.27 8.27 8.26 8.26 8.25 8.24 8.24 8.04 8.04 8.04 8.03 8.02 8.02 8.02 8.01 7.69 7.68 7.67 7.67 7.67 7.66 7.66 7.66 7.64 7.63 7.63 7.62 7.62 7.61 7.61 7.60 7.60 7.59 7.59 7.26 CDCl3
3.47
1.97
1.93
4.99
3.00
f1 (ppm)

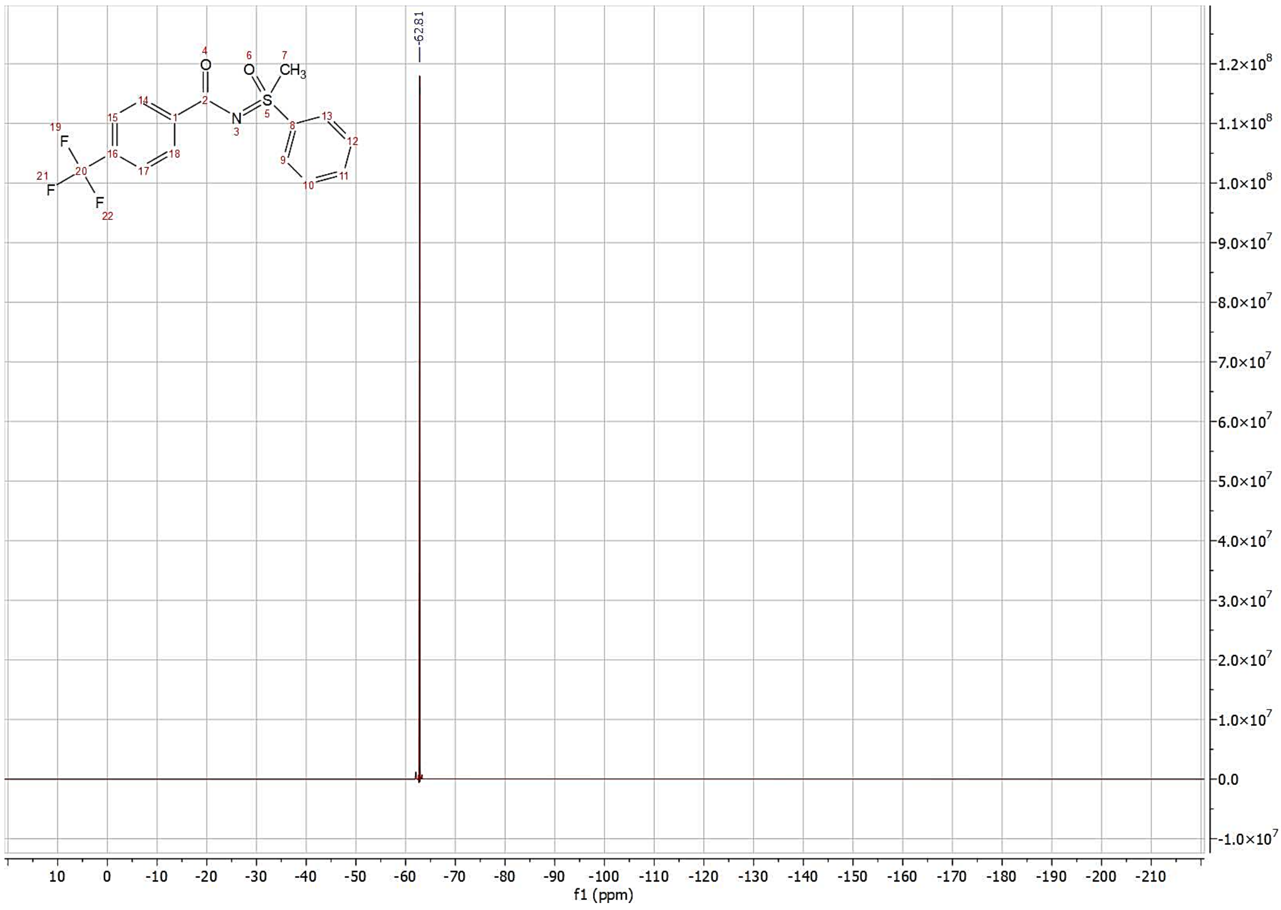
-62.81
f1 (ppm)

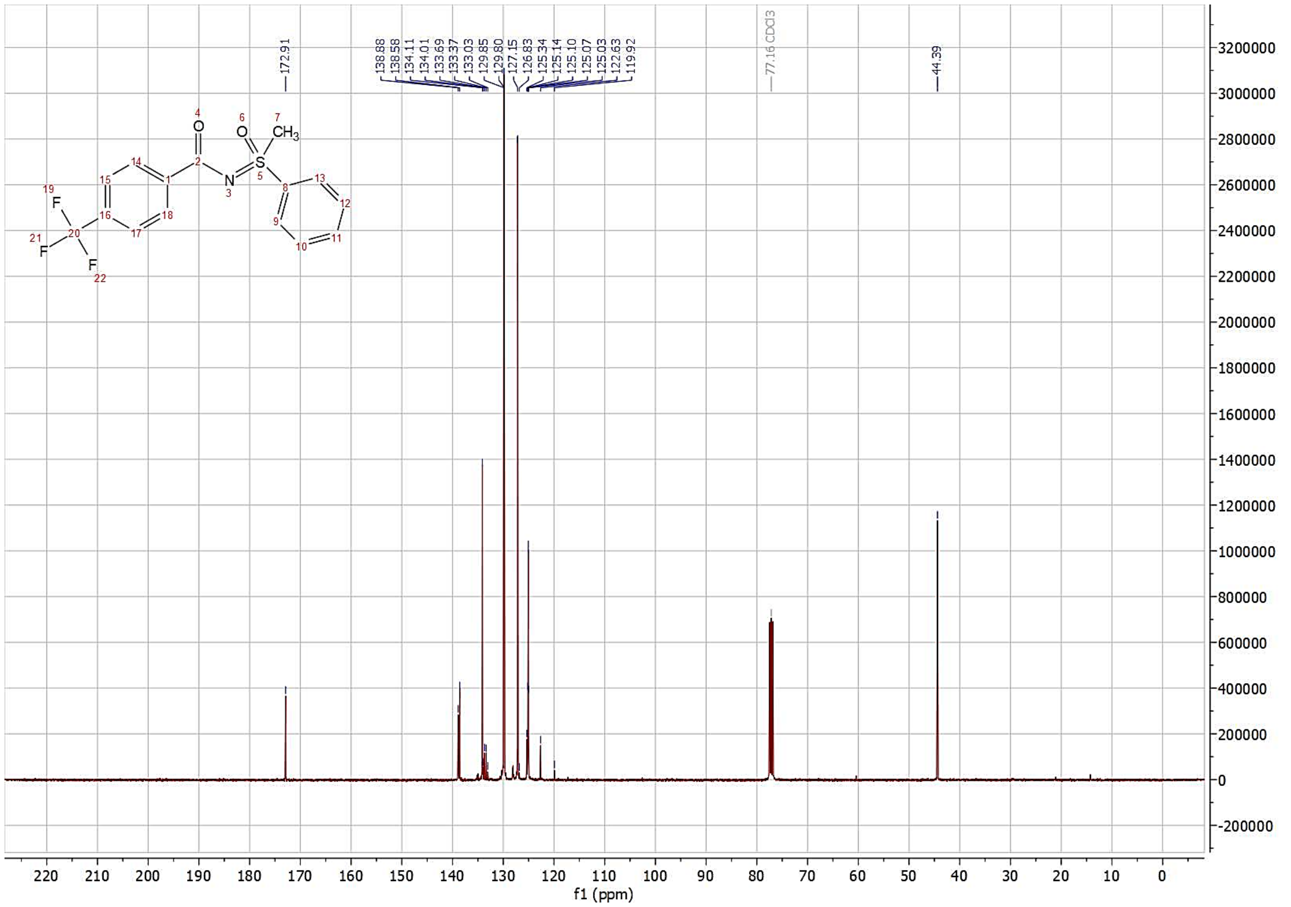
172.91
138.88
138.58
134.11
134.01
133.69
133.37
133.03
129.85
129.80
127.15
126.83
125.34
125.14
125.10
125.07
125.03
122.63
119.92
77.16 CDCl3
44.39
f1 (ppm)

Compound 6j

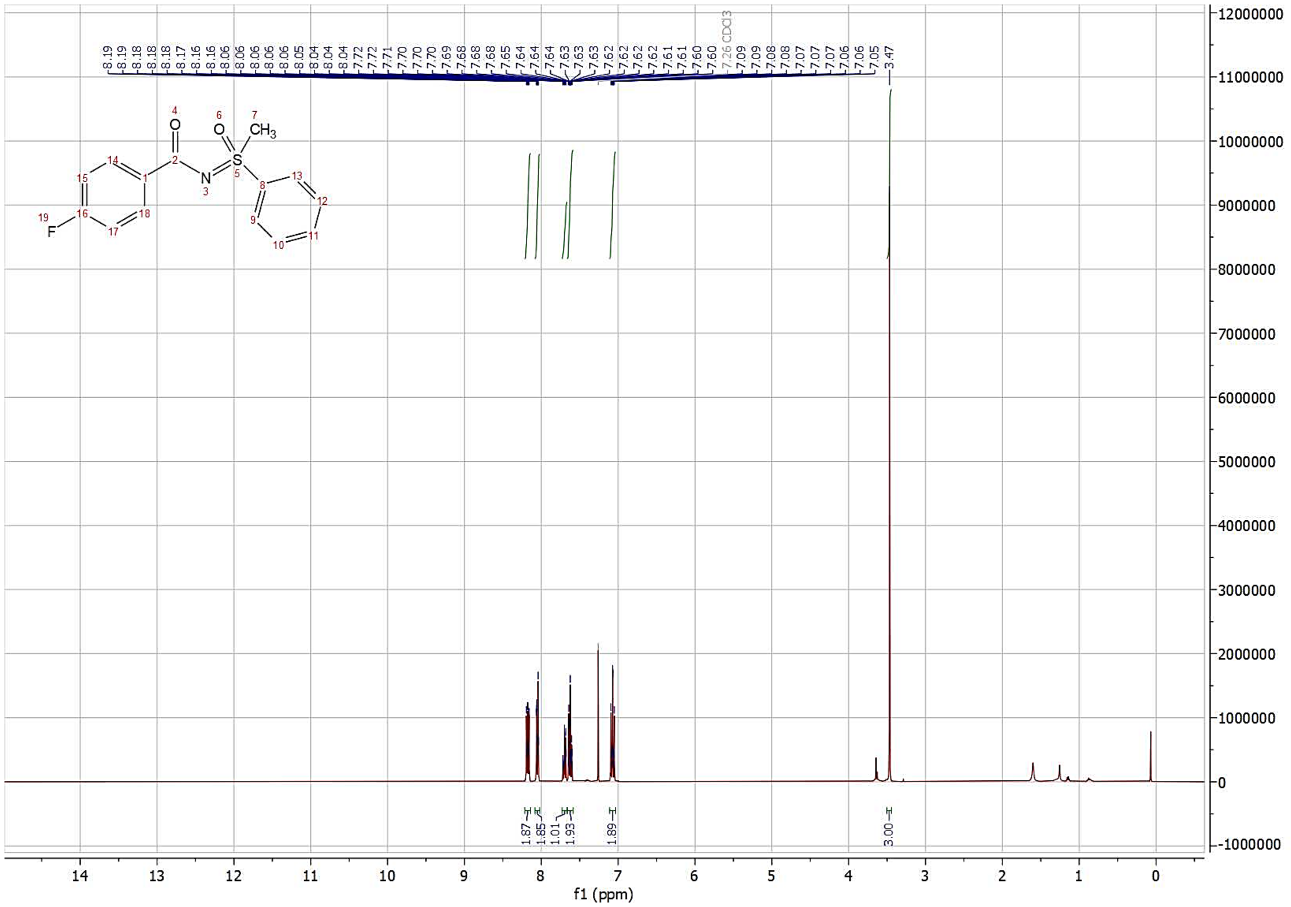
3.47
7.26 CDCl3
1.87
1.85
1.01
1.93
1.89
3.00
f1 (ppm)

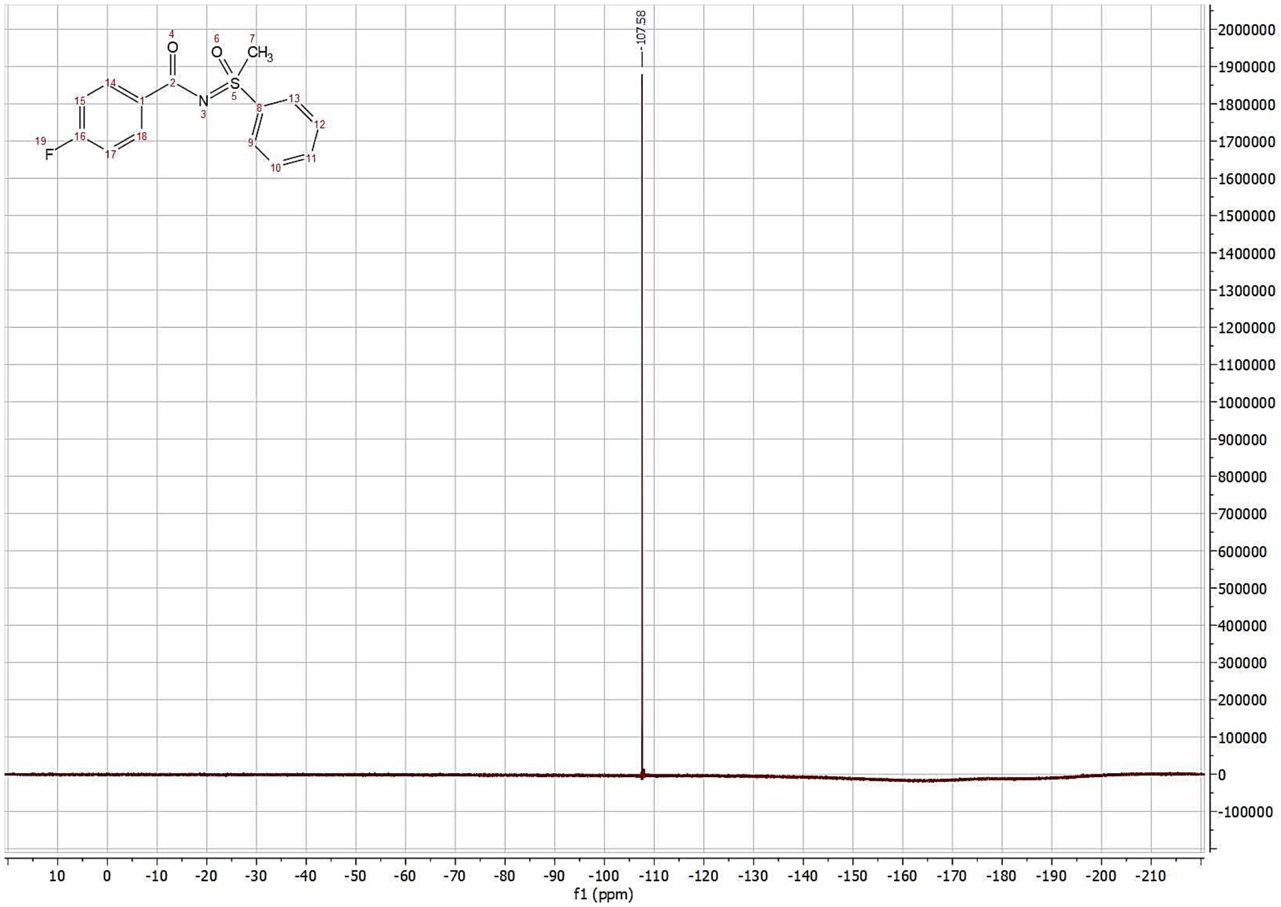
-107.58
f1 (ppm)

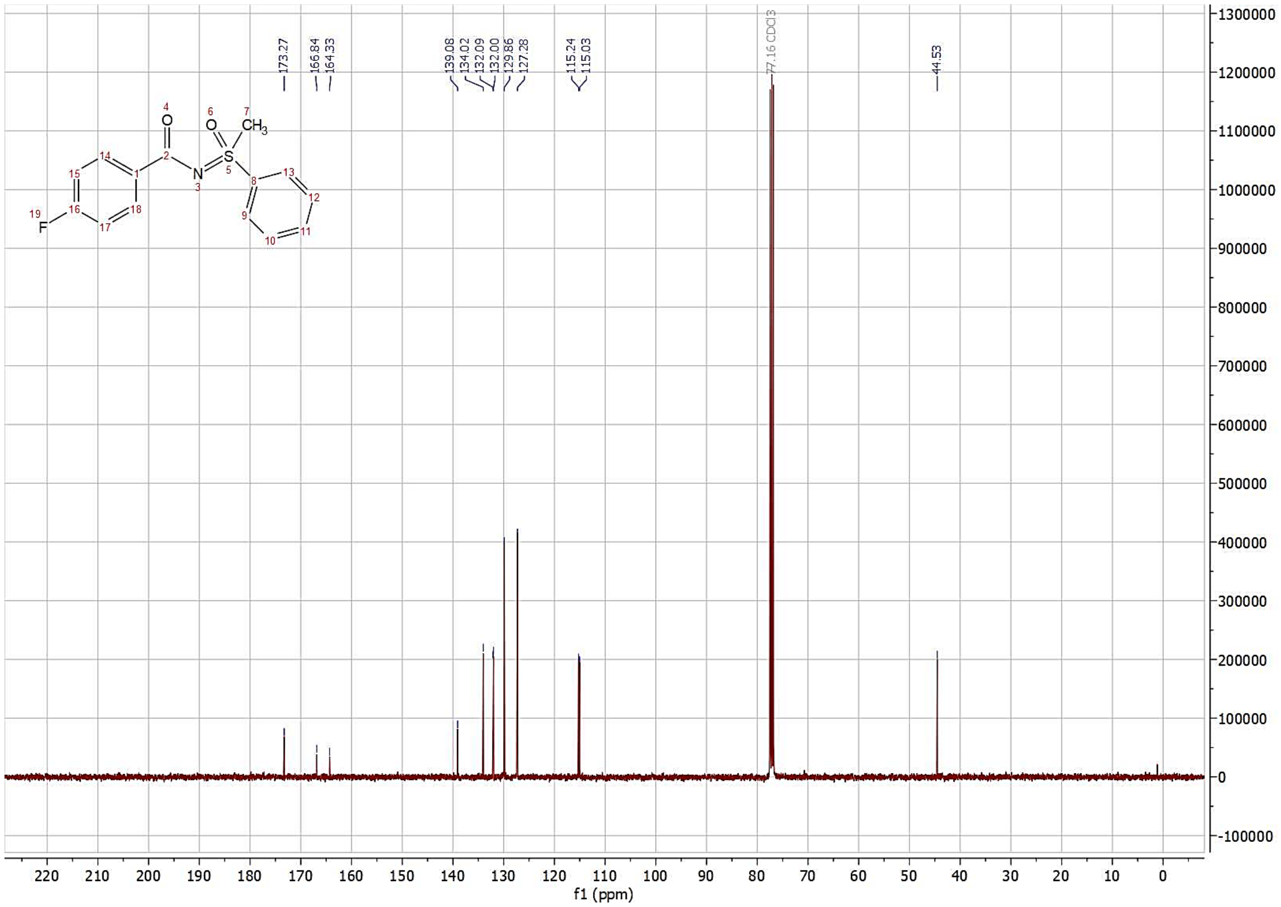
173.27
166.84
164.33
139.08
134.02
132.09
132.00
129.86
127.28
115.24
115.03
77.16 CDCl3
44.53
f1 (ppm)

Compound 6k

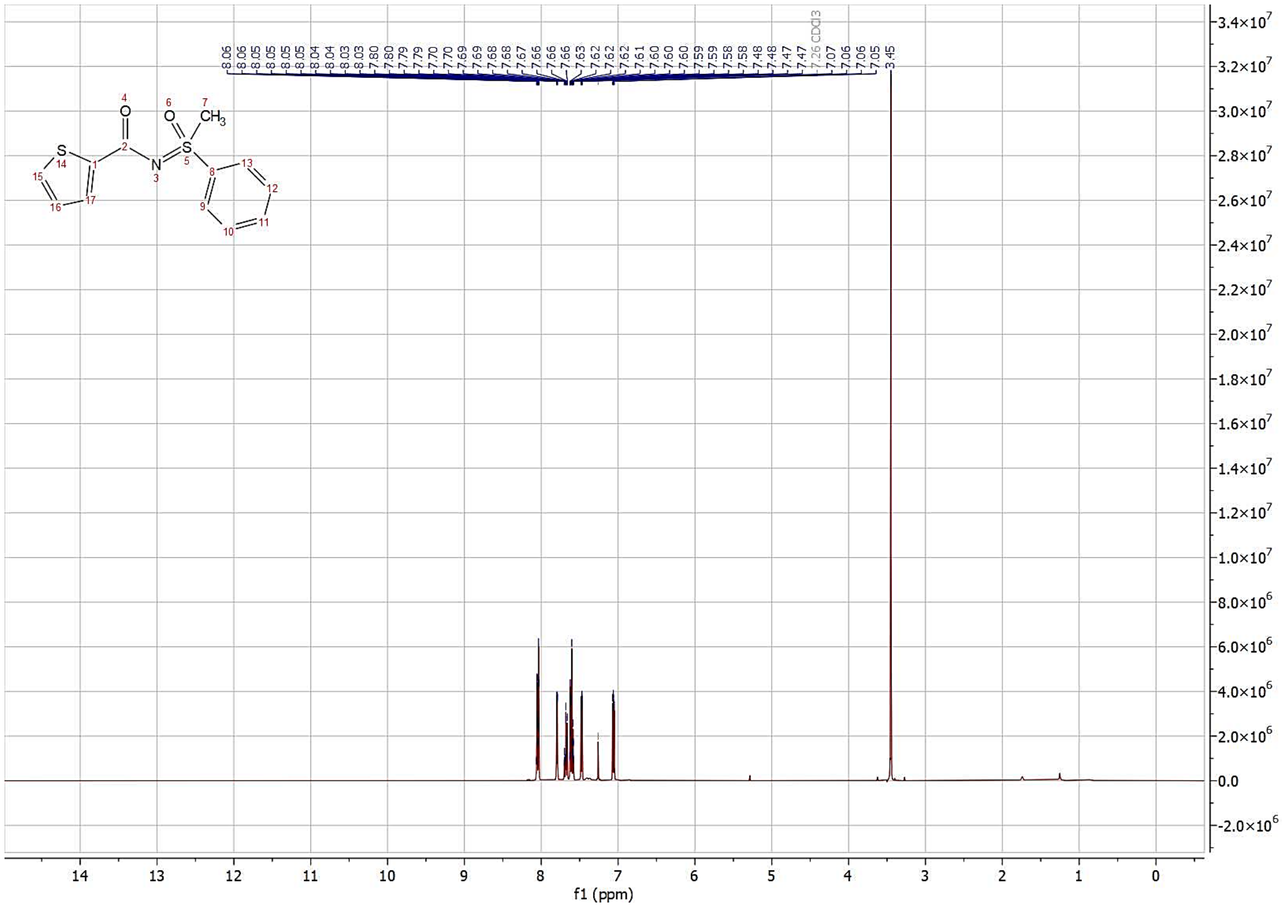
8.06
8.06
8.05
8.05
8.05
8.04
8.04
8.03
8.03
7.80
7.80
7.79
7.79
7.70
7.70
7.69
7.69
7.68
7.67
7.66
7.66
7.66
7.63
7.62
7.62
7.62
7.61
7.60
7.60
7.60
7.59
7.59
7.58
7.58
7.48
7.48
7.47
7.47
7.26 CDCl3
7.07
7.06
7.06
7.05
3.45
f1 (ppm)

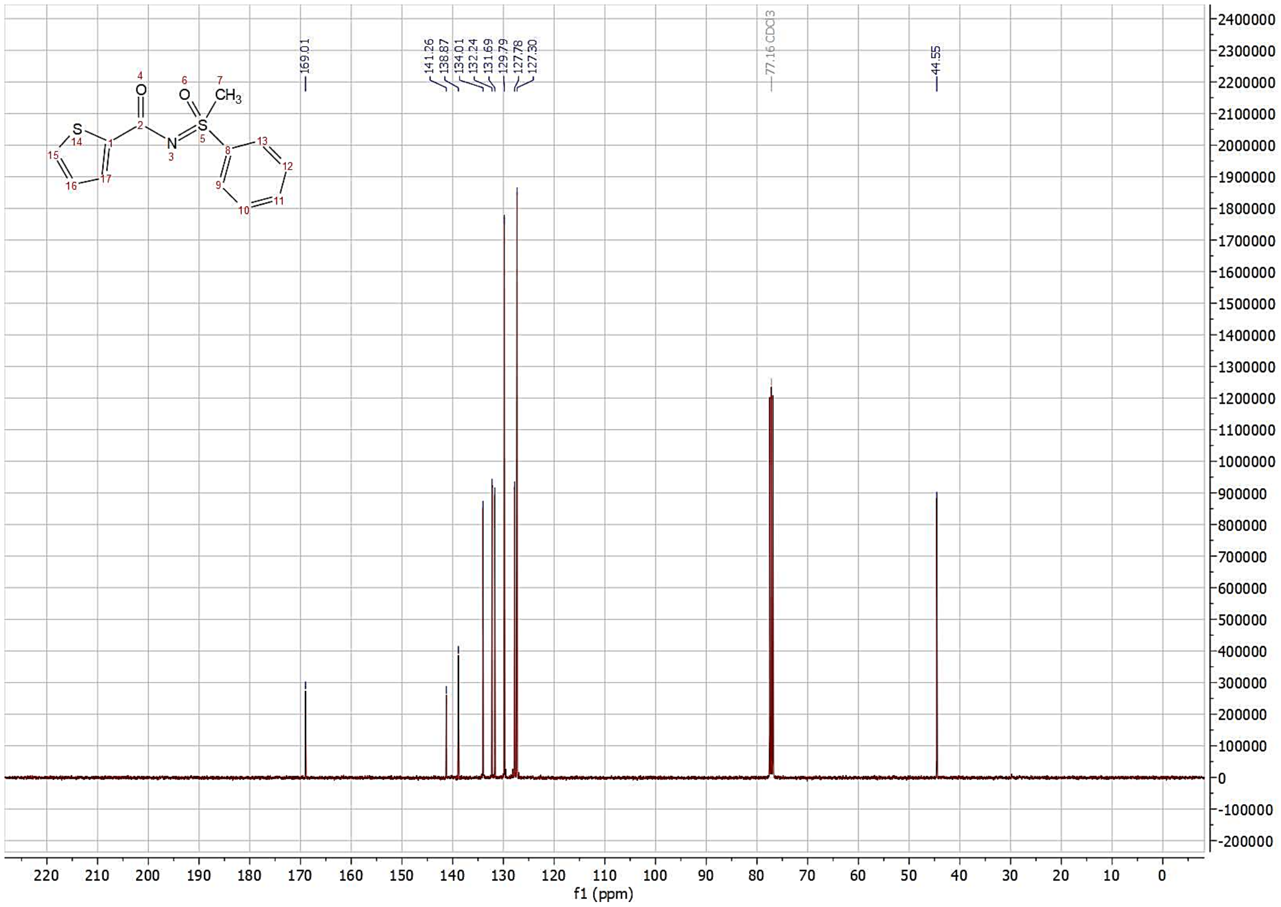
169.01
141.26
138.87
134.01
132.24
131.69
129.79
127.78
127.30
77.16 CDCl3
44.55
f1 (ppm)

Compound 6l

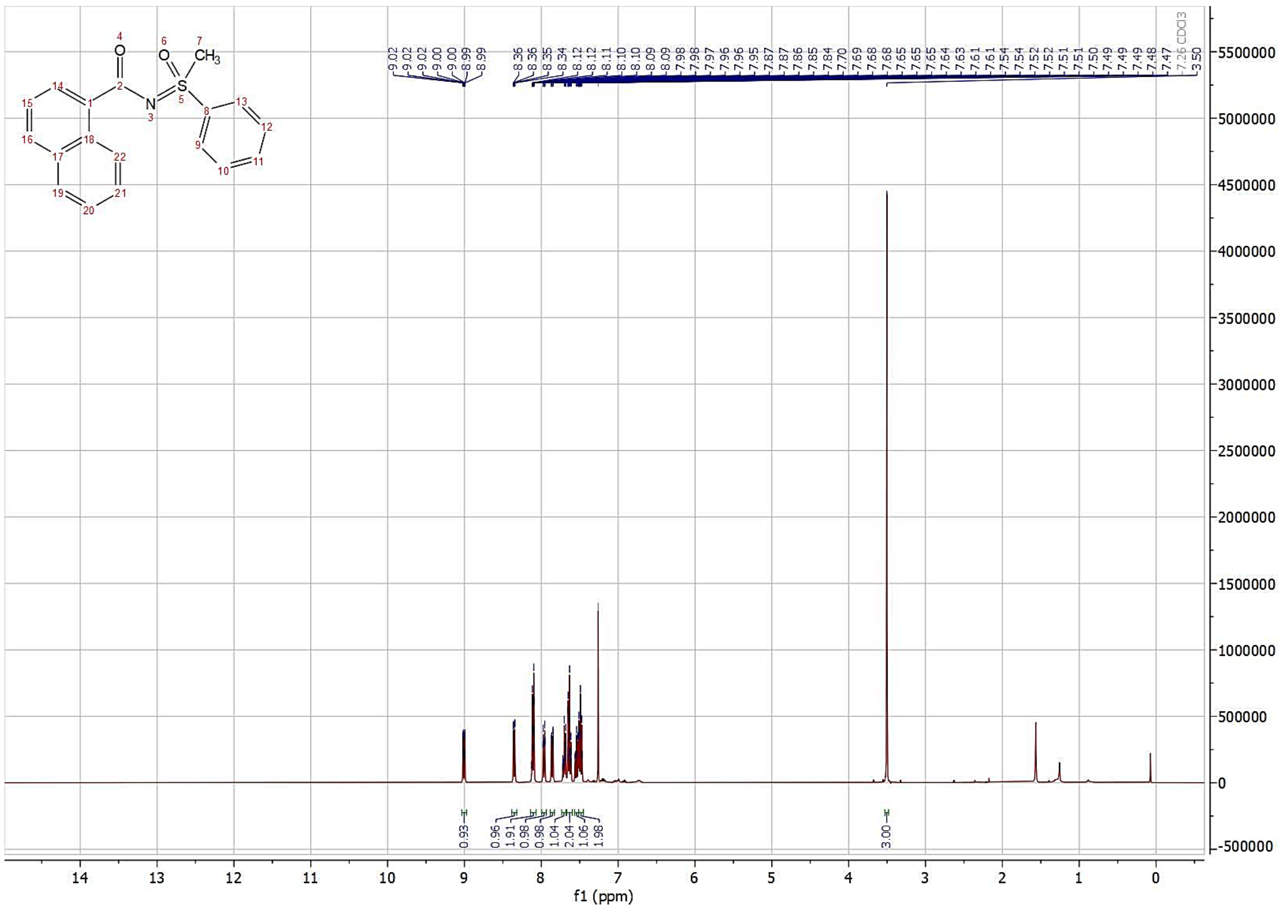

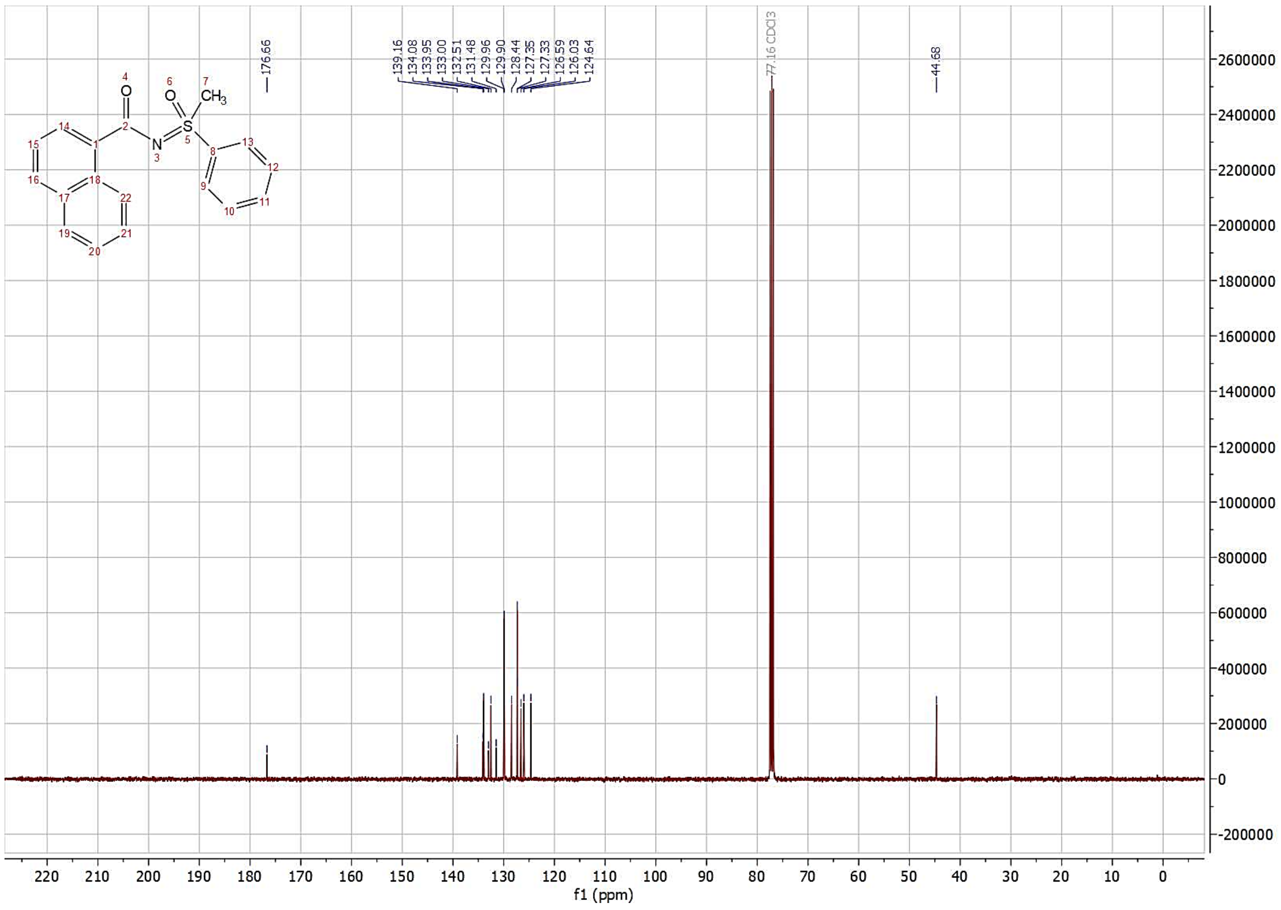

Compound 6m

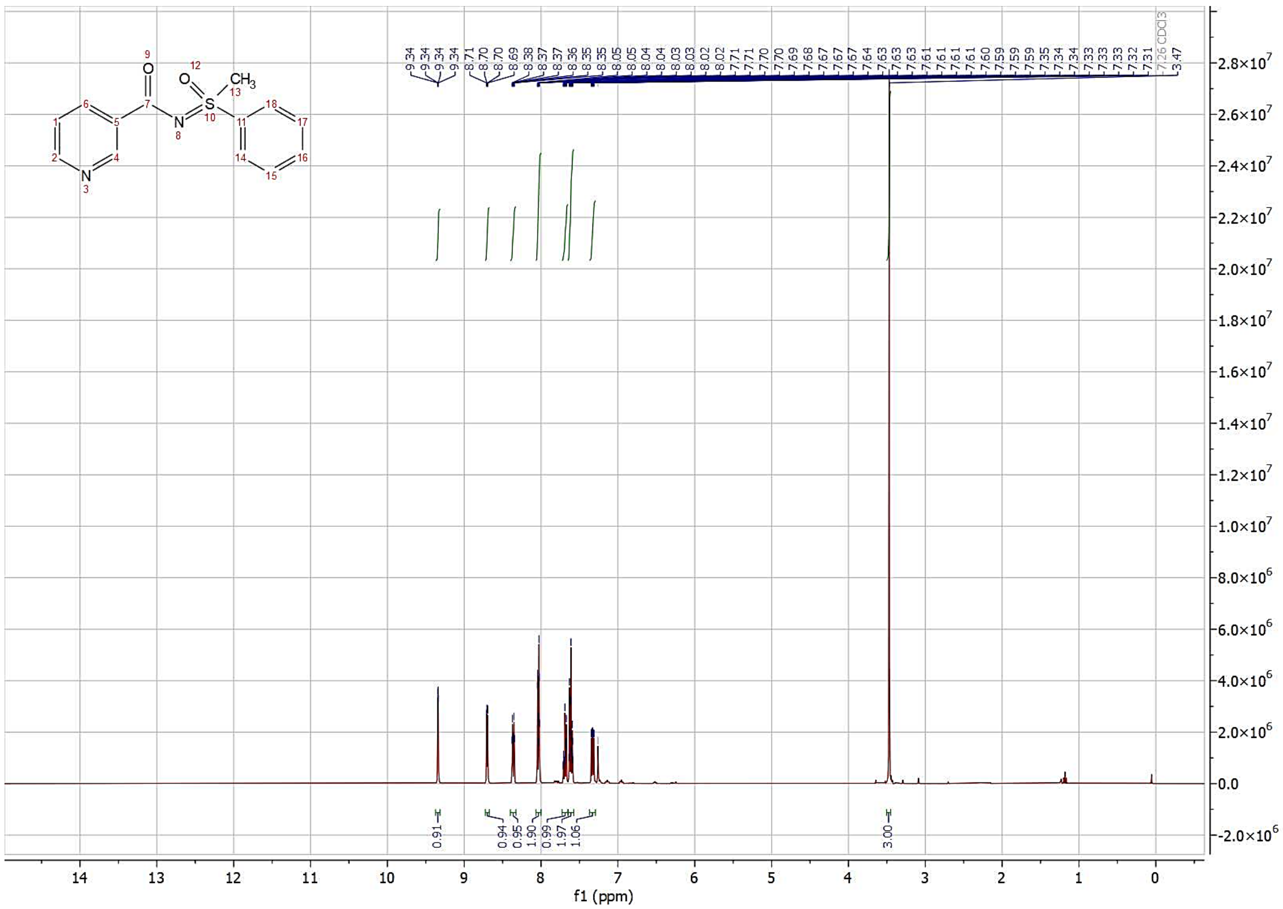

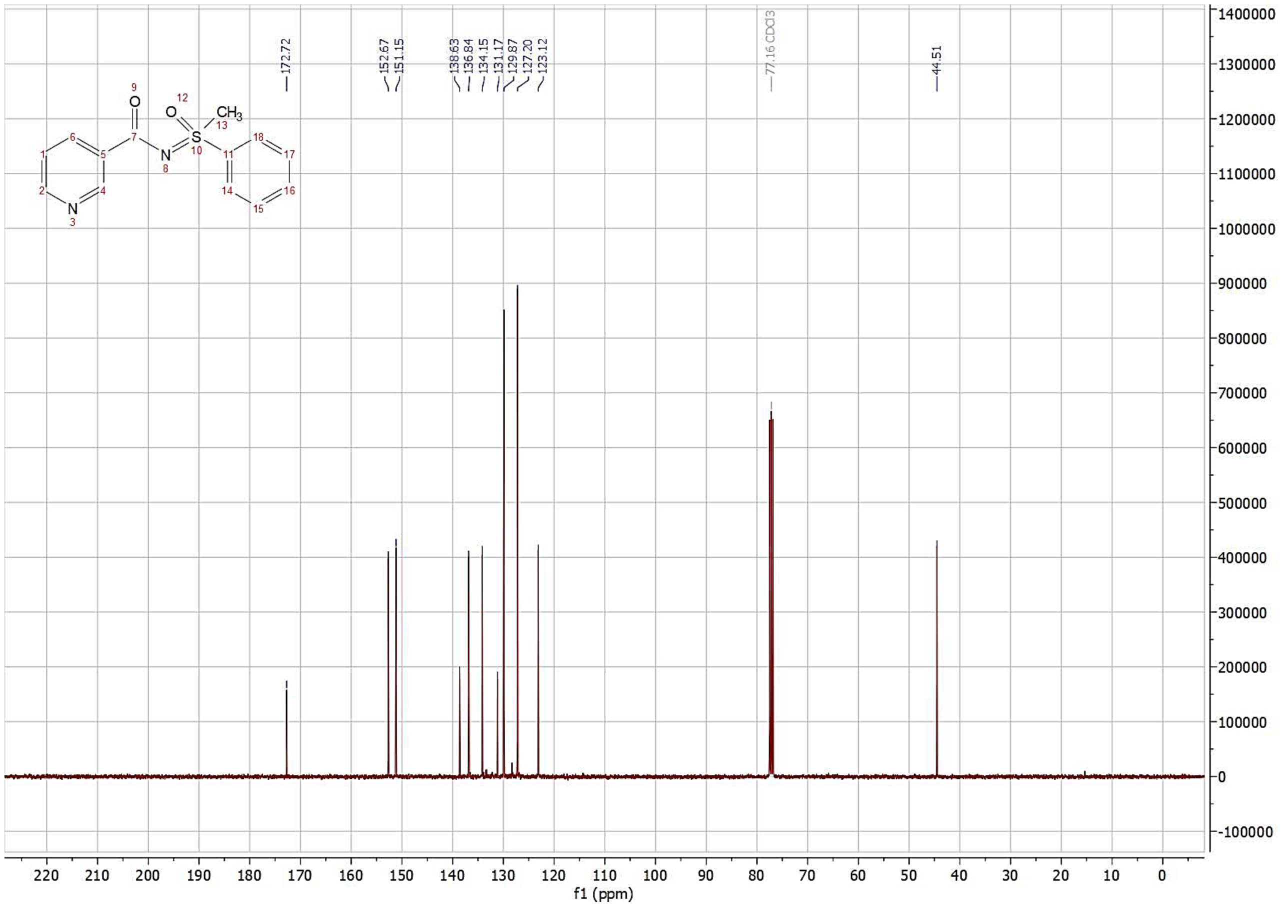

Compound 6n

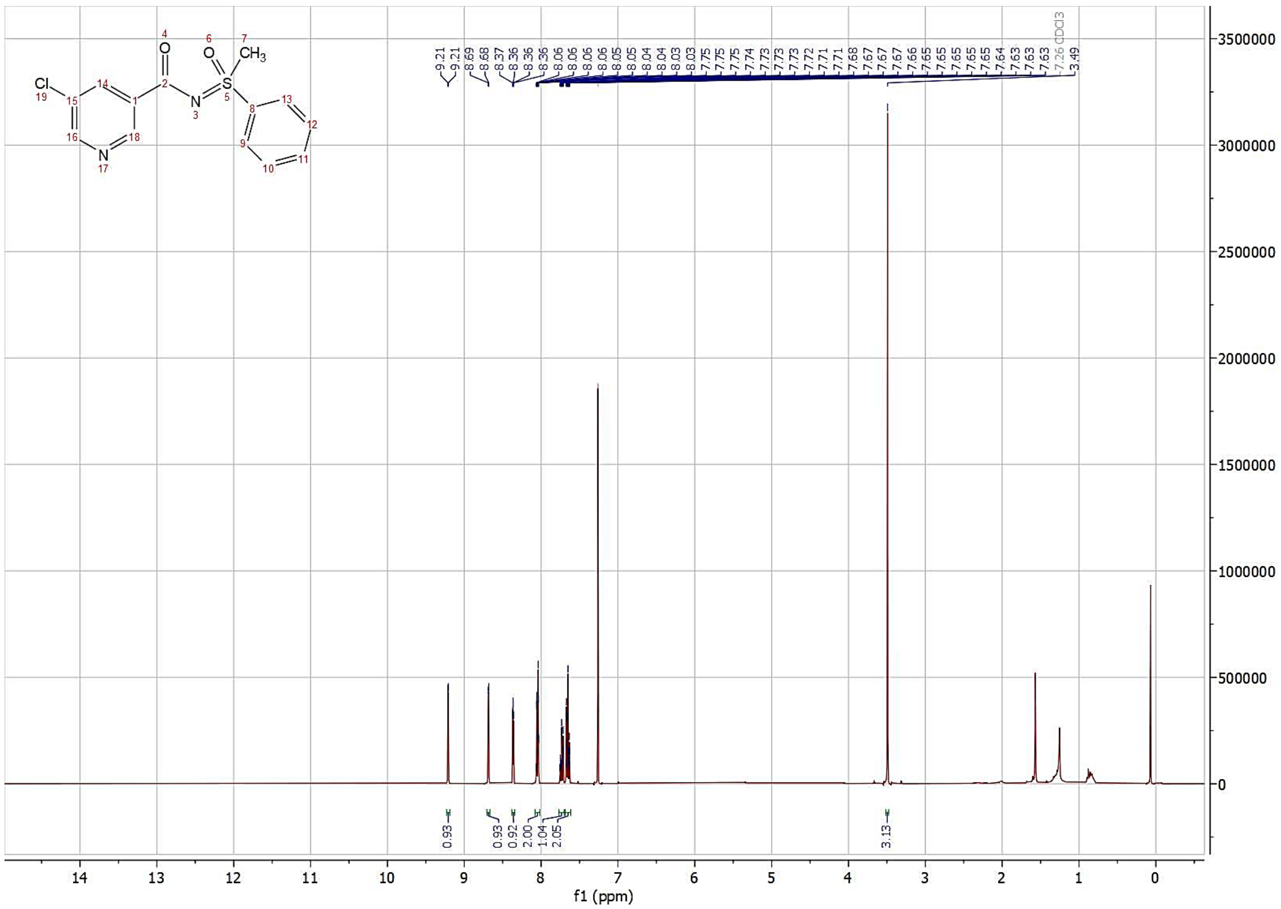
9.21
9.21
8.69
8.68
8.37
8.36
8.36
8.36
8.06
8.06
8.06
8.06
8.05
8.05
8.04
8.03
8.03
7.75
7.75
7.75
7.74
7.73
7.73
7.73
7.72
7.71
7.71
7.68
7.67
7.67
7.67
7.66
7.65
7.65
7.65
7.65
7.64
7.63
7.63
7.63
7.26 CDCl3
3.49
0.93
0.93
0.92
2.00
1.04
2.05
3.13
f1 (ppm)

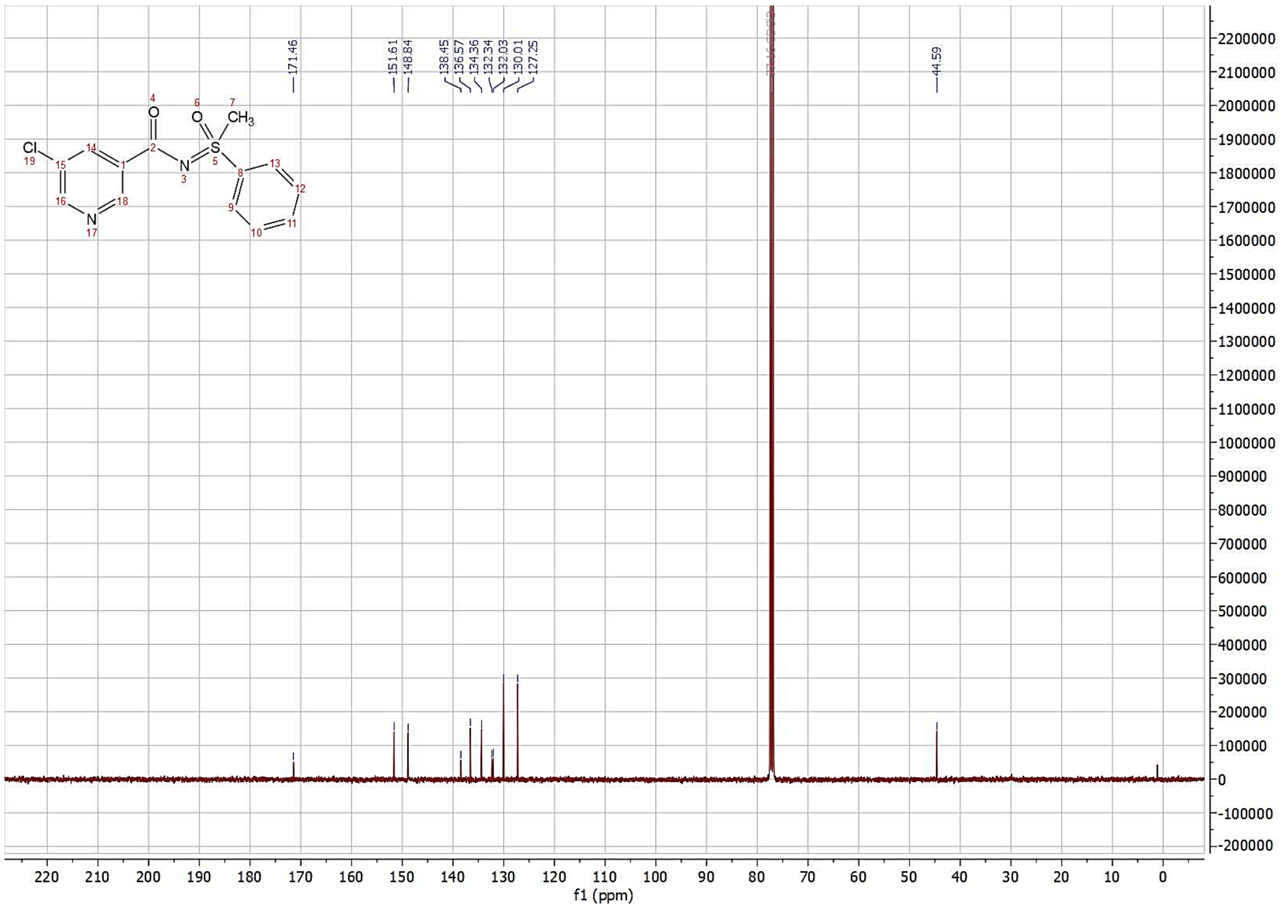
171.46
151.61
148.84
138.45
136.57
134.36
132.34
132.03
130.01
127.25
44.59
f1 (ppm)

Compound 6o

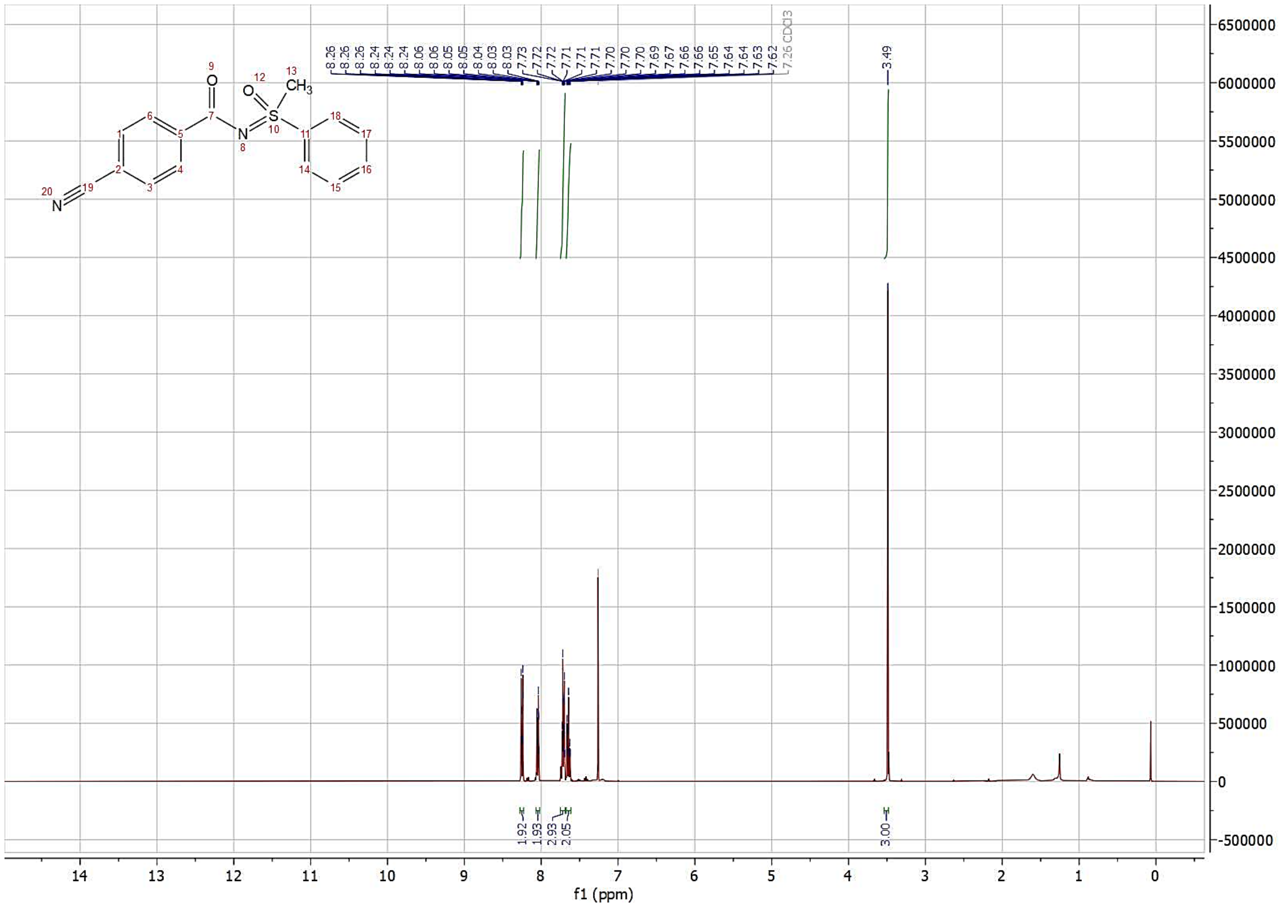

8.26 8.26 8.26 8.24 8.24 8.24 8.06 8.06 8.05 8.05 8.04 8.03 8.03 7.73 7.72 7.72 7.71 7.71 7.71 7.70 7.70 7.69 7.67 7.66 7.65 7.64 7.64 7.63 7.62
7.26 CDCl3
3.49
1.92 1.93 2.93 2.05 3.00
f1 (ppm)

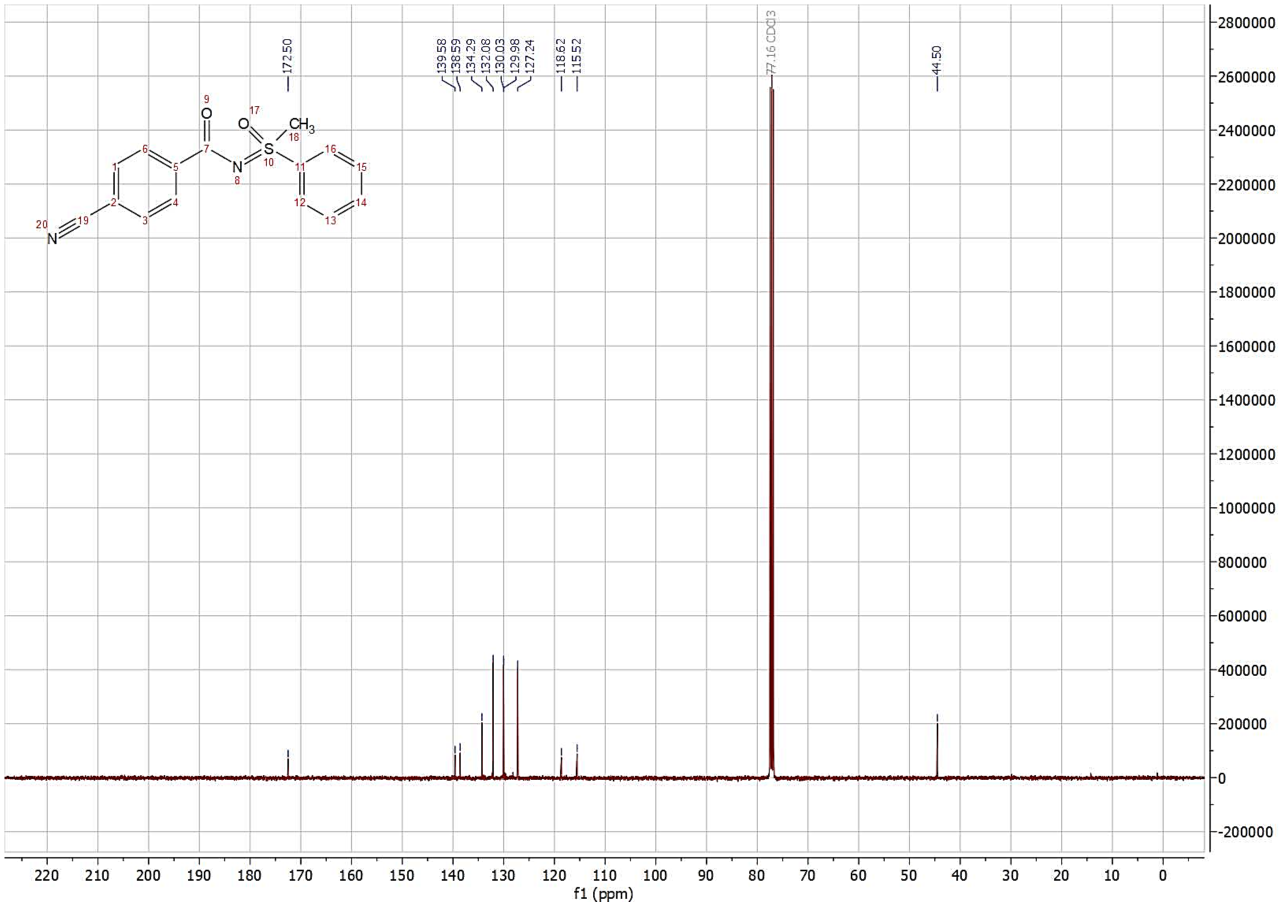

172.50
139.58 138.59 134.29 132.08 130.03 129.98 127.24
118.62 115.52
77.16 CDCl3
44.50
f1 (ppm)

Compound 6p

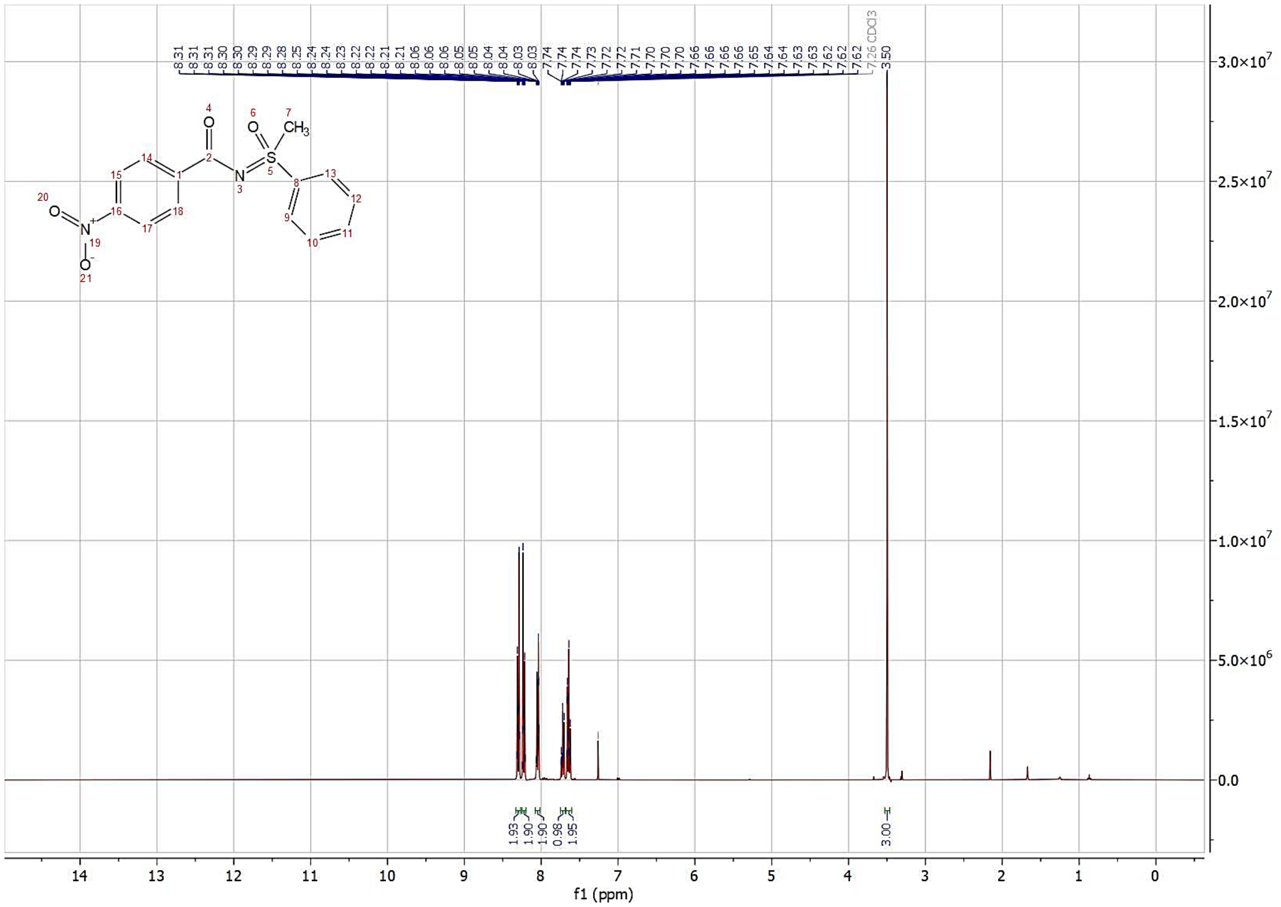

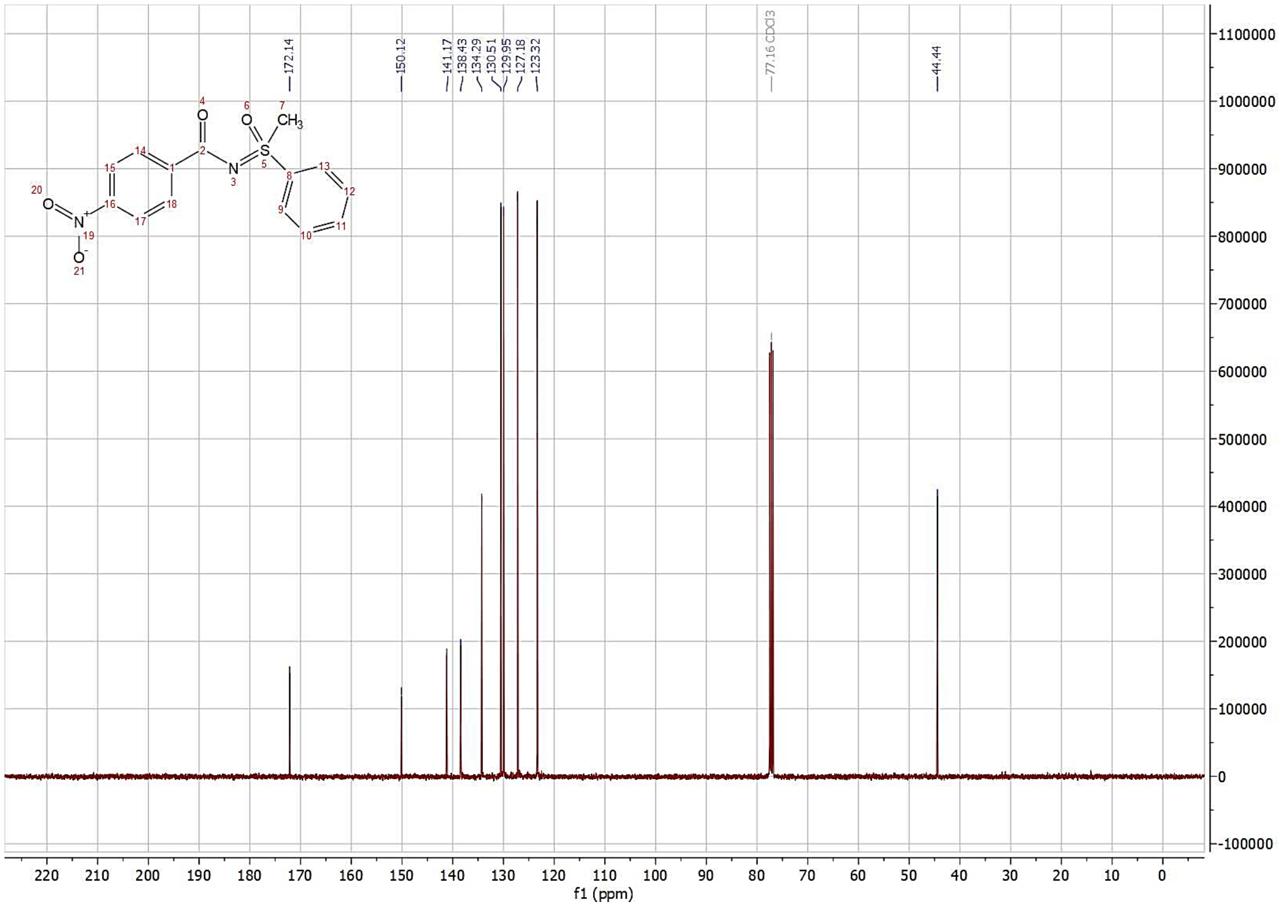

Compound 6q

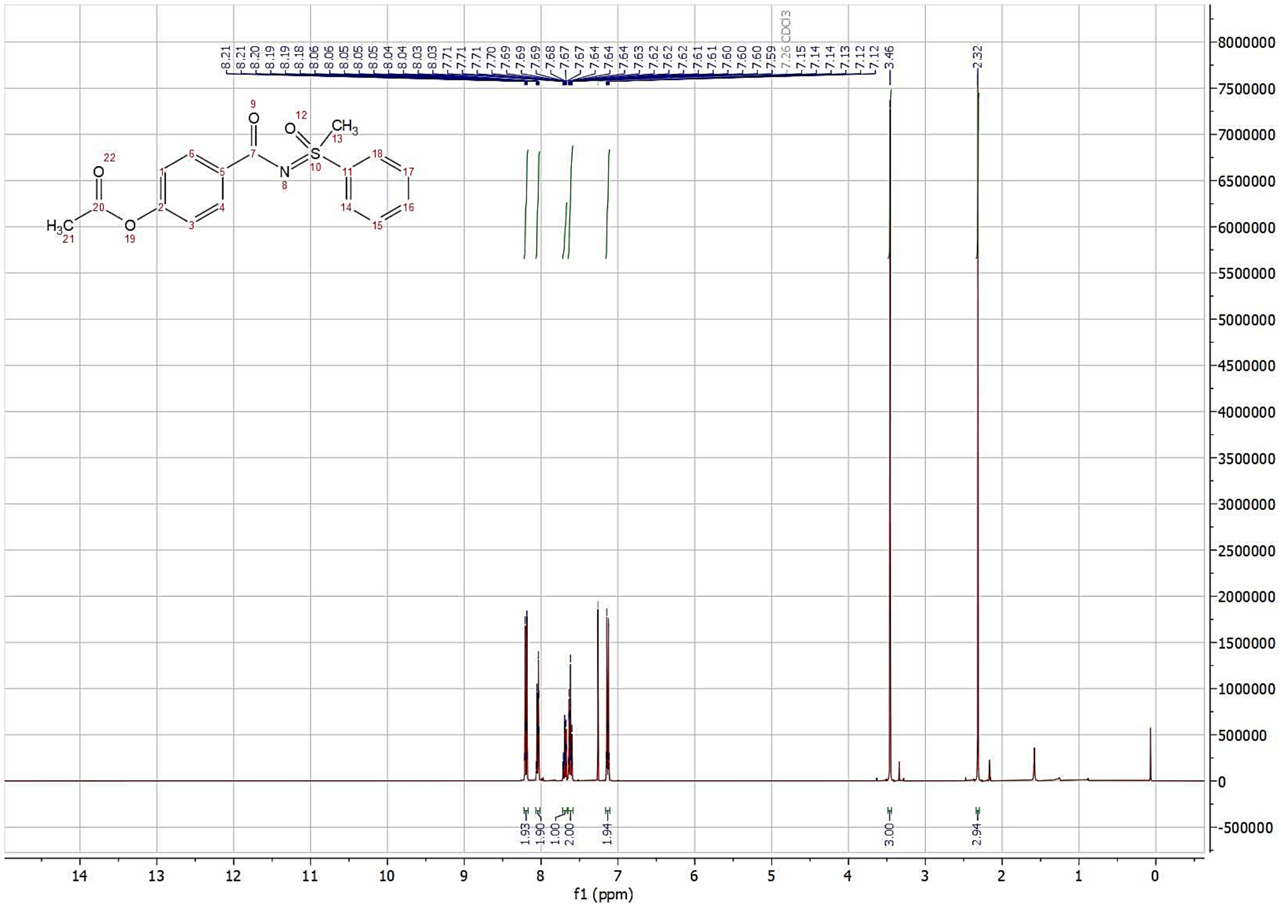

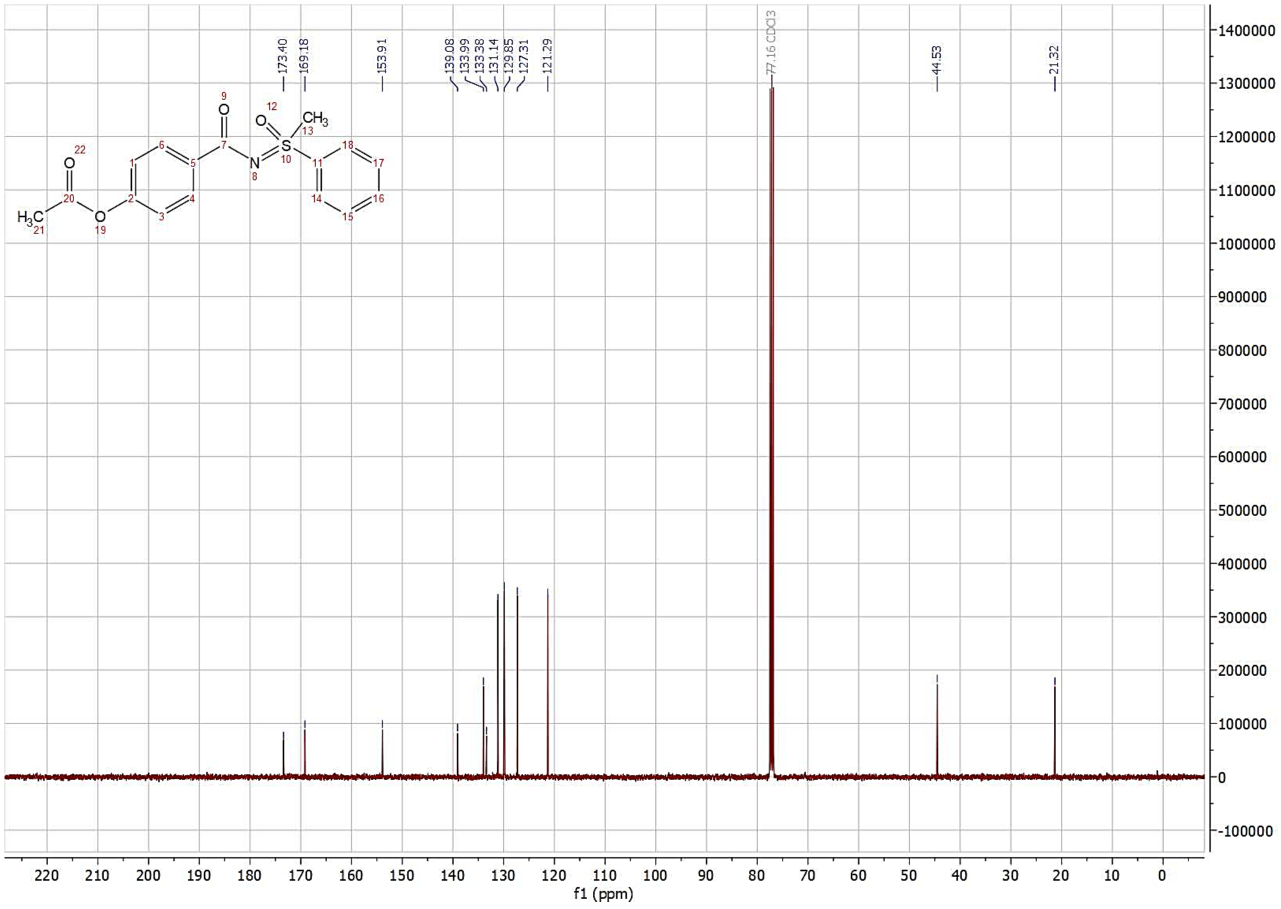

Compound 6r

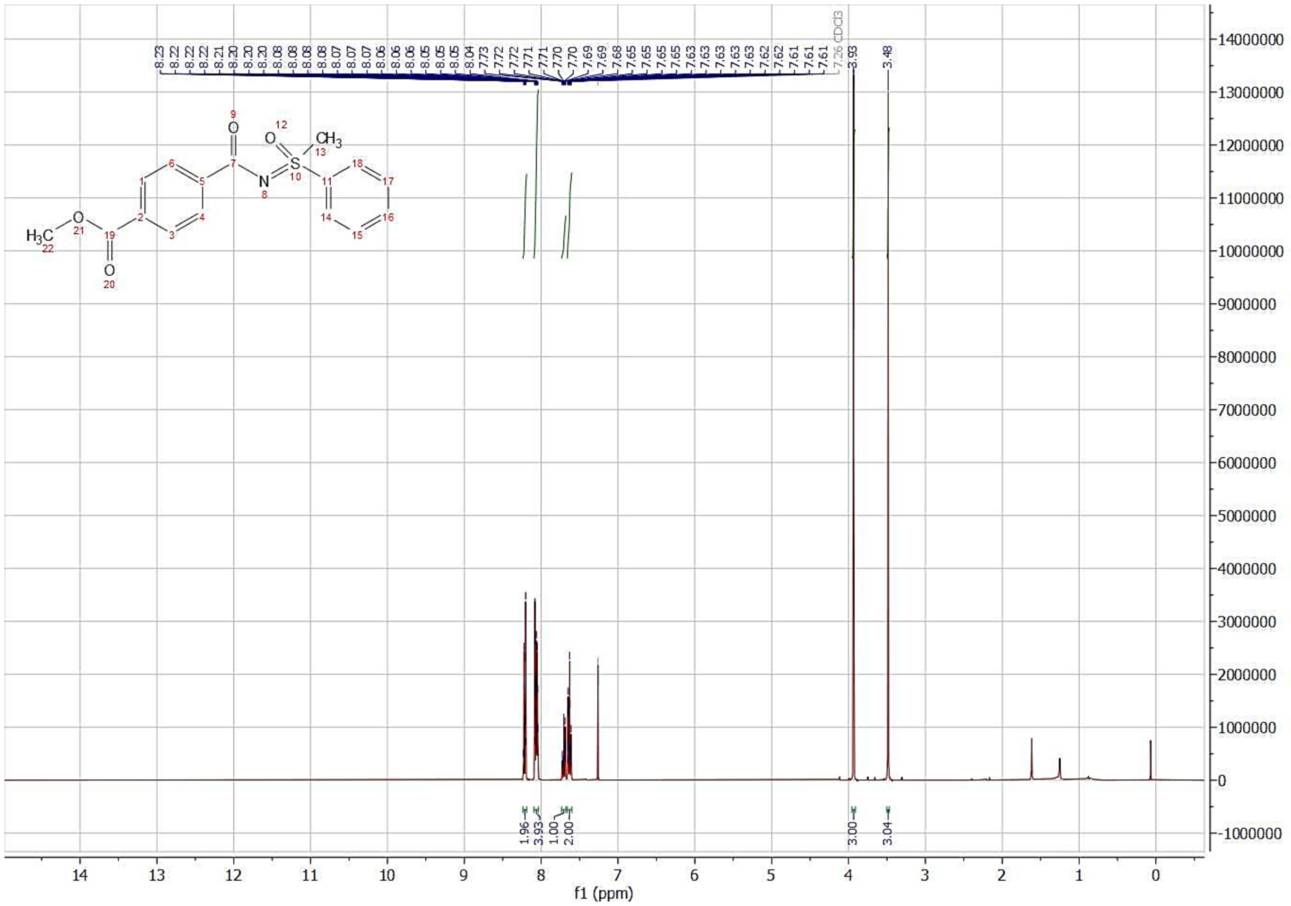

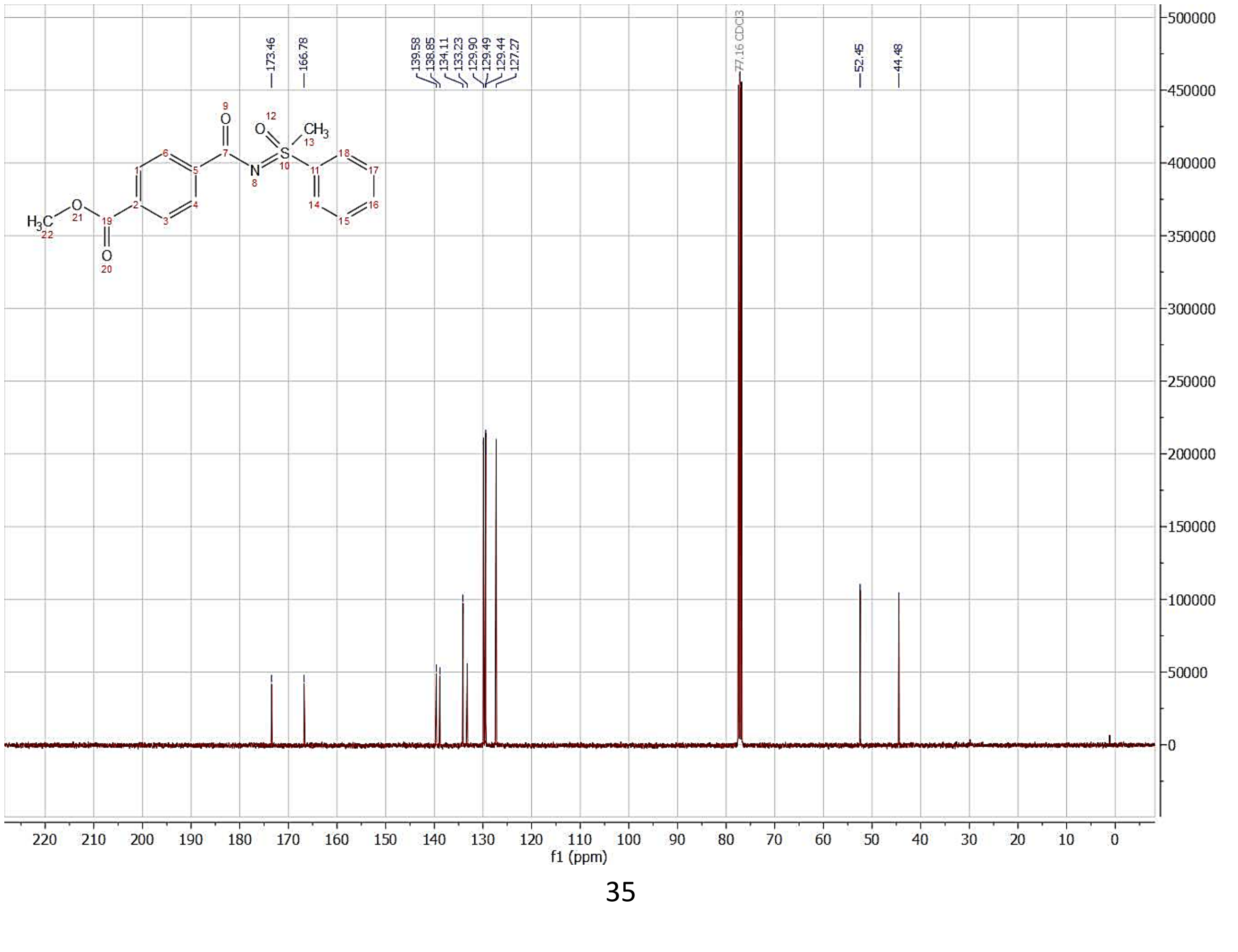

Compound 6s

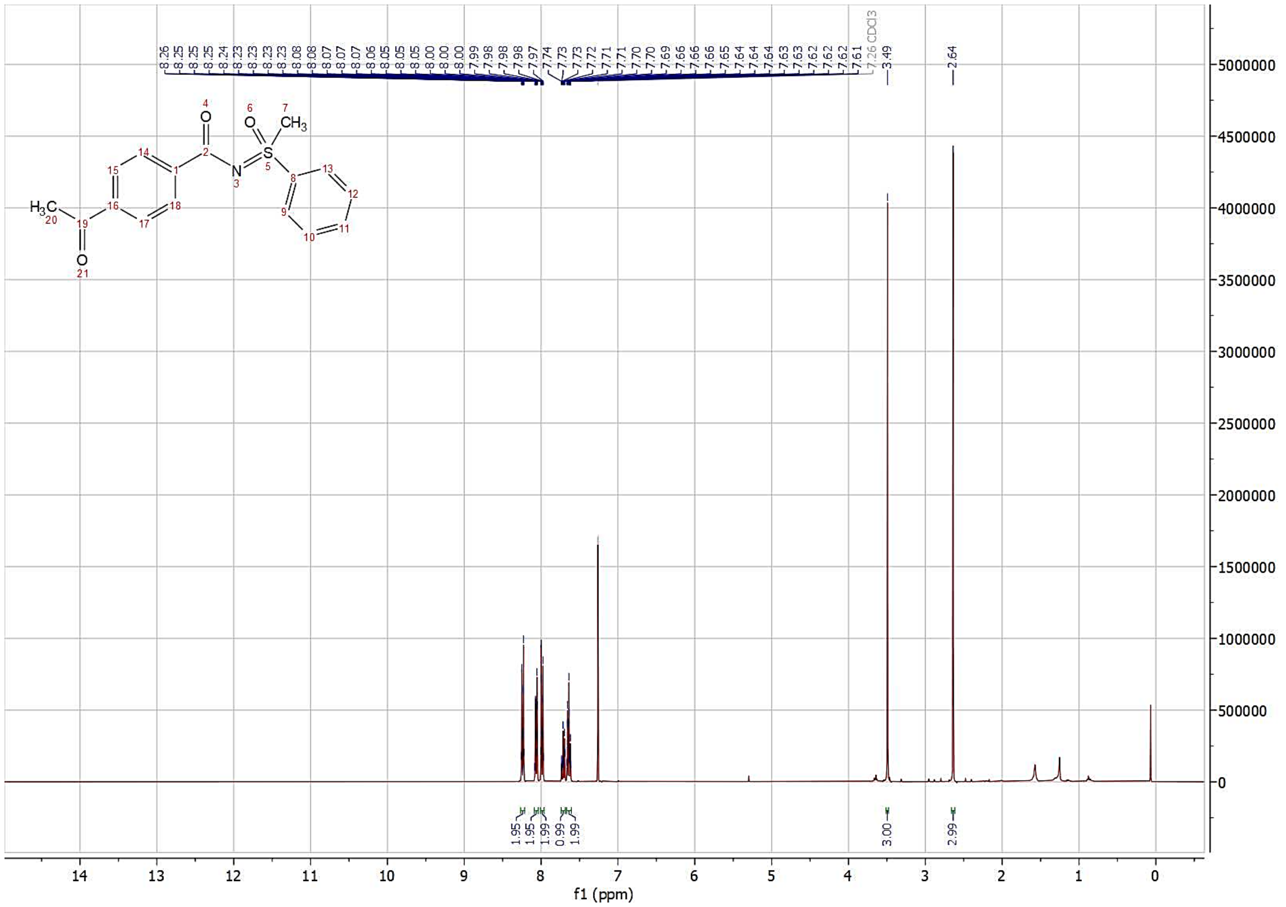

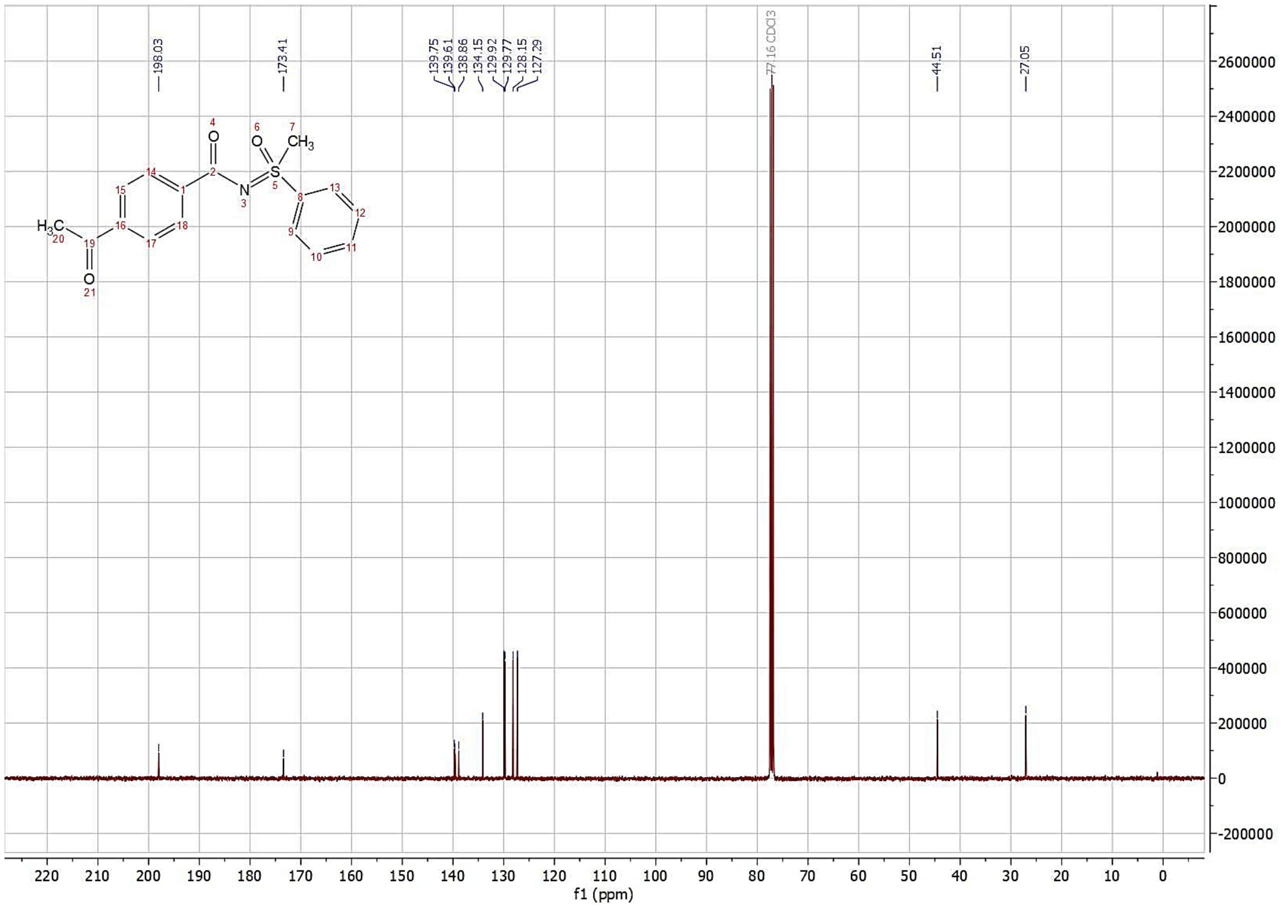

7. References

[6] L. Degennaro, A. Tota, S. De Angelis, M. Andresini, C. Cardellicchio, M. A. Capozzi, G. Romanazzi, R. Luisi, "A Convenient, Mild, and Green Synthesis of NH-Sulfoximines in Flow Reactors" *European Journal of Organic Chemistry* **44**, (2017), 6486.

[38] S.-R. Guo, P. Santhosh Kumar, Y.-Q. Yuan, M.-H. Yang, "Palladium catalyzed aroylation of NH-sulfoximines with aryl halides using chloroform as the CO precursor" *Tetrahedron Letters* **58**, (2017), 2681.

[39] N. Sharma, G. Sekar, "Palladium nanoparticles catalyzed aroylation of NH-sulfoximines with aryl iodides" *RSC Advances* **6**, (2016), 37226.

[40] B. D. Bala, N. Sharma, G. Sekar, "Sulfoximinocarbonylation of aryl halides using heterogeneous Pd/C catalyst" *RSC Advances* **6**, (2016), 97152.

[77] K. Stagel, K. Rath, P. M. Kathe, M. Schnürch, T. M. Huber, A. K. Opitz, K. Bica-Schröder, "Online Coupling High-Temperature Electrolysis with Carbonylation Reactions: A Powerful Method for Continuous Carbon Dioxide Utilization" *Angewandte Chemie International Edition* **64**, (2025), e202420578.

[78] C. Li, Y. Yang, X. Zheng, C. Zhang, H. Cai, W. Lin, "Photocatalyzed sulfoximination/amidation of (Het)arylethenes tethered N-tosyl amide: a versatile entry to sulfoximidoyl β- and γ-lactams" *Organic Chemistry Frontiers* **11**, (2024), 4508.

[82] A. Nenning, M. Holzmann, J. Fleig, A. K. Opitz, "Excellent kinetics of single-phase Gd-doped ceria fuel electrodes in solid oxide cells" Materials Advances **2**, (2021), 5422.

[83] F. M. S. Rodrigues, L. D. Dias, M. J. F. Calvete, T. M. R. Maria, L. M. Rossi, A. J. L. Pombeiro, L. M. D. R. S. Martins, M. M. Pereira, "Immobilization of Rh(I)-N-Xantphos and Fe(II)-C-Scorpionate onto Magnetic Nanoparticles: Reusable Catalytic System for Sequential Hydroformylation/Acetalization" *Catalysts* **11**, (2021), 608.

[86] B. I. P. Smith, N. M. L. Knight, G. J. Knox, D. M. Lindsay, L. C. Paterson, J. Bergare, C. S. Elmore, R. A. Bragg, W. J. Kerr, "Selective Deuteration and Tritiation of Pharmaceutically Relevant Sulfoximines" Angew. Chem. Int. Ed. **64**, (2025), 64, e202417179.

[87] W. Su, P. Xu, R. Petzold, J. Yan, T. Ritter, "Ligand-to-Copper Charge-Transfer-Enabled C-H Sulfoximination of Arenes" Org. Lett. **25**, (2023), 1025.
